# Multi-omics analyses related to mitochondria and ageing in triple-negative breast cancer implicate PYCR1 potentiates tumor progression

**DOI:** 10.1186/s12935-026-04235-0

**Published:** 2026-02-26

**Authors:** Jia-nan Huang, Jingxi Hu, Chao Shi, Chunyan Chu, Haolin Hu

**Affiliations:** 1 Department of Breast Center, Zhongda Hospital, Medical School, Southeast University, 87 Dingjiaqiao Road, Jiangsu 210009 Nanjing, China; 2 Bachelor of Pharmaceutical Sciences, Monash University, Parkville, 3052 Victoria Australia; 3 Department of General Surgery, Xuyi County People’s Hospital, Xuyi, 211700 Jiangsu China; 4 Department of Pathology, Zhongda Hospital, Medical School, Southeast University, 87 Dingjiaqiao Road, Jiangsu 210009 Nanjing, China; 5 Department of Breast Center, Zhongda Hospital, Southeast University, 87 Dingjiaqiao Road, Jiangsu 210009 Nanjing, China

**Keywords:** TNBC, Mitochondrial genes, Ageing genes, Prognostic models, PYCR1

## Abstract

**Background:**

Triple-negative breast cancer (TNBC), defined by the lack of expression of Estrogen Receptor (ER), Progesterone Receptor (PR), and Human Epidermal Growth Factor Receptor 2 (HER2), is associated with increased rates of recurrence and mortality. Alterations in energy metabolism often accompany malignant transformation of cells, a process closely linked to mitochondrial function. Ageing contributes to tumor progression through multiple mechanisms. This study aims to explore the mechanisms by which mitochondrial function and ageing influence TNBC, providing new targets and strategies for its diagnosis and treatment.

**Methods:**

This study identified mitochondrial ageing-related differentially expressed genes (MAR-DEGs) and constructed a prognostic prediction model based on the TCGA-TNBC (training set) and GSE58812 (validation set) datasets. Differential expression analysis, Log-rank test, univariate Cox regression, random forest, and LASSO regression were employed for screen gene sets with diagnostic and prognostic value. A mitochondrial ageing-related risk score (MARS) model was constructed based on LASSO regression. Further analyses were conducted to examine the correlations between MARS and clinicopathological features, copy number variations, drug sensitivity, immune checkpoint expression, and tumor microenvironment. Finally, bioinformatics analysis was conducted to identify PYCR1 expression and potential functions in TNBC.

**Results:**

Based on 52 MAR-DEGs in TNBC, a prognostic signature composed of 4 MAR-DEGs (PYCR1, MAPT, CEBPA, and BCL2A1) was developed. The nomogram incorporating this signature accurately predicted 3-year, 5-year, and 7-year survival rates. Copy number variation (CNV), drug sensitivity, and tumor immune microenvironment analyses revealed that the high-risk group had higher tumor purity and lower immune cell infiltration, as well as lower immunotherapy sensitivity. Immunohistochemical validation of clinical samples revealed that PYCR1 is significantly overexpressed in TNBC tissues. In vitro functional experiments confirmed that knockdown of PYCR1 significantly inhibits the proliferation, migration, and invasion capabilities of TNBC cells.

**Conclusions:**

By integrating multi-omics data and experimental validation, we successfully developed a MARS model with significant prognostic value. We confirmed the high expression of PYCR1 in TNBC and its function in promoting tumor progression, providing new insights for the precision treatment of TNBC.

**Supplementary Information:**

The online version contains supplementary material available at 10.1186/s12935-026-04235-0.

## Introduction

Breast cancer (BC) represents the most prevalent and lethal malignant neoplasm among women, with its incidence rate exhibiting an annual increase [1]. According to the GLOBOCAN 2020 report, there were 2.26 million new cases of breast cancer globally in 2020, surpassing lung cancer to become the most prevalent malignant tumor worldwide [2]. The latest classification method for breast cancer categorizes it into four types: Luminal A, Luminal B, HER2-positive, and TNBC [3]. In some breast cancer patients, the cancer cells are negative for ER, PR, and HER-2, which is referred to as TNBC and accounts for 15% to 20% of all breast cancer cases [4]. Recent clinical consensus indicates that, compared to other types of breast cancer, TNBC has a higher rate of recurrence and metastasis, along with a poorer clinical prognosis [5]. Due to the absence of specific receptors, treatment options for TNBC are very limited [6]. Consequently, early diagnosis of TNBC, precise prediction of patient responses to therapeutic interventions, and the development of novel predictive models are of paramount importance.

Mitochondria are central to cellular metabolism and play a pivotal role in key metabolic activities, including energy production and cellular signal transduction, both of which are essential for organismal health [7]. Mitochondrial dysfunction is a significant contributor to ageing and various age-related diseases, such as cancer, neurodegenerative disorders, cardiovascular diseases, and metabolic syndromes [7]. The function of mitochondria is complex and elusive, particularly in their roles in drug resistance and immune evasion in tumor cells, highlighting the need for ongoing research to elucidate these intricate functions [8]. Currently, relevant studies have confirmed the role of mitochondrial quality-related genes as prognostic and therapeutic biomarkers in lung adenocarcinoma [9]. Meanwhile, TNBC is distinct from other breast cancer subtypes due to its metabolic phenotype, which is predominantly characterized by mitochondrial metabolism [10]. Consequently, inhibiting mitochondrial metabolism presents a promising therapeutic strategy for TNBC. Ageing manifests as a gradual decline in function, occurring heterogeneously across multiple organ systems, ultimately leading to tissue dysfunction [11]. The link between cancer and ageing has been well-documented in numerous epidemiological studies, with cancer incidence increasing exponentially with age after sexual maturity [12]. However, ageing is also characterized as a cellular response marked by stable growth arrest and other phenotypic alterations, which play a role in normal development, maintaining tissue homeostasis, and limiting tumor progression [11]. Therefore, the intricate relationship between ageing and tumorigenesis presents significant challenges in studying the interplay between cancer and ageing.

Mitochondrial dysfunction and ageing are closely intertwined, with each process influencing the other. Mitochondrial dysfunction can accelerate ageing phenotypes, while ageing exacerbates mitochondrial decline through cumulative damage and reduced repair mechanisms. This interplay is particularly relevant in the context of cancer, where both ageing and mitochondrial dysfunction can contribute to tumor initiation, progression, and resistance to therapy [13].

However, the specific interplay between ageing-related mitochondrial dysfunction and TNBC progression remains poorly understood. Given that ageing is a significant risk factor for cancer and that mitochondrial dysfunction is a hallmark of both ageing and TNBC, it is plausible that ageing-related mitochondrial changes may exacerbate the malignant characteristics of TNBC. Understanding this interplay could provide critical insights into the mechanisms driving TNBC progression and identify potential therapeutic targets.

In this study, we aim to explore the relationship between ageing-related mitochondrial dysfunction and TNBC progression. We developed the MARS based on mitochondrial and ageing-related genes, and used it to establish a prognostic model for TNBC patients. Our results provide new insights into the roles of mitochondrial function and ageing in TNBC, highlighting their potential as therapeutic targets and biomarkers for improved diagnosis and prognosis. Furthermore, our study has identified a gene, PYCR1, that regulates mitochondrial function while simultaneously promoting the progression of ageing. This gene is located in tumor cells and can promote the proliferation, migration, and invasion of TNBC tumor cells. Additionally, this gene is associated with a poor prognosis in TNBC and with higher-grade clinical-pathological features. These results suggest that PYCR1 may be an effective target for clinical intervention.

## Methods

### Data Source

The RNA sequencing (RNA-seq) data and clinical information for TNBC samples were obtained from the UCSC Xena database(https://xenabrowser.net/datapages/) [14]. We utilized R software (version 4.4.0) to acquire mutation data using the ‘TCGAbiolinks’ package. To identify TNBC patient samples within the TCGA cohort, we followed the screening methodology outlined in a previous TNBC-related study, excluding samples with incomplete survival data [15]. Ultimately, we identified 191 TNBC samples and 99 adjacent normal tissue samples. We systematically summarized the clinical characteristics of the 191 TNBC patients. The detailed content of the overall clinical information baseline table for TCGA-TNBC is presented in Table S1.

For external validation of the prognostic model, we obtained RNA-seq data and clinical characteristics from a cohort of 107 TNBC patients, sourced from the GSE58812 dataset available in the Gene Expression Omnibus (GEO) (https://www.ncbi.nlm.nih.gov/geo/). In addition, two independent breast cancer chemotherapy cohorts, GSE18728 and GSE20181, were included to evaluate the predictive value of MARS for chemotherapy response.

The METABRIC, GSE76275, and GSE161529 datasets were utilized for the external validation of the PYCR1 gene. The transcriptomic and clinical data for METABRIC were obtained from the METABRIC database (https://www.cbioportal.org/datasets). Following the screening methodology established in previous studies, a total of 355 TNBC samples were ultimately included in the analysis^[24]^. RNA-seq and clinical data for 198 TNBC patients and 265 non-TNBC patients from GSE76275, along with a single-cell RNA sequencing (scRNA-seq) dataset comprising four cases from GSE161529, were retrieved from the GEO database. These datasets were employed to assess PYCR1 expression and conduct prognostic analyses. Detailed sample information for all datasets is provided in Table S2.

Additionally, we utilized the BEST tool (https://rookieutopia.hiplot.com.cn/app_direct/BEST/) to analyze and download multiple breast cancer (BC) expression and survival datasets, which served as external cohorts for PYCR1 expression and prognostic analysis [25].

In processing the GSE161529 dataset, we utilized the R package Seurat to normalize the scRNA-seq data. The normalization was performed using the ‘Normalize Data’ function, with the normalization method set to ‘Log Normalize’. Subsequently, the transformed data were converted into a Seurat object. To ensure the retention of high-quality data, we applied three filtering criteria to the raw scRNA-seq data matrix using the Seurat package. Specifically, we excluded genes detected in fewer than three cells, cells expressing fewer than 200 or more than 7000 genes, and cells exhibiting a high mitochondrial proportion (> 10%). Mitochondria-related genes were obtained from MitoCarta3.0 (https://www.broadinstitute.org/mitocarta/mitocarta30-inventory-mammalian-mitochondrial-proteins-and-pathways) and manually curated based on a previous study [16, 17]. This process resulted in a final list of 2,030 mitochondria-related genes. To identify genes associated with ageing, we utilized the Molecular Signatures Database (MSigDB) (https://www.gsea-msigdb.org/gsea/msigdb/index.jsp) and the Human Ageing Genomic Resources (HAGR) (https://genomics.senescence.info/), ultimately yielding 598 unique ageing-associated genes after deduplication [18, 19]. The mitochondrial and ageing gene sets are detailed in Table S3.

### Identification of MAR-DEGs

We prepared raw transcript count data from 191 TNBC patients and 99 adjacent normal tissue samples within the TCGA-BRCA cohort. Utilizing the ‘DESeq2’ package, we identified differentially expressed genes (DEGs) based on the criteria of adjusted *p-value* (*p-adj*) < 0.05 and |log fold change (logFC)| > 1. Subsequently, we applied the Venn algorithm to determine the intersection of 6223 DEGs, 2030 mitochondria-related genes, and 598 ageing-related genes, resulting in the identification of 52 MAR-DEGs.

### Kaplan-Meier (K-M) survival analysis

We conducted a K-M survival analysis utilizing the ‘survival’ package, setting *p* < 0.05 as the threshold for statistical significance. Additionally, some survival analyses determined the optimal cutoff value using the ‘surv_cutpoint’ function from the ‘survminer’ package in R.

### Identification of DEGs in high- and low-risk score subgroups

In the TCGA-BRCA cohort, 191 TNBC patients were stratified into high- and low-risk groups based on their risk scores. A corresponding baseline table of clinical information was constructed (Table S4). Utilizing the ‘DESeq2’ package, a total of 1,245 DEGs were identified according to the criteria of adjusted *p*-value 0.05, which included 726 upregulated genes and 519 downregulated genes.

### Functional enrichment analysis

We utilized the ‘clusterProfiler’ R package to conduct functional enrichment analysis of DEGs, aiming to elucidate the biological functional differences among various groups. This analysis includes Gene Ontology (GO) assessments, covering Biological Process (BP), Molecular Function (MF), and Cellular Component (CC). Furthermore, we employed Kyoto Encyclopedia of Genes and Genomes (KEGG) pathway analysis to identify pathways significantly affected across different population groups. Additionally, Gene Set Enrichment Analysis (GSEA) was performed to identify predefined gene sets that exhibited significant differences among the groups. Functional terms or pathways were considered statistically significant with a false discovery rate (FDR)-adjusted *p-value* of less than 0.05 and a *p-valu*e of less than 0.05.

### Construction and clinical application of risk signatures

The ‘survival’ package was employed to conduct batch log-rank tests on 52 MAR-DEGs, with a *p-value* threshold of < 0.05 considered statistically significant. This analysis ultimately identified seven genes that exhibit significant survival implications in TNBC. The prognostic significance of these genes in TNBC was evaluated through univariate Cox regression analysis, with the results illustrated in a forest plot. A *p-value* of < 0.05 was again regarded as statistically significant, leading to the identification of four genes with notable prognostic relevance. Subsequently, we utilized the ‘randomForestSRC’ package to perform a random forest analysis on these four genes, thereby validating their diagnostic significance in TNBC.

The ‘glmnet’ R package was utilized to conduct the Least Absolute Shrinkage and Selection Operator (LASSO) model analysis. Weighted LASSO coefficients, derived from individual gene expression levels, were employed to compute the MARS. We implemented 10-fold cross-validation to optimize the model and mitigate overfitting. The prognostic risk score in this model was calculated as follows:

$$\begin{aligned}&\:MARS\:=\:Expression\:of\:Gene\:1\:*\:Coef\:1\: \\& \quad+\:Expression\:of\:Gene\:2\:*\:Coef\:2\: \\& \quad+\:Expression\:of\:Gene\:3\:*\:Coef\:3\:\ \\& \quad+\:...\:+\:Expression\:of\:Gene\:n\:*\:Coef\:n\left[L1\right]\:\end{aligned}$$

In this study, TNBC samples were stratified into high-risk and low-risk subgroups based on the median risk score for subsequent analysis. The Receiver Operating Characteristic (ROC) curve was generated using the ‘survivalROC’ package in R. Survival curves for the high-risk and low-risk groups were constructed using the ‘survival’ and ‘survminer’ packages in R, with a *p-value* < of less than deemed considered significant for survival differences between subgroups.

### Construction and evaluation of the nomogram

Considering that clinicopathological variables significantly influence survival outcomes, we constructed a nomogram utilizing TCGA clinicopathological parameters alongside MARS to enhance the predictive accuracy of MARS. Clinicians can leverage the points derived from the nomogram to estimate the patients’ survival probabilities. Calibration curves and ROC curves were generated, and the area under the curve (AUC) was calculated to assess the predictive capability of the nomogram.

### Exploration of gene mutation patterns

We utilized the ‘maftools’ package to explore the somatic mutation data of TNBC patients. The ‘ggplot2’ package was employed to create Oncoplots, which visualize the somatic mutation landscape of the top 20 mutated genes within the cohort, stratified by risk score levels. To evaluate the Tumor Mutational Burden (TMB) of the patients, we processed the Mutation Annotation Format (MAF) files using the maf() function from the TMB package.

### Drug sensitivity analysis

The drug sensitivity training data were downloaded from the Genomics of Drug Sensitivity in Cancer (https://www.cancerrxgene.org) [20]. Subsequently, these data were validated using TCGA data to identify commonly used anti-tumor drugs for our analysis. The results were effectively presented through grouped comparison plots utilizing the ‘oncoPredict’ and ‘ggpubr’ packages. The Wilcoxon test was employed to ascertain the statistical differences between various groups, with a *p-value* < 0.05 considered statistically significant.

### Immune checkpoint and the tumor microenvironment analysis

Through a comprehensive literature review, we identified 13 commonly utilized immune checkpoints. We applied the ESTIMATE algorithm and the Cell-type Identification by Estimating Relative Subsets of RNA Transcripts (CIBERSORT) method to analyze the tumor microenvironment. The ESTIMATE algorithm utilizes the ‘estimate’ software package to assess the proportions of immune cells, stromal cells, and tumor cells in 191 TNBC samples obtained from the TCGA database. Using 1,000 permutations of the LM22 signature, the CIBERSORT method was employed to calculate the abundance of 22 major immune cell subtypes.

### Prediction of immunotherapeutic efficacy

Each sample was scored using the TIDE algorithm (http://tide.dfci.harvard.edu/) to assess the predicted efficacy of immunotherapy between the two risk groups, with samples having a TIDE score less than 0 defined as responders.

### Pan-cancer analysis

We utilized the ‘TCGAplot’ package to conduct a comprehensive pan-cancer analysis and visualization of multi-omics data related to PYCR1 within TCGA. The source code and pre-built package are available on GitHub (https://github.com/tjhwangxiong/TCGAplot) [21].

### Clinical specimens and immunohistochemistry (IHC)

We collected paraffin-embedded tissues from 15 breast surgery patients at Zhongda Hospital, Southeast University (Nanjing, China) between September 2024 and December 2024 for IHC analysis. The PYCR1 antibody was obtained from ProteinTech, and the tissue sections underwent IHC staining performed by the Department of Pathology at Zhongda Hospital Southeast University. The expression levels of the PYCR1 protein were evaluated based on both staining intensity and quantity. This study was conducted with the approval of the Clinical Research Ethics Committee of Zhongda Hospital, Southeast University (approval number: 2023ZDSYLL056-P01).

### Identification of PYCR1-associated genes

The TCGA-BRCA cohort, which includes191 patients with TNBC, was stratified into high and low expression groups based on the median expression level of PYCR1. Subsequently, a corresponding baseline clinical information table was constructed (Table S5). Utilizing the ‘DESeq2’ package, DEGs were identified based on the criteria of adjusted *p-value* < 0.05 and |logFC| > 0.5, resulting in the identification of 237 upregulated genes and 625 downregulated genes.

### WGCNA network construction and module identification

To investigate the gene modules highly associated with PYCR1 gene expression, we employed the Weighted Gene Co-expression Network Analysis (WGCNA) technique to construct a gene co-expression network, incorporating 6,223 DEGs. In our analytical workflow, we initially identified outliers in the samples by constructing a clustering dendrogram. Subsequently, based on the analysis of network topology, we determined the optimal soft-thresholding power for calculating the adjacency between genes. These adjacency data were transformed into a topological overlap matrix (TOM) to quantify the strength of associations between genes. Using the TOM, we calculated gene connectivity and constructed a hierarchical clustering tree for the genes. Through the dynamic tree cut method, we identified gene modules with similar expression patterns and performed a merging process on these modules. Within these modules, we specifically focused on the one exhibiting the strongest correlation with PYCR1 expression, from which we screened for key genes to serve as the focal point for subsequent analysis.

### Cell Culture and siRNA transfection

The human breast cancer cell line MDA-MB-231 was obtained from the Cell Bank of the Chinese Academy of Sciences (Shanghai, China). These cells were cultured in DMEM medium (Thermo Fisher, USA), supplemented with 10% fetal bovine serum (FBS) (Royacel RYS-KF22, China) and 1% penicillin/streptomycin (Beyotime, Shanghai, China), and were maintained in a 37°C incubator with 5% CO2. The small interfering RNA (siRNA) targeting PYCR1 was designed and synthesized by GenePharma (Shanghai, China). The siRNA sequences are as follows: si-NC: 5′-UUCUCCGAACGUGUCACGUTT-3′ (sense) and 5′-ACGUGACACGUUCGGAGAATT-3′ (antisense); si-PYCR1: 5’-GUGUGAAGAUGGGACUUCCTT-3’ (sense) and 5’-GGAAGUCCCAUCUUCACACTT-3’ (antisense).

### Quantitative real-time PCR (qRT-PCR)

Total RNA was extracted from cultured cells using the RNAeasy™ Animal RNA Isolation Kit (Beyotime, Shanghai, China) following the manufacturer’s instructions with spin columns. Real-time quantitative reverse transcription polymerase chain reaction (RT-qPCR) was conducted using the Taq Pro Universal SYBR qPCR Master Mix obtained from BestEnzymes (Lianyungang, China). The primers for PYCR1 and the internal control GAPDH were sourced from GENERAL BIOL (Chuzhou, China). The sequences of the primers are as follows: PYCR1: F: 5’-TGGCTGCCCACAAGATAATGG-3’, R: 5’-CGTGACGGCATCAATCAGGT-3’; GAPDH: F: 5’-ACCCAGAAGACTGTGGATGG-3’, R: 5’-TCAGCTCAGGGATGACCTTG-3’.

### Western blotting

Total protein was isolated with RIPA lysis buffer (KeyGene Biotech, KGB5203, 1:1000) and quantified by bicinchoninic acid assay ( BCA, KeyGene Biotech, KGB2101). Samples were separated by SDS-PAGE, transferred to PVDF membranes (Merck Millipore, SVLP04700), blocked for 1-hour h in 5% skim milk, incubated overnight with primary antibodies, and probed with horseradish peroxidase (HRP) -conjugated secondary antibodies.

### CCK8 assay

Seed 200 µL of cell suspension (2 × 10^5 cells/well) into a 96-well plate and incubate at 37 °C in a humidified atmosphere with 5% CO2. After culturing the cells for 24, 48, 72, 96, and 120 h, add 20 µL of CCK8 reagent (Beyotime, Shanghai, China) to the culture medium and incubate again at 37 °C in a humidified atmosphere containing 5% CO2 for 2 h. Subsequently, measure the absorbance at 450 nm using a microplate reader (Bio-Tek, USA).

### Wound healing assay

Cells were seeded in 6-well plates and transfected with si-/nc-PYCR1. After reaching confluence, a 200 µL pipette tip was employed to create a scratch in the monolayer of cells. The scratched areas were subsequently washed with PBS. Following this, the TNBC cells were cultured in serum-free medium. Wound healing was observed at 0, 12, 24, and 48 h under a microscope, and photographs were taken at each time point.

### Transwell migration assay

The Transwell migration assay was conducted using 8.0 μm Transwell chambers (LABSELECT, Beijing, China). The lower chamber was filled with 600 µL of medium supplemented with 10% FBS, while the upper chamber contained 200 µL of cell suspension at a density of 5 × 10^4 cells/ml. After incubating at 37 °C with 5% CO2 for 12 h, the cells in the upper chamber were removed and fixed with 600 µL of 4% paraformaldehyde solution (Servicebio, China) for 30 min. The fixative was then discarded, and the cells were stained with 600 µL of 0.1% crystal violet solution (Keygen, China) for an additional 30 min, followed by three washes with phosphate-buffered saline (PBS). After allowing the cells to air-dry, photographs of the migrated cells were captured under a microscope.

### Plate cloning experiment

Add 2 mL of cell suspension (1 × 10³ cells/well) to a 6-well plate and culture in a 37 °C incubator with 5% CO₂. Change the medium every three days. Depending on the growth conditions, discard the medium after 7 to 14 days and add 1 mL of 4% paraformaldehyde solution to fix the cells for 30 min. After discarding the fixative, apply 1 mL of 0.1% crystal violet solution to stain the cells for 30 min, followed by three washes with PBS. Allow the cells to air dry naturally before observation.

### Animal experiments

Six-week-old female BALB/c nude mice (GemPharmatech, Nanjing) were housed under SPF conditions and used to establish xenografts. A 50 µL PBS–Matrigel (1:1) suspension containing 5 × 10⁶ MDA-MB-231 cells pretreated with si-NC or si-PYCR1 was injected subcutaneously into the axilla. Tumor dimensions were recorded with electronic calipers every third day and volume computed as 0.5 × length × width². On day 24, animals were euthanized, and xenograft tumors were promptly excised, weighed, and processed for downstream analyses. All procedures were approved by the Animal Ethics Committee of Southeast University (approval No.SEU-IACUC-20251209001).

### Statistical analysis

All experimental data are presented as the mean ± standard deviation (SD) of three or more biological replicates, with each sample run in three technical replicates. Statistical analyses were conducted using R (version 4.4.0).

For comparisons between two groups, we used Student’s t-test for normally distributed data (e.g., in vitro assays) and the Wilcoxon rank-sum test for non-normally distributed omics data. A raw *p-value* < 0.05 was considered statistically significant for these individual comparisons, denoted as follows: *p < 0.05, ** *p* < 0.01, *** *p* < 0.001, **** *p* < 0.0001.

Multiple testing correction was rigorously applied to control the false discovery rate (FDR) in high-dimensional analyses. The Benjamini–Hochberg method was used throughout, with significance defined as an FDR-adjusted *p-value* (*p-adj*) < 0.05 for the following: Differential expression analysis, functional enrichment analysis (GO, KEGG, GSEA), batch survival screening (e.g., log-rank tests across multiple genes), and group comparisons involving multiple variables (e.g., immune cell fractions, drug sensitivity) were performed using Wilcoxon tests, followed by FDR correction across all compared items.

## Results

### Identification of MAR-DEGs in TNBC

**Fig. 1:**
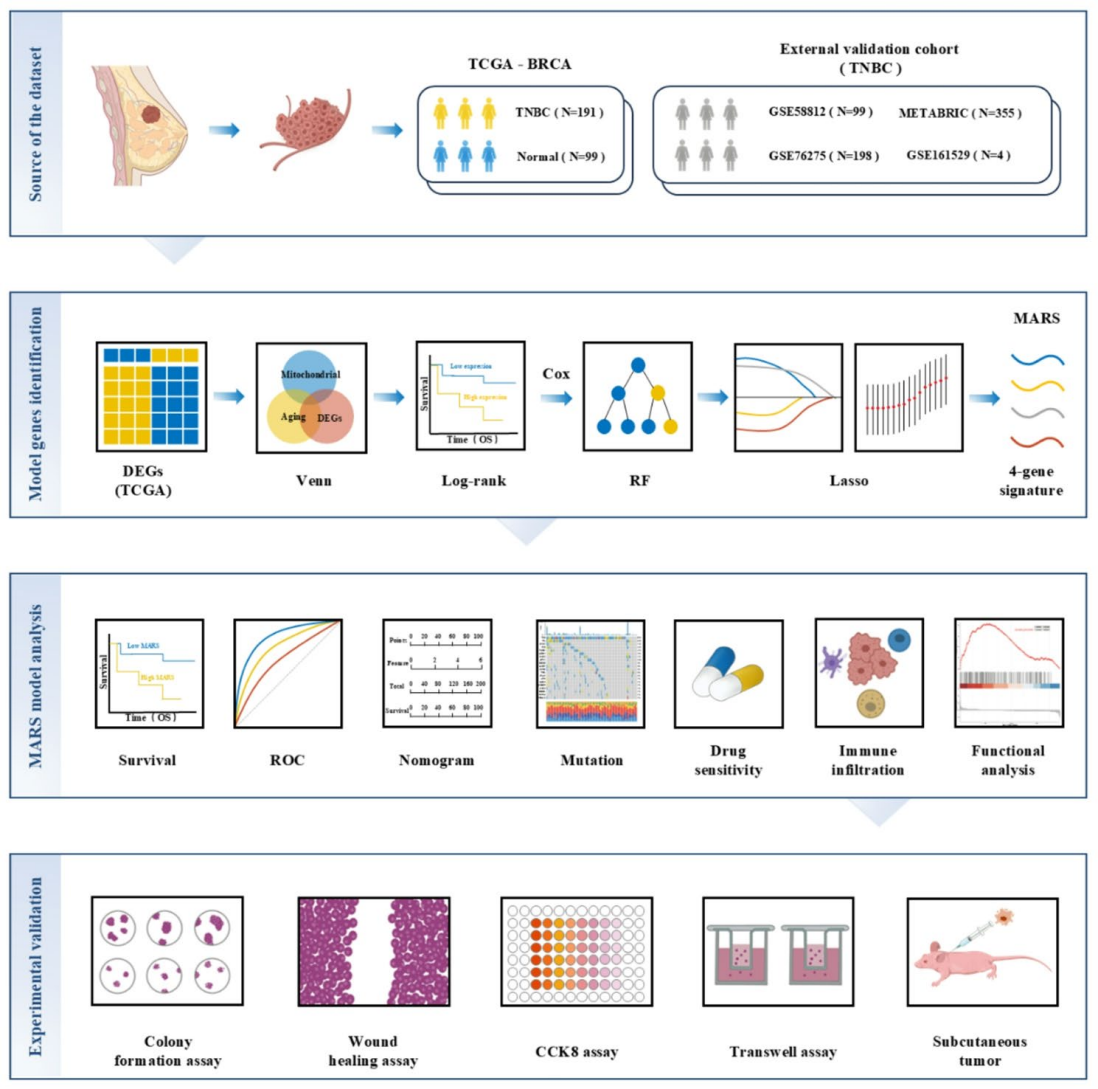
The technical roadmap of this study

**Fig. 2:**
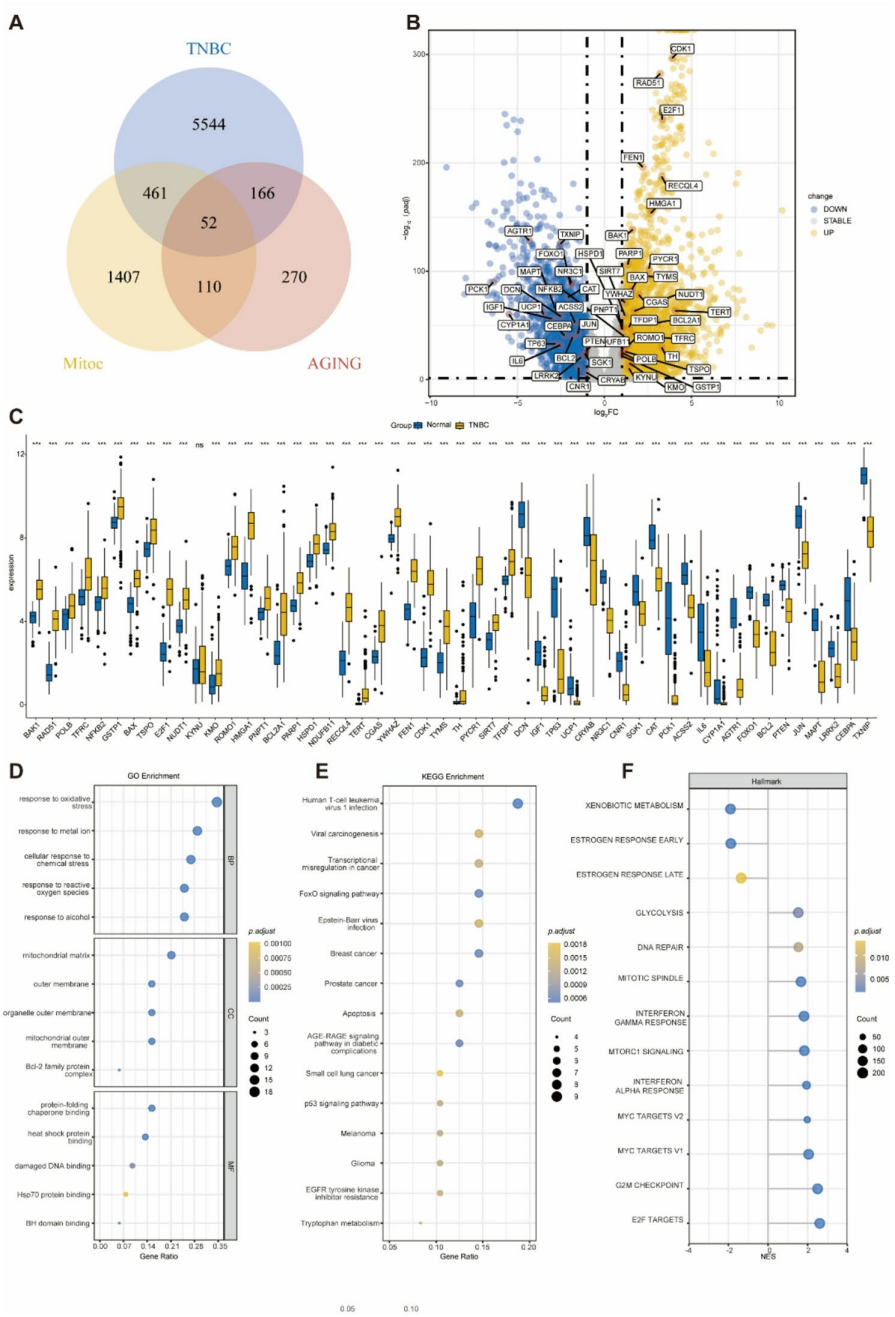
Identification of MAR-DEGs in TNBC. **A**: A Venn diagram showing the overlap between DEGs in TNBC and genes in the mitochondrial and senescence gene sets. **B**: A volcano plot of DEG expression, with labels highlighting the 52 MAR-DEGs. **C**: Expression levels of the 52 MAR-DEGs in TNBC tissues and adjacent normal tissues. **D**: Bubble chart of GO functional enrichment analysis for the 52 MAR-DEGs. **E**: Bubble chart of KEGG functional enrichment analysis for the 52 MAR-DEGs. **F**: Bar chart of Hallmark enrichment analysis results after GSEA filtering, sorted by NES values. The size of the dots is proportional to the number of overlapping genes, and the color is encoded based on the *p-value*

The research process is illustrated in Fig. 1. In this study, we screened 191 TNBC samples alongside 99 adjacent normal tissue samples from the TCGA database, resulting in the identification of a total of 6,223 DEGs. Among these, 3,817 genes were found to be upregulated, while 2,406 genes were downregulated in TNBC (Table S6). Through Venn diagram overlap analysis, we identified 52 MAR-DEGs specific to TNBC (Fig. 2A). These 52 MAR-DEGs are annotated in the volcano plot (Fig. 2B), and their expression patterns are visualized in the TCGA database (Fig. 2C).

To understand the biological functions of MAR-DEGs, we conducted a functional enrichment analysis on these 52 MAR-DEGs. The GO enrichment analysis revealed that the MAR-DEGs are involved in various biological processes, including responses to oxidative stress, metal ions, chemical stress, reactive oxygen species, and alcohol. Additionally, they are associated with mitochondrial components, including the mitochondrial matrix, outer membrane, and outer mitochondrial membrane, as well as interactions with Bcl-2 family protein complexes, protein folding chaperone binding, heat shock protein binding, damaged DNA binding, Hsp70 protein binding, and BH domain binding (Fig. 2D). Furthermore, the KEGG analysis indicated that these genes are linked to several pathways, including human T-cell leukemia virus 1 infection, viral carcinogenesis, transcriptional misregulation in cancer, the FoxO signaling pathway, Epstein-Barr virus (EBV) infection, breast cancer, prostate cancer, apoptosis, the AGE-RAGE signaling pathway in diabetic complications, small cell lung cancer, the p53 signaling pathway, melanoma, glioma, EGFR tyrosine kinase inhibitor resistance, and tryptophan metabolism (Fig. 2E).

GSEA using Hallmark gene sets from the MSigDB database revealed that, compared to adjacent normal tissues, pathways such as xenobiotic metabolism, early estrogen response, late estrogen response, and hypoxia were downregulated in TNBC. Conversely, pathways including the glycolysis, DNA repair, mitotic spindle, interferon gamma response, mTORC1 signaling, interferon alpha response, MYC targets V2, MYC targets V1, G2M checkpoint, and E2F targets were found to be upregulated (Fig. 2F). These findings imply that mitochondrial function and ageing may significantly contribute to the pathogenesis of TNBC, providing new insights and potential therapeutic targets for future research.

### Construction of Mitochondrial Ageing-Related Prognostic Signatures

**Fig. 3:**
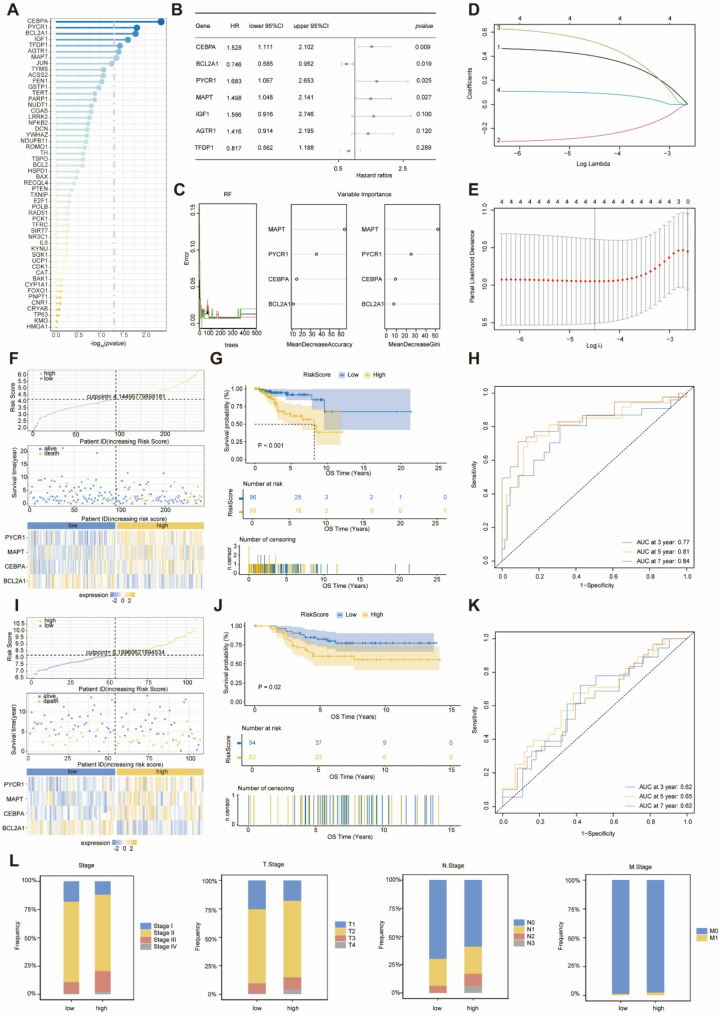
Construction of the mitochondrial senescence-related prognostic signature and validation of its prognostic value. **A**: Bar plot of log-rank test results for the 52 MAR-DEGs, with the x-axis representing -log_10_(*p-value*). The size of the dots is proportional to -log_10_(*p-value*), and the gray dashed line indicates -log_10_(0.05). **B**: Forest plot of univariate Cox regression analysis results, showing that four MAR-DEGs are associated with poor prognosis in TNBC. **C**: Diagnostic value of the four MAR-DEGs evaluated by the RF algorithm and ranked by importance. **D**,** E**: LASSO coefficient profiles and selection of prognostic MAR-DEGs by minimum criteria cross-validation. **F**,** I**: Risk score nomograms depicting risk scores, survival time distribution, and expression of PYCR1, MAPT, and CEBPA in the training and validation cohorts. **G**,** J**: Kaplan-Meier curves for OS of TNBC patients in the training and validation cohorts, stratified by median MARS. **H**,** K**: Time-dependent ROC analysis for predicting 3-year, 5-year, and 7-year OS in TNBC patients in the training and validation cohorts. Panels **F–H** correspond to the training cohort, and panels** I–K** to the validation cohort. **L**: Stacked bar chart showing the percentage distribution of clinical stage and TNM staging (T, N, M) in patients with low and high MARS levels

The 52 MAR-DEGs were subjected to K-M analyses of overall survival (OS) (log-rank test), and seven prognostic MAR-DEGs (CEBPA, BCL2A1, PYCR1, MAPT, IGF1, AGTR1, TFDP1) were screened with log-rank *p-adj* values < 0.05(Fig. 3A). To identify potential prognostic risk factors among these seven prognostic MAR-DEGs, we further conducted the univariate Cox regression analysis. CEBPA, BCL2A1, PYCR1, and MAPT were ultimately considered potential risk factors for poor OS in TNBC (Fig. 3B). We randomly selected 70% of the TNBC samples as the training set, while the remaining 30% served as the validation set. The relationships among error rate, the number of classification trees, and the four genes were assessed using RF with feature selection, with the genes ranked in descending order of relative correlation. The RF diagnostic model’s accuracy for these four genes reached 96.59% (Fig. 3C). Subsequently, a Lasso logistic regression model was developed to predict the prognosis of TNBC patients. All four MAR-DEGs with prognostic and diagnostic value were included to generate the optimal risk score model (Figs. 3D, E). The calculation formula for the mitochondrial ageing-related risk score (designated as MARS) is as follows:   

$$\begin{aligned}&\:MARS\:=\:expression\:level\:of\:CEBPA\:*\:\left(0.4631\right)\:\\&\quad+\:expression\:level\:of\:BCL2A1\:*\:(-0.3094)\: \\& \quad+\:expression\:level\:of\:PYCR1\:*\:\left(0.6262\right)\: \\& \quad+\:expression\:level\:of\:MAPT\:*\:\left(0.1083\right)\end{aligned}$$

### Internal Training and External Validation of the MARS Prediction Model

Based on the median MARS calculated using this formula, we stratified the 191 TNBC patients from the TCGA cohort into low- and high-risk groups, which served as the training dataset. Concomitantly, 107 triple-negative breast cancer samples from the GSE58812 cohort were dichotomized into low- and high-risk groups according to their median MARS value and designated as the validation set. The ternary plot of the risk score illustrates the distribution of the MARS model across both the training and validation cohorts, indicating that the mortality rate among TNBC patients increases with higher MARS levels. Notably, elevated expressions of PYCR1, MAPT, and CEBPA were observed in the high MARS group (Fig. 3F, I). In both cohorts, TNBC patients exhibiting high MARS levels demonstrated a significantly shorter predicted OS compared to those with low MARS levels, with a *p-value* of < 0.001 in the training set and 0.02 in the validation set (Fig. 3G, J). Furthermore, the time-dependent ROC curves illustrated the robust stability of the MARS model in the training cohort (3-year AUC: 0.77; 5-year AUC: 0.81; 7-year AUC: 0.84; Fig. 3H) and the validation cohort (3-year AUC: 0.62; 5-year AUC: 0.65; 7-year AUC: 0.62; Fig. 3K). Additionally, while no statistically significant differences were observed between MARS and the various clinical stages (I-IV), T stages (T1-T4), N stages (N0-N3), and M stages (M0-M1) of TNBC, patients classified in the low-risk score group were more frequently found in the earlier stages of the TNM staging system. This trend suggests a correlation between the scoring system and the extent of tumor progression **(**Fig. 3L**)**.

### Establishment and Evaluation of a Hybrid Prognostic Nomogram Model Integrated With

**Fig. 4:**
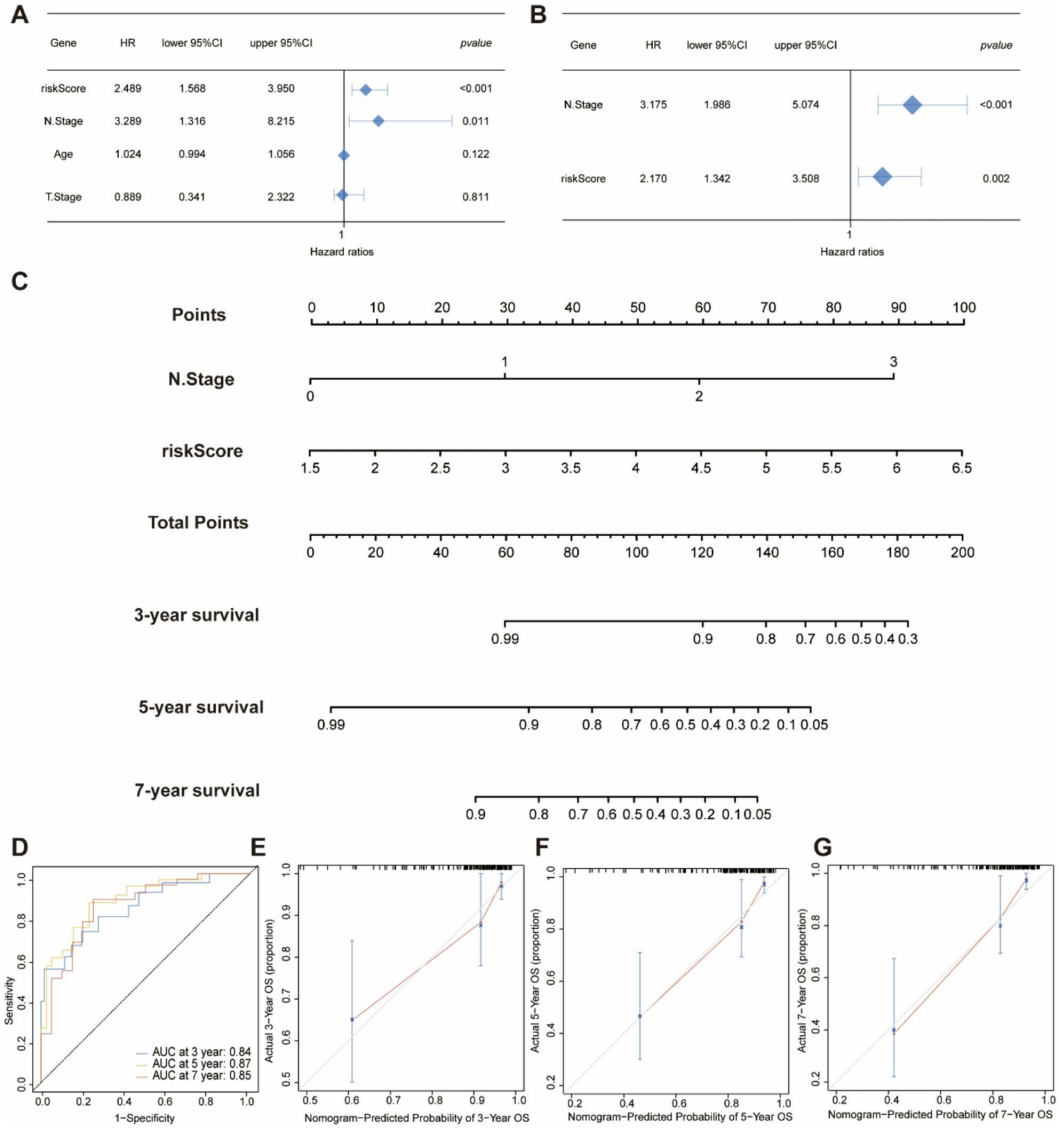
Construction of the nomogram for predicting OS in TNBC patients. **A**: Forest plot of univariate Cox regression analysis results showing MARS and clinical features in TNBC patients. **B**: Forest plot of multivariate Cox regression analysis results showing MARS and clinical features in TNBC patients. **C**: Nomogram for predicting 3-year, 5-year, and 7-year OS in TNBC patients based on MARS and T stage. **D**: Time-dependent ROC analysis for predicting 3-year, 5-year, and 7-year OS in TNBC patients using the nomogram. **E**,** F**,** G**: Calibration curves for predicting 3-year, 5-year, and 7-year OS in TNBC patients based on the nomogram

In addition, we established a nomogram model integrated with independent risk factors, specifically T.Stage and MARS. To assess whether the MARS model serves as an independent prognostic factor, we performed a Cox analysis combining MARS with clinical variables, including clinical T stage (cT), clinical N (cN) stage, and age. Univariate Cox regression analysis indicated that MARS is a significant high-risk factor for patients with TNBC [HR = 2.489, 95% CI (1.568, 3.950), *p* < 0.001, Fig. 4A]. Furthermore, multivariate Cox analysis reinforced that MARS may act as an independent risk factor for OS in TNBC [HR = 2.170, 95% CI (1.342, 3.507), *p* < 0.001, Fig. 4B]. Subsequently, we developed a nomogram that integrates N stage and the MARS model to assist clinicians in predicting the survival of TNBC patients (Fig. 4C). The predictive accuracy of our nomogram exceeded that of the MARS alone, demonstrating 3-year, 5-year, and 7-year AUCs of 0.84, 0.87, and 0.85, respectively. This indicates that the inclusion of clinical characteristics enhances the predictive efficacy of the MARS model (Fig. 4D). The calibration plots for 3-, 5-, and 7-year outcomes demonstrated the stable performance of the nomogram (Fig. 4E, F, G).

### Analysis of Mutation Status and Drug Sensitivity in Different Risk Subgroups

**Fig. 5:**
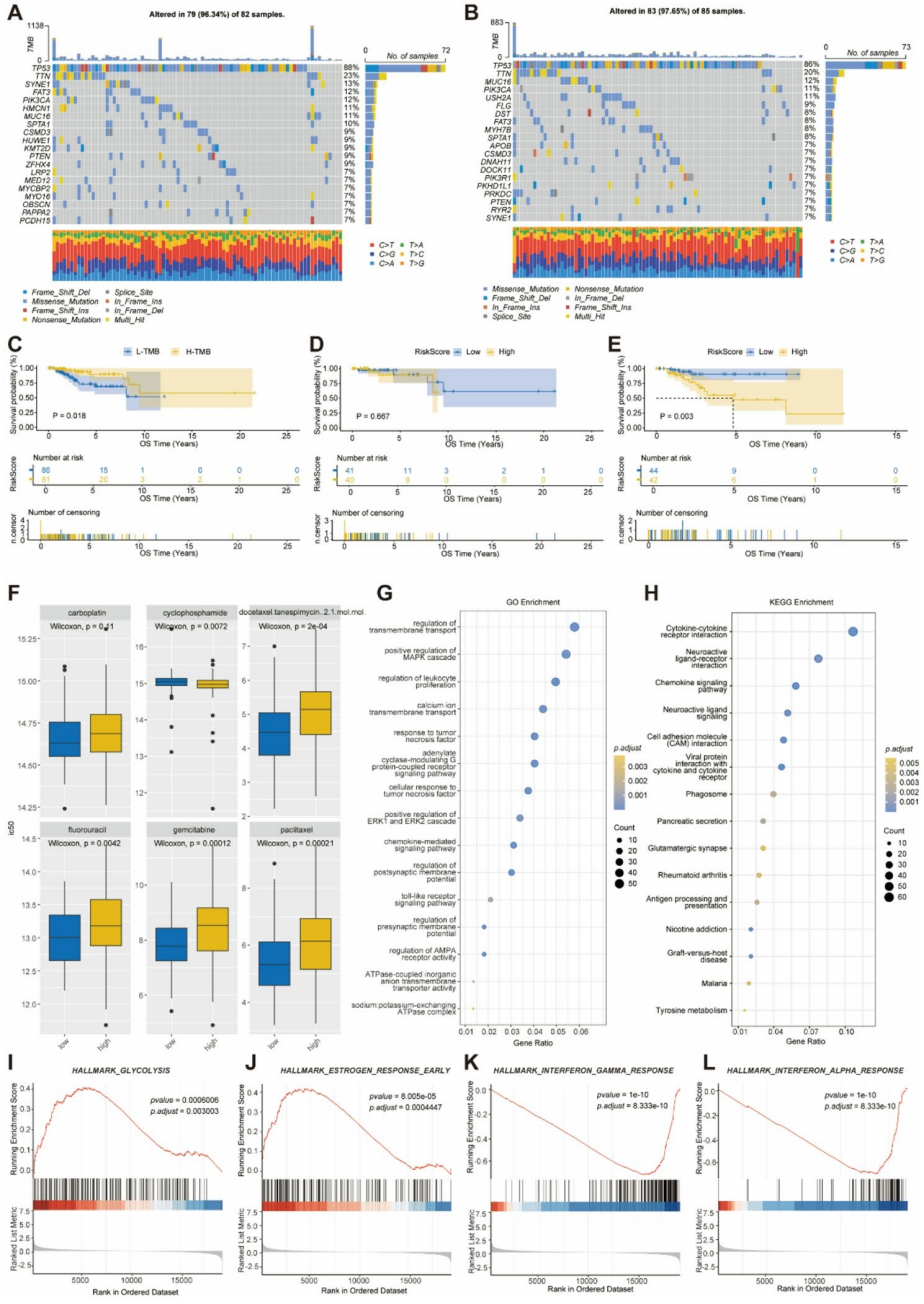
Mutation landscape, drug sensitivity, and mechanism analysis of MARS in the TCGA-TNBC training cohort. **A**: Top 20 genes with the highest mutation frequency in the high-risk group. **B**: Top 20 genes with the highest mutation frequency in the low-risk group. **C**: Kaplan-Meier curves for OS of patients with high and low tumor mutation burden (TMB). **D**: Kaplan-Meier curves for OS in the high TMB subgroup, comparing high-risk and low-risk groups. **E**: Kaplan-Meier curves for OS in the low TMB subgroup, comparing high-risk and low-risk groups. **F**: IC50 values of six common clinical chemotherapeutic drugs in high- and low-risk subgroups. **G**: Bubble chart of GO functional enrichment analysis for 1245 DEGs between high- and low-risk subgroups. **H**: Bubble chart of KEGG functional enrichment analysis for 1245 DEGs between high- and low-risk subgroups. **I**: GSEA functional enrichment analysis of the glycolysis pathway. **J**: GSEA functional enrichment analysis of the estrogen response early pathway. **K**: GSEA functional enrichment analysis of the interferon gamma response pathway. **L**: GSEA functional enrichment analysis of the interferon alpha response pathway

The development and progression of tumors are closely associated with genetic mutations. Therefore, we mapped the tumor mutation landscape between the high-risk and the low-risk score TNBC subgroups, presenting the top 20 high-frequency mutated genes in each group. In the high-risk score subgroup, 96.34% of the samples exhibited somatic mutations, with TP53, a classical tumor suppressor gene, being the most frequently mutated, appearing in 88% of the samples. This was followed by TTN, SYNE1, FAT3, and PIK3CA (Fig. 5A). In the low-risk score subgroup, 97.65% exhibited significant genetic alterations, with TP53 again showing the highest mutation frequency, present in 86% of the samples, followed by TTN, MUC16, PIK3CA, and USH2A (Fig. 5B).

TMB is a valuable and clinically accessible predictive biomarker for cancer prognoses and immunotherapy responses. Our analysis revealed that the high TMB subgroup exhibited significantly better OS compared to the low TMB subgroup (p=0.018, Figure 5C). This finding may be attributed to the likelihood that patients with high TMB tumors possess a pre-activated immune system, which can be further stimulated by immune checkpoint inhibitors (ICIs). Although there was no statistically significant difference in OS between the high-risk and low-risk groups within the high TMB subgroup (p=0.667, Figure 5D), it was noted that the low-risk group demonstrated better OS than the high-risk group in the low TMB subgroup (p=0.003, Figure 5E). In conclusion, the combination of MARS and TMB may serve as a valuable biomarker set for predicting the prognosis of patients with TNBC.

To further explore the differences in drug sensitivity between high-risk and low-risk score subgroups, we calculated the association between the MARS score and the half-maximal inhibitory concentration (IC50) of several anticancer drugs using the ‘oncoPredict’ package in R software. Fluorouracil, paclitaxel, carboplatin, gemcitabine, docetaxel, and temsirolimus are six common chemotherapeutic agents used in the treatment of TNBC (Fig. 5F). Our findings indicate that, with the exception of carboplatin and cyclophosphamide, the high-risk score subgroup exhibited significantly lower sensitivity to the remaining four drugs compared to the low-risk score subgroup. In summary, our data suggest that MARS may serve as a predictive biomarker to guide clinical drug selection in the treatment of TNBC.

To investigate the potential mechanisms of MARS in TNBC, we stratified 191 TNBC patients from the TCGA-BRCA database into high-risk and low-risk groups based on their risk scores. Utilizing the “DESeq2” package, we identified 1,245 DEGs (Table S6). Further GO enrichment analysis (Fig. 5G) revealed that MARS may be associated with various biological processes, including the positive regulation of the MAPK cascade, response to tumor necrosis factor, regulation of AMPA receptor activity, and ATPase-coupled inorganic anion transmembrane transporter activity. Additionally, the results of the KEGG enrichment analysis (Fig. 5H) suggest that MARS may be involved in several signaling pathways, including the Cytokine-cytokine receptor interaction, Neuroactive ligand-receptor interaction, Chemokine signaling pathway, and Cell adhesion molecules (CAMs).The GSEA results indicate that in the high-risk subgroup, glycolysis and the early estrogen response pathway are significantly enriched (Fig. 5I, J). In contrast, the low-risk subgroup exhibits significant enrichment in the interferon γ response and interferon α response pathways (Fig. 5K, L). These findings demonstrate substantial differences in gene expression patterns between the high-risk and low-risk subgroups, which may be closely related to disease progression and occurrence, thereby providing potential biological targets for future research.

### Immune microenvironment landscape

**Fig. 6:**
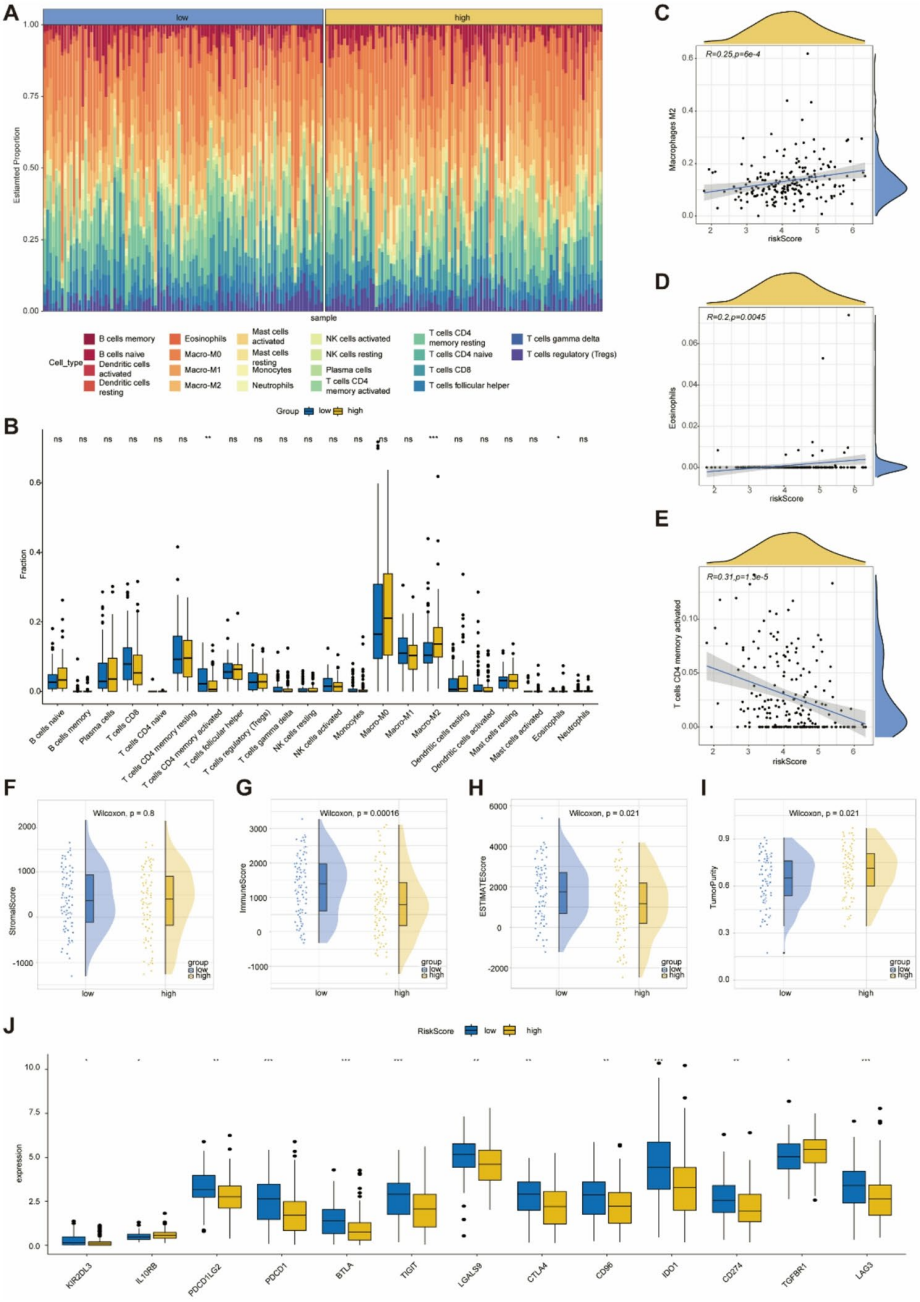
Immune cell landscape between high- and low-risk subgroups. **A**: Bar plot showing the proportions of 22 immune cell types calculated by CIBERSORT in high- and low-risk subgroups, with each bar representing a patient and each color representing an immune cell type. **B**: Expression levels of immune cells between high- and low-risk subgroups. **C**: Macrophages M2 are positively correlated with risk score. **D**: Eosinophils are positively correlated with risk score. **E**: T cells CD4 memory activated are negatively correlated with risk score. **F**: Stromal score. **G**: Immune score. **H**: ESTIMATE score. **I**: Tumor purity. **J**: Bar plot comparing the expression levels of immune checkpoints between the high-risk and low-risk groups. * *p* < 0.05, ** *p* < 0.01, *** *p* < 0.001

Considering the close relationship between tumor progression and the immune microenvironment, we analyzed the differences in the immune landscape between high-risk and low-risk score subgroups in the TCGA-TNBC training cohort using two methods. We employed the CIBERSORT algorithm to predict the relative abundance of 22 tumor-infiltrating immune cells in each sample of the TCGA-TNBC training cohort (Fig. 6A). Our analysis revealed statistically significant differences in the abundance of T cells (CD4 memory activated), macrophages (M2) and eosinophils between the high-risk and low-risk score subgroups (all *p* < 0.05, Fig. 6B). Further correlation analysis indicated that macrophages (M2) and eosinophils exhibited a positive correlation with the risk score (Fig. 6C, D), while T cells (CD4 memory activated) were negatively correlated with the risk score (Fig. 6E). Subsequently, differential analysis was conducted based on the Stromal score (Fig. 6F), Immune score (Fig. 6G), ESTIMATE score (Fig. 6H), and Tumor purity (Fig. 6I) as calculated by the ESTIMATE algorithm. While no statistically significant difference was observed in the stromal score, the immune score and ESTIMATE score were notably lower in the high-risk score subgroup. In contrast, this subgroup exhibited higher tumor purity. To further investigate the expression levels of various immune checkpoints within the training set, we analyzed the correlation between high and low-risk score subgroups and immune checkpoints. The differential expression results of these components are illustrated in Fig. 6J, where immune checkpoint genes such as PDCD1LG2, PDCD1, LAG3, TIGIT, BTLA, LGALS9, CTLA4, and CD96 were significantly upregulated in the low-risk score subgroup (*p < 0.05*). These findings comprehensively highlight the distinct immune characteristics between high-risk and low-risk TNBC, suggesting that the population with low risk may exhibit greater immune cell infiltration and could potentially benefit from ICIs.

### The therapeutic benefit of the MARS value

After that, we predicted the immunotherapy efficacy with the TIDE algorithm based on TCGA and GSE58812. Our results showed that patients with TIDE > 0 (Non-Responders) had significantly higher MARS levels. Moreover, in high-risk patients with MARS, there is decreased T-cell infiltration, exacerbation of immune rejection or impairment of function, and immune-evasion characteristics, such as low IFNG and low Merck18 (Figures S1A-H). We further analyzed Disease-Free Interval (DFI) and Metastasis-Free Survival (MFS) in the high- and low-MARS groups in that study. The high MARS group of the TCGA cohort had a lower DFI (Figure S1I). Likewise, within the GSE58812 cohort, elevated MARS was significantly associated with poorer MFS, indicating an increased risk of treatment resistance and early failure (Figure S1J). Eventually, we found that MARS was significantly reduced after adjuvant chemotherapy in two independent adjuvant chemotherapy cohorts (GSE18728, GSE20181). The paired P-values were < 0.05 in both cohorts, suggesting that MARS might predict chemotherapy efficacy (Figure S1K-N).

### High Expression of PYCR1 Predicts Poor Prognosis in TNBC Patients

**Fig. 7:**
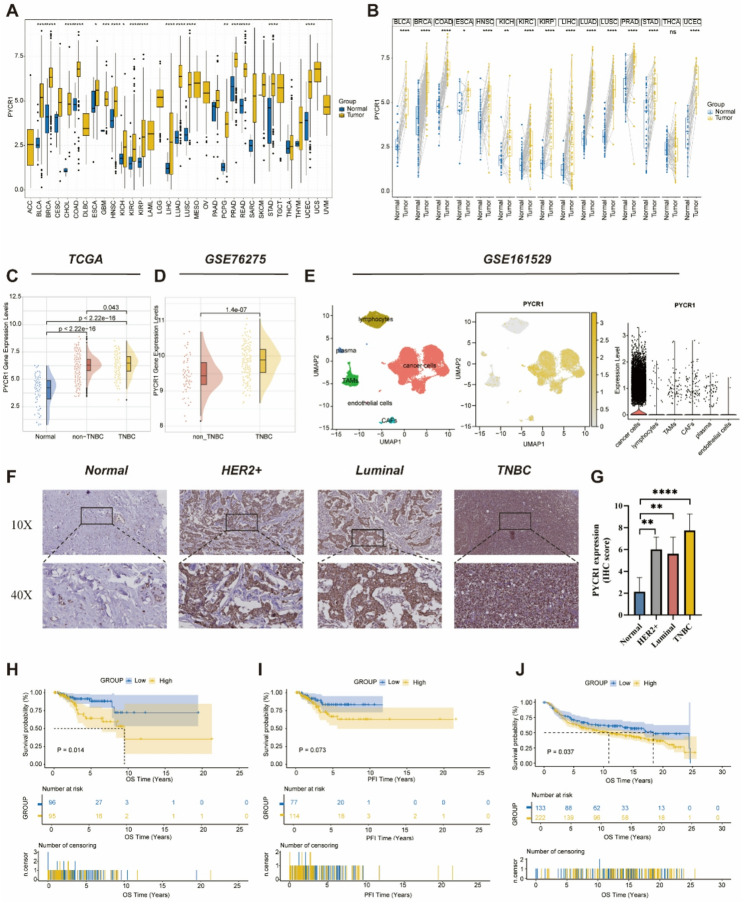
Overexpression of PYCR1 is associated with poor prognosis in TNBC. **A**: Expression of PYCR1 in tumor and adjacent normal tissues across pan-cancer. **B**: Expression of PYCR1 in tumor and adjacent normal tissues in paired samples across pan-cancer. **C**: In the TCGA-BRCA cohort, expression levels of PYCR1 in adjacent normal tissues, non-TNBC tumor tissues, and TNBC tumor tissues. **D**: In the GSE76275 cohort, expression levels of PYCR1 were compared between non-TNBC tumor tissues and TNBC tumor tissues. **E**: In the GSE161529 cohort, the UMAP plot shows the distribution of various cells in TNBC tissues (left). UMAP plot highlighting PYCR1 expression (middle). Violin plot of PYCR1 expression in different cells (right). **F**,** G**: Representative images of PYCR1 protein IHC staining and statistical analysis of IHC scores. **H**: Kaplan-Meier curves for OS in TNBC patients from the TCGA-BRCA dataset. **I**: Kaplan-Meier curves for PFI in TNBC patients from the TCGA-BRCA dataset, with optimal cutoff values of 6.266612. **J**: Kaplan-Meier curve for OS in TNBC patients from the Metabric dataset, with an optimal cutoff value of 7.730881. * *p* < 0.05, ** *p* < 0.01, *** *p* < 0.001

Univariate Cox regression analysis (Fig. 3B) indicated that among the four prognostic gene signatures, PYCR1 emerged as the most significant factor influencing patient OS. Thus, we focused on PYCR1 for further investigation and conducted a comprehensive evaluation of its role across various tumors through pan-cancer analysis (Figure S2). Compared to adjacent normal tissues, the mRNA expression level of PYCR1 was significantly upregulated in tumor tissues (Fig. 7A, B). Analysis of the TCGA and GSE76275 datasets demonstrated that the expression of PYCR1 in TNBC was significantly higher than in adjacent normal tissues and non-TNBC tumor tissues (Fig. 7C, D). Additionally, analysis of the GSE161529 dataset revealed that PYCR1 is highly expressed in tumor cells (Fig. 7E). At the protein expression level, IHC staining results from pathological sections of clinical patients confirmed that PYCR1 expression levels in different subtypes of breast cancer tissues were significantly elevated compared to adjacent normal tissues (Fig. 7F, G). Furthermore, in the TCGA dataset, high expression of the PYCR1 gene was significantly correlated with poorer overall survival (OS) and progression-free interval (PFI) (Fig. 7H, I). Similarly, in the Metabric dataset, elevated PYCR1 expression was associated with worse OS (Fig. 7J). Ultimately, we conducted a comprehensive assessment of PYCR1’s survival status across different datasets using the BEST analysis, revealing that high expression of PYCR1 was closely associated with a poorer prognosis, a finding consistent with our bioinformatics analysis results (Figure S3).

### The Potential Mechanisms of PYCR1 in TNBC

To further investigate the potential role of PYCR1 in the development of TNBC, TNBC patients from the TCGA database were stratified into high-expression and low-expression groups based on PYCR1 expression levels. We employed the ESTIMATE algorithm and CIBERSORT analysis to systematically evaluate the differences in immune microenvironment characteristics between the high-expression and low-expression subgroups, aiming to dissect the potential heterogeneity in the immune landscape. Our findings indicated that PYCR1 expression was positively correlated with M0 macrophages and negatively correlated with CD8 T cells (Figure S4). Subsequently, we conducted differential expression analysis using the “DESeq2” package, setting the significance level at *p-adj* 0.05, which led to the identification of a total of 862 DEGs (Table S6).

**Fig. 8:**
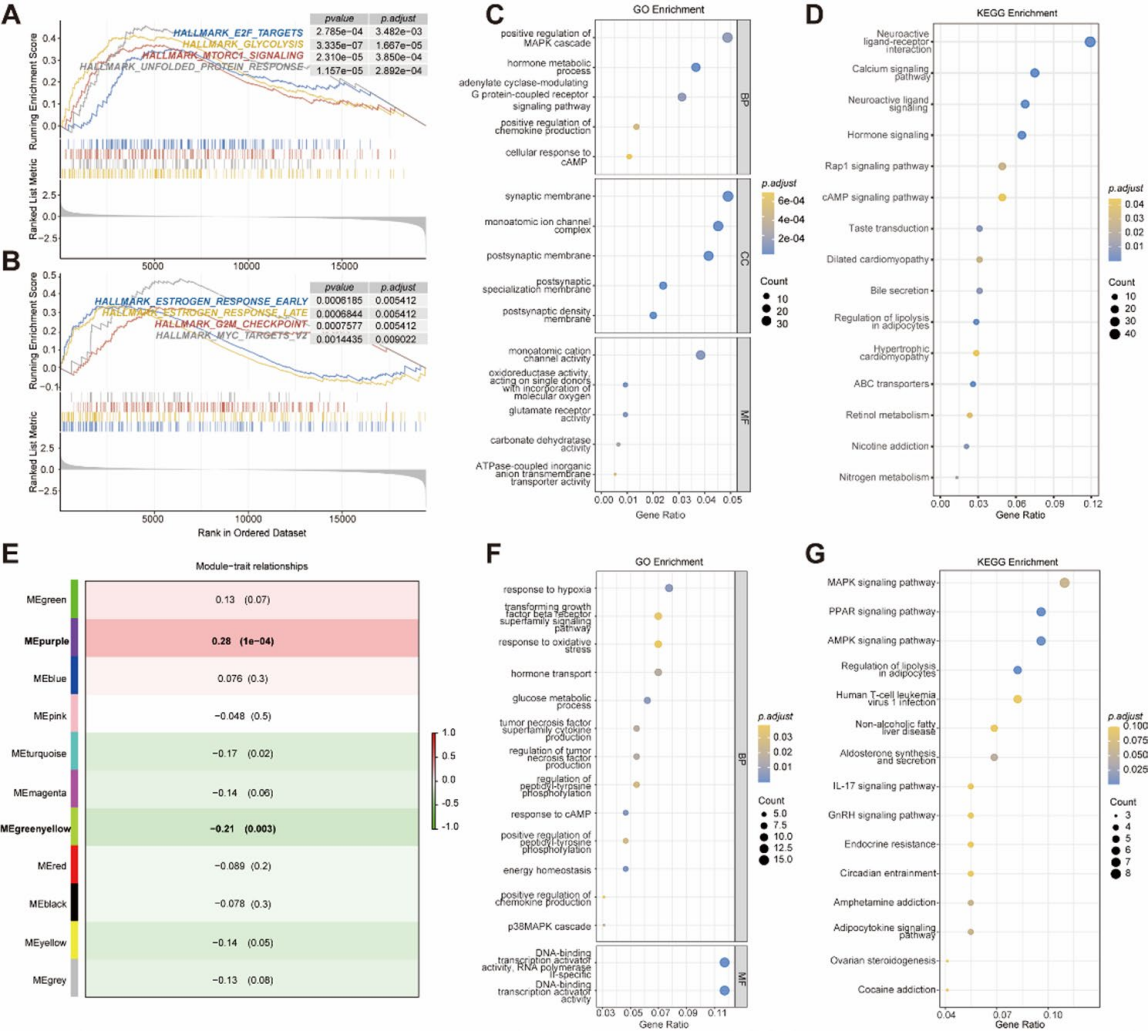
Potential mechanisms of PYCR1 in TNBC. **A**,** B**: GSEA enrichment analysis results for high and low PYCR1 expression groups.**C**: Bubble chart of GO functional enrichment analysis for 862 DEGs. **D**: Bubble chart of KEGG functional enrichment analysis for 862 DEGs. **E**: Heatmap showing the correlation between PYCR1 expression and modules. **F**: Bubble chart of GO functional enrichment analysis for 141 correlated genes. **G**: Bubble chart of KEGG functional enrichment analysis for 141 correlated genes

Additionally, we employed the GSEA method, with a particular focus on the HALLMARK gene sets, to investigate the potential functions of PYCR1 in specific biological processes. Our analysis revealed significant enrichment of several signaling pathways in the PYCR1 high-expression group, including glycolysis, the unfolded protein response, mTORC1 signaling, E2F targets, early estrogen response, late estrogen response, G2M checkpoint, and MYC targets V2 (Fig. 8A, B).

The results of the GO enrichment analysis suggested that PYCR1 may be associated with biological processes, including the positive regulation of the MAPK cascade, cellular response to cAMP, and ATPase-coupled inorganic anion transmembrane transporter activity (Fig. 8C). Furthermore, the KEGG analysis revealed that PYCR1 is involved in several signaling pathways, including the Rap1 signaling pathway, cAMP signaling pathway, Regulation of lipolysis in adipocytes, and Hormone signaling (Fig. 8D).

To further investigate the mechanisms by which PYCR1 influences TNBC, we conducted a WGCNA to identify genes associated with PYCR1 among the 6,223 DEGs in the TCGA-TNBC training cohort (Figure S5). Utilizing average linkage hierarchical clustering, we classified all DEGs into 11 distinct modules. Notably, the purple module, comprising 72 genes, exhibited the highest positive correlation with PYCR1 expression (*r* = 0.28, *p* < 0.0001), whereas the yellow-green module, consisting of 69 genes, displayed the highest negative correlation with PYCR1 expression (*r* = -0.21, *p* = 0.003) (Fig. 8E). The 141 genes derived from the purple and yellow-green modules were designated as PYCR1-related genes (Table S7) and were included in the subsequent enrichment analysis.

Based on the GO annotation analysis of the 141 genes related to PYCR1 (Fig. 8F), it is evident that PYCR1 is associated with pathways including the response to hypoxia, oxidative stress, and cyclic adenosine monophosphate (cAMP). The KEGG analysis (Fig. 8G) indicates that PYCR1 is involved in several biological processes, such as the Amphetamine addiction, Adipocytokine signaling pathway, Ovarian steroidogenesis, and Cocaine addiction. These findings provide novel insights into the biological functions of PYCR1 in the development of TNBC and may identify potential targets for future therapeutic strategies.

PYCR1 silencing inhibits TNBC cell growth in vitro and in vivo.

**Fig. 9:**
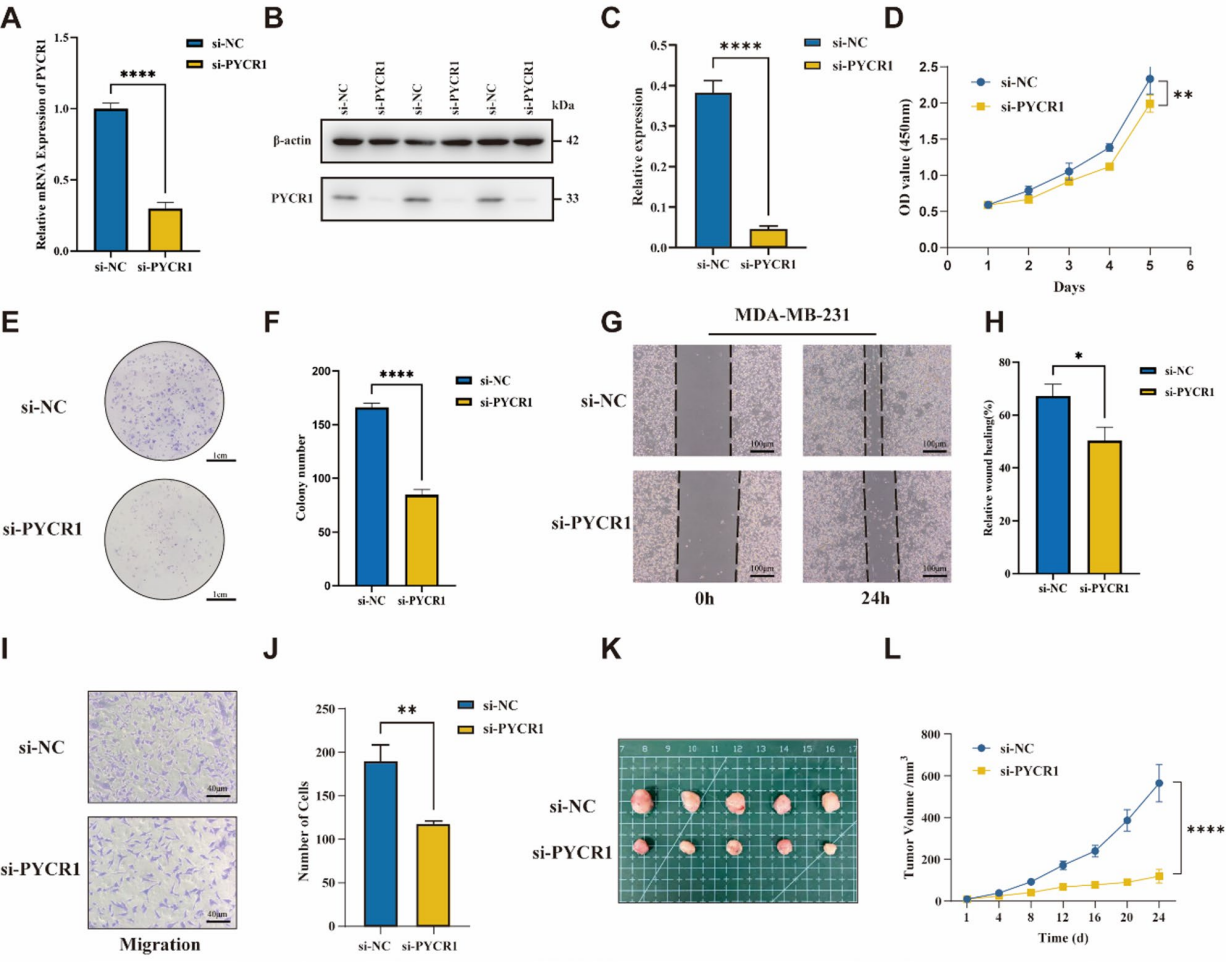
PYCR1 silencing inhibits TNBC cell growth in vitro and in vivo. **A**: Efficacy of siRNA in inhibiting PYCR1 expression by RT-qPCR. **B**,** C**: The Western blot analysis demonstrates the knockdown effect of PYCR1. **D**: CCK8 assay to evaluate the effect of PYCR1 knockdown on cell proliferation. **E**,** F**: Colony formation assay to assess the impact of PYCR1 knockdown on cell proliferation. Scale bar, 1 cm. **G**,** H**: Wound healing assay to evaluate the effect of PYCR1 knockdown on cell migration. Scale bar, 100 μm. **I**,** J**: Transwell assay to assess the migratory and invasive abilities of cells after PYCR1 knockdown. **K**: Gross morphology of tumors in BALB/c nude mice inoculated with MDA-MB-231 cells with or without PYCR1 knockdown. **L**: Serial monitoring of subcutaneous tumor volumes over a 24-day period. Scale bar, 40 μm.* *p* < 0.05, ** *p* < 0.01, *** *p* < 0.001, **** *p* < 0.0001. * All cellular assays were conducted using at least 3 biological replicates of knockdown samples, with each sample run in 3 technical replicates

To explore the effects of PYCR1 on TNBC in vitro, RNA interference experiments targeting PYCR1 were performed using the MDA-MB-231 cell line. The efficiency of gene silencing was confirmed using RT-qPCR (Fig. 9A) and Western blot assay (Fig. 9B, C), which demonstrated that the siRNA sequences effectively reduced PYCR1 expression. CCK-8 (Fig. 9D) and colony formation assays(Fig. 9E, F) revealed that knockdown of PYCR1 significantly decreased the proliferation and colony formation of breast cancer cells. In the wound-healing (Fig. 9G, H) and transwell assays (Fig. 9I, J), MDA-MB-231 cells with PYCR1 inhibition exhibited significantly slower migration and impaired invasive ability compared to the control group. In vivo studies, the tumors in the si-PYCR1 group exhibited significantly smaller volumes and lower net weights (Fig. 9K, L). These findings suggest that PYCR1 acts as a driver gene in TNBC, and its inhibition suppresses the malignant progression of TNBC cells.

## Discussion

In the development and progression of BC, numerous studies have elucidated the mechanisms underlying impaired mitochondrial function and ageing within the tumor microenvironment [22–24]. The association between mitochondrial dysfunction and the ageing process is still inadequately investigated, especially concerning TNBC. To date, there is a scarcity of studies that have thoroughly examined the genes that concurrently affect mitochondrial function and the ageing process. Furthermore, the influence of MAR-DEGs on the malignant progression of TNBC and the establishment of an immunosuppressive microenvironment has not been adequately explored.

This research presents a novel integration of mitochondrial-related genes with ageing gene sets to develop the inaugural risk prediction model tailored specifically for TNBC. Through an extensive bioinformatics analysis, we elucidate the roles of these genes within the TNBC tumor microenvironment, identifying PYCR1 as a significant target gene. We performed differential expression analysis to compare TNBC tumor tissues with adjacent non-tumor tissues, subsequently conducting GSEA to explore the underlying biological mechanisms. The findings indicated a notable activation of pathways linked to mitochondrial energy metabolism, such as oxidative phosphorylation, and ageing-related pathways, including the G2/M checkpoint pathway [25], within TNBC tumor tissues. Furthermore, pro-tumorigenic pathways, exemplified by the MYC pathway [26], were also activated, underscoring their essential contributions to the development and progression of TNBC. By intersecting DEGs with mitochondrial and ageing gene sets, we identified 52 MAR-DEGs that are specifically expressed in TNBC. To establish the MARS, we utilized batch log-rank tests, univariate Cox regression models, and the LASSO algorithm to screen the MAR-DEGs. Ultimately, we identified 4 MAR-DEGs for incorporation into the prognostic model. This innovative methodology not only enhances our understanding of the intricate relationship between mitochondrial dysfunction and ageing in TNBC but also provides a novel instrument for risk stratification and potential therapeutic intervention.

In this study, we evaluated the predictive performance of the MARS model using the TCGA-TNBC dataset as the training set and the GSE58812 dataset as the validation set. The results demonstrated that MARS achieved favorable AUC values, exceeding 0.75 in the training set and 0.60 in the validation set, thereby confirming its robust predictive accuracy across diverse datasets. To explore the correlation between MARS and the clinical characteristics of TNBC patients, we stratified patients into different risk groups based on MARS and analyzed their clinical features. A higher proportion of advanced-stage TNBC patients was observed in the high-risk group, indicating that MARS is associated with disease severity. To further enhance its predictive capability, we incorporated age and TNM stage into the MARS model. By combining the results of univariate and multivariate Cox regression analyses, we constructed a nomogram that integrates MARS with common clinical indicators of TNBC. After including these clinical characteristics, the AUC value increased to over 0.80, significantly improving the prognostic predictive power. Thus, the development of MARS and the nomogram provides a novel and effective framework for prognostic prediction in TNBC patients, offering valuable insights for clinical decision-making.

To investigate whether MARS can inform medication in TNBC patients, we analyzed the differences in mutational profiles, drug sensitivity, and immune checkpoint expression across different risk groups. Notably, TP53, a well-known tumor suppressor gene [27], exhibits the highest mutation frequency in both the high- and low-risk groups, consistent with its established role in TNBC. In the high TMB subgroup, there was no statistically significant difference in OS between the high-risk and low-risk groups (*p* = 0.667). However, in the low TMB subgroup, the low-risk group demonstrated superior OS compared to the high-risk group (*p* = 0.003). These findings suggest that integrating MARS with TMB may enhance prognostic prediction in TNBC patients, complementing the clinical utility of MARS [28–31]. Additionally, in our drug sensitivity research, we compared six commonly used chemotherapeutic agents for TNBC, including fluorouracil, paclitaxel, carboplatin, gemcitabine, docetaxel, and cyclophosphamide [32, 33]. Results indicated that, except for carboplatin and cyclophosphamide, the high-risk group exhibited significantly lower sensitivity to the other four drugs compared to the low-risk group. This suggests that MARS may serve as a crucial predictor for guiding chemotherapeutic agent selection, thereby improving treatment efficacy. Thus, MARS is not only a survival predictor but also a valuable tool for developing personalized treatment plans in TNBC.

To elucidate the prognostic differences between high- and low-risk TNBC patients, we conducted a systematic analysis of DEGs. The high-risk group exhibited significant enrichment in key pathways promoting tumor progression, such as the MAPK and TNF signaling pathways, which are closely associated with tumor proliferation, immune evasion, and drug resistance [34, 35]. GSEA further revealed that the high-risk group was highly active in pathways related to cell cycle regulation and DNA damage repair, whereas the low-risk group was enriched in pathways associated with immune surveillance, such as antigen presentation. These factors may be critical in contributing to the observed prognostic disparities [36, 37]. Notably, mitochondrial-related pathways, such as oxidative phosphorylation, were significantly activated in the high-risk group, highlighting the critical roles of mitochondrial dysfunction and ageing in TNBC progression [38]. These findings indicate that the MARS may influence TNBC prognosis by regulating senescence pathways related to mitochondrial function, providing a new theoretical basis for targeted therapeutic strategies.

Given that TNBC is classified as an immunologically “cold” tumor, the immunosuppressive environment within the tumor presents significant challenges for clinical immunotherapy [39]. To explore the impact of the MARS score on immunotherapy, we analyzed the relationship between immune infiltration and the MARS score. Results showed that low-risk patients had higher immune infiltration scores and greater tumor purity, indicating a more “active” immune microenvironment. Specifically, activated CD4 memory T cells and dendritic cells were more enriched in the low-risk group, enhancing anti-tumor immunity [40, 41]. Conversely, M2 macrophages and eosinophils were associated with higher risk scores and may promote tumor progression [42–44]. Identifying TNBC patients who may benefit from immunotherapy is a significant clinical challenge [45]. Additionally, low-risk patients exhibited higher expression levels of immune checkpoints and were more likely to benefit from immune checkpoint inhibitors [46]. These findings suggest that the MARS score could serve as a valuable biomarker for guiding immunotherapy in TNBC patients.

PYCR1 (Pyrroline-5-carboxylate reductase 1) is a mitochondrial enzyme encoded by a gene located on human chromosome 17 [47], catalyzing the final step of proline synthesis by converting pyrroline-5-carboxylate (P5C) to proline [47]. Research indicates that the PYCR1 gene is implicated in cancer progression through its regulation of mitochondrial energy metabolism processes [48]. For instance, PYCR1 enhances the growth and metastasis of liver cancer cells by modulating the expression of IRS1 via lactylation modification [49], and it promotes bladder cancer development by influencing the Akt/Wnt/β-catenin signaling pathway [50]. In TNBC, cancer-associated fibroblasts (CAFs) rely on PYCR1 for proline synthesis, which is crucial for tumor-promoting extracellular matrix deposition, as shown by scRNA-seq [51]. This study, for the first time, utilizes bioinformatics analysis and experimental validation to demonstrate that PYCR1 is highly expressed in TNBC and is closely associated with poor prognosis and the deterioration of clinical phenotypes. Our findings reveal that PYCR1 is expressed not only in CAFs but also at significant levels in tumor cells, suggesting its dual role in both stromal and cancer cells. These findings highlight the importance of targeting PYCR1 in TNBC therapy.

Despite the comprehensive analysis and promising findings of this study, several limitations should be acknowledged. First, the prognostic model was developed using retrospective data, which may limit its generalizability. Second, the in vitro experiments on PYCR1 require validation in in vivo models. Third, the bioinformatics analyses relied on publicly available datasets, which may have inherent limitations. Finally, further research is needed to explore the therapeutic potential of targeting PYCR1.

## Conclusion

In conclusion, this study establishes an effective prognostic model for TNBC based on four MAR-DEGs, elucidates their involvement in TNBC progression and immunosuppressive environment formation, and identifies PYCR1 as a key driver of TNBC malignancy and a potential therapeutic target.

## Supplementary Information

Below is the link to the electronic supplementary material.

Supplementary Material 1

Supplementary Material 2

Supplementary Material 3

Supplementary Material 4

Supplementary Material 5

Supplementary Material 6

Supplementary Material 7

Supplementary Material 8: Figure S1: The therapeutic benefit of the MARS value. A, E: The proportion of predicted immunotherapy responses in high-risk and low-risk subgroups. B, F: MARS of different immune response groups. C, D: Exclusion and Merck18 scores of high-risk and low-risk subgroups within the TCGA cohort. G, H: IFNG and Dysfunction scores of high-risk and low-risk subgroups within the GSE58812 cohort. I: Kaplan-Meier curves for DFI of TNBC patients within the TCGA and GSE58812 cohort, stratified by median MARS. J: Kaplan-Meier curves for MFS of TNBC patients within the GSE58812 cohort, stratified by median MARS. K, M: MARS of pre-chemotherapy and post-chemotherapy subgroups. L, N: Paired MARS of pre-chemotherapy and post-chemotherapy subgroups.

Supplementary Material 9: Figure S2: Comprehensive pan-cancer analysis of PYCR1. A: Pan-cancer expression of PYCR1 in 33 tumor samples (without normal samples). B: Correlation between PYCR1 and the proportions of immune cells across pan-cancer. C: Triangular representation of the correlation between PYCR1 and immune scores across pan-cancer. D: Heatmap of the correlation between PYCR1 and immune scores. E: Pan-cancer Cox regression analysis of PYCR1 in TCGA cancers. F: Radar plot of the correlation between PYCR1 expression and TMB.

Supplementary Material 10:Figure S3: Survival analysis of PYCR1 in different datasets. A: Kaplan-Meier curve for DFS of BC patients in the GSE45255 dataset. B: Kaplan-Meier curve for DSS of BC patients in the GSE45255 dataset. C: Kaplan-Meier curve for OS of BC patients in the GSE20685 dataset. D: Kaplan-Meier curve for OS of BC patients in the GSE42568 dataset. E: Kaplan-Meier curve for RFS of BC patients in the GSE42568 dataset. F: Kaplan-Meier curve for RFS of BC patients in the GSE17705 dataset. G: Kaplan-Meier curve for RFS of BC patients in the GSE45255 dataset. H: Kaplan-Meier curve for RFS of BC patients in the GSE20711 dataset

Supplementary Material 11: Figure S4: Immune cell landscape between high and low PYCR1 expression groups in the TCGA-TNBC cohort. A: Expression profiles of immune cells between high and low PYCR1 expression groups. B: Positive correlation between Macrophages M0 and PYCR1 expression. C: Negative correlation between T cells CD8 and PYCR1 expression. D: Stromal score. E: Immune score. F: ESTIMATE score. G: Tumor purity. *p < 0.05, ** p < 0.01, *** p < 0.001.

Supplementary Material 12: Figure S5: Identification of gene modules co-expressed with PYCR1 in the TCGA-TNBC cohort using WGCNA. A: Dendrogram of all DEGs based on their trend-adjusted expression profiles. B: Trend-adjusted expression profiles of DEGs, with colors assigned according to their module membership. C,D: Dendrogram of TNBC patients. 6223 genes were clustered into 11 modules based on dissimilarity measure (1-TOM). E: Clustering dendrogram of module eigengenes. F: Heatmap showing the clustering relationship between gene modules and samples. G: Heatmap of correlations between gene modules. H: Scatter plots of the purple module. I: Scatter plots of the purple module.

## Data Availability

The TCGA-TNBC dataset used in this study was obtained from the TCGA database. The GSE58812, GSE76275, and GSE161529 datasets were obtained from the GEO database. The METABRIC dataset was obtained from cBioPortal. All data and code required for the analyses are available upon request. For more information, please contact the corresponding author.

## Abbreviations

TNBC: Triple-negative breast cancer
BRCA: Breast Invasive Carcinoma
TCGA: The Cancer Genome Atlas
ER: Estrogen receptor
PR: Progesterone receptor
HER-2: Human Epidermal growth factor Receptor 2
MAR-DEGs: Mitochondrial and ageing-related differentially expressed genes
MARS: Mitochondrial ageing-related risk score model
CNV: Copy number variation
SASP: Senescence-associated secretory phenotype
GEO: Gene Expression Omnibus
RNA-seq: RNA sequencing
scRNA-seq: Single-cell RNA sequencing
BC: Breast cancer
DEGs: Differentially expressed genes
K-M: Kaplan-Meier
GO: Gene Ontology
BP: Biological Process
MF: Molecular Function
CC: Cellular Component
KEGG: Kyoto Encyclopedia of Genes and Genomes
GSEA: Gene Set Enrichment Analysis
FDR: False Discovery Rate
LASSO: Least Absolute Shrinkage and Selection Operator
ROC: Receiver Operating Characteristic
AUC: Area under the curve
TMB: Tumor Mutational Burden
MAF: Mutation Annotation Format
CIBERSORT: Cell-type Identification by Estimating Relative Subsets of RNA Transcripts
IHC: Immunohistochemistry
LogFC: Log Fold Change
WGCNA: Weighted Gene Co-expression Network Analysis
TOM: Topological overlap matrix
FBS: Fetal bovine serum
siRNA: Small interfering RNA
RT-qPCR: Real-time quantitative reverse transcription polymerase chain reaction
PBS: Phosphate-buffered saline
SD: Standard deviation
OS: Overall survival
ICI: Immune checkpoint inhibitors
IC50: Half-maximal inhibitory concentration
DFI: Disease-free interval
DSS: Disease-specific survival
PFI: Progression-free interval
