# Supplementary material for: Multi-omics analyses related to mitochondria and ageing in triple-negative breast cancer implicate PYCR1 potentiates tumor progression
Source: Cancer Cell Int. 2026 Feb 26;26:150. doi: 10.1186/s12935-026-04235-0 (PMC13041056; doi:10.1186/s12935-026-04235-0)
Supplement: Supplementary file 1 — Supplementary Material 1 [file 12935_2026_4235_MOESM1_ESM.docx]

**Table S1：** Baseline Characteristics of TCGA-TNBC Patients

| **Clinical parameters** | N | | value |
| --- | --- | --- | --- |
| **Age** |  |  | |
| ≤65 |  | 149 (78.01%) | |
| ＞65 |  | 42 (21.99%) | |
| **Stage** |  |  | |
| Stage I |  | 28 (14.66%) | |
| Stage II |  | 130 (68.06%) | |
| Stage III |  | 26 (13.61%) | |
| Stage IV |  | 3 (1.57%) | |
| Stage X |  | 1 (0.52%) | |
| Unkown |  | 3 (1.57%) | |
| **T.Stage** |  |  | |
| T1 |  | 41 (21.47%) | |
| T2 |  | 126 (65.97%) | |
| T3 |  | 18 (9.42%) | |
| T4 |  | 5 (2.62%) | |
| TX |  | 1 (0.52%) | |
| **N.Stage** |  |  | |
| N0 |  | 123 (64.4%) | |
| N1 |  | 46 (24.08%) | |
| N2 |  | 16 (8.38%) | |
| N3 |  | 6 (3.14%) | |
| **M.Stage** |  |  | |
| M0 |  | 160 (83.77%) | |
| M1 |  | 3 (1.57%) | |
| MX |  | 28 (14.66%) | |
