# Supplementary material for: Multi-omics analyses related to mitochondria and ageing in triple-negative breast cancer implicate PYCR1 potentiates tumor progression
Source: Cancer Cell Int. 2026 Feb 26;26:150. doi: 10.1186/s12935-026-04235-0 (PMC13041056; doi:10.1186/s12935-026-04235-0)
Supplement: Supplementary file 2 — Supplementary Material 2 [file 12935_2026_4235_MOESM2_ESM.docx]

**Table S3：** Mitochondrial and Aging-Associated Gene Catalog

| **Mitochondrial** | **Aging** |
| --- | --- |
| ABCF2 | ABL1 |
| ACBD6 | AC037482.3 |
| ACBD7 | ADA |
| ACE | ADM |
| ACE2 | ADRA1A |
| ACOT1 | AGER |
| ACOT12 | AKT1 |
| ADORA2A | AKT3 |
| AGTR1 | ALDH3A1 |
| AGTR2 | ALOX12 |
| AKT2 | AMFR |
| ALOX12 | AMH |
| ARL6IP5 | APAF1 |
| CAMK4 | APEX1 |
| CARM1 | APOD |
| CASP7 | APP |
| CAV3 | ARG1 |
| CCL27 | ARG2 |
| CGAS | ARNTL |
| CHD9 | ASS1 |
| CHKB | ATG7 |
| CREBBP | ATM |
| CRTC1 | ATP2B1 |
| CRTC2 | ATP8A2 |
| CRTC3 | ATR |
| CTSL | AURKB |
| DDX58 | B2M |
| ELAVL1 | BAK1 |
| G6PD | BCL2 |
| GABPB1-IT1 | BCL2A1 |
| GOT1 | BCL2L12 |
| HDAC3 | BCL6 |
| HELZ2 | BECN1 |
| HMGA1 | BGLAP |
| HSD17B12 | BMPR1A |
| IFIH1 | BRCA2 |
| IL1B | C1QA |
| IL6 | CACYBP |
| IRF7 | CALCA |
| MAPK11 | CALR |
| MDH1 | CARM1 |
| MED1 | CASP2 |
| MEF2C | CAT |
| MEF2D | CCL11 |
| MID1IP1 | CCN2 |
| MIR181B1 | CD68 |
| MIR210 | CDK6 |
| MT-TC | CDKN1A |
| MT-TI | CDKN2A |
| MT-TK | CDKN2B |
| MT-TP | CGAS |
| MT-TT | CHEK1 |
| MT-TV | CHEK2 |
| MYEF2 | CISD2 |
| NAMPT | CLDN1 |
| NCOA1 | CLN8 |
| NCOA2 | CNP |
| NCOA6 | CNR1 |
| NCOR1 | COL4A2 |
| NFKB2 | COMP |
| NMNAT2 | COQ7 |
| NOX1 | CREB1 |
| NR1D1 | CRYAB |
| OPRD1 | CTC1 |
| PCTP | CTNNA1 |
| PERM1 | CTSC |
| PIP5KL1 | CX3CL1 |
| PM20D1 | CYP1A1 |
| PPARA | DAG1 |
| PPP2R3C | DCN |
| PRKAA2 | DDC |
| PRKAB1 | DKK1 |
| PRKAB2 | DLD |
| PRKAG1 | DNAJA3 |
| PRKAG2 | DNMBP |
| PRKAG3 | DNMT3A |
| RB1 | ECRG4 |
| RELA | EDN1 |
| RNF122 | EDNRB |
| SLC2A1 | EEF1E1 |
| SLC2A4 | EEF2 |
| SMARCD3 | EIF2S1 |
| SNORD138 | ENDOG |
| SP1 | ENO3 |
| STARD3 | EPO |
| TBL1X | ERCC1 |
| TBL1XR1 | ERCC2 |
| TCL1A | ERO1A |
| TGS1 | FBXO4 |
| THRSP | FBXO5 |
| TLR3 | FOS |
| TLR7 | FOXG1 |
| TMPRSS2 | FOXM1 |
| TRAF6 | FOXO4 |
| USP46 | FZR1 |
| AARS1 | GBA |
| ABCE1 | GCLM |
| ABCG1 | GHRHR |
| ABCG2 | GJB2 |
| ABHD4 | GJB6 |
| ABHD5 | GLRX2 |
| ABHD6 | GNAO1 |
| ABHD8 | GNRH1 |
| ABL1 | GRB2 |
| ABLIM3 | GRM5 |
| ACAA2 | GSK3A |
| ACAT2 | GSN |
| ACBD3 | GSS |
| ACO1 | H2AX |
| ACO2 | HAMP |
| ACOD1 | HLA-G |
| ACOT11 | HMGA1 |
| ACOT13 | HMGA2 |
| ACSBG2 | HRAS |
| ACSL3 | HTR2A |
| ACSL4 | HTRA2 |
| ACSL5 | HYAL2 |
| ACSL6 | ICAM1 |
| ACSM1 | ID2 |
| ACSM2A | IDE |
| ACSM2B | IGFBP1 |
| ACSM4 | IGFBP2 |
| ACSM5 | IGFBP5 |
| ACSM6 | IL10 |
| ACSS1 | IL15 |
| ACSS2 | ING2 |
| ACTR10 | INPP5D |
| ADAM28 | IRAK1 |
| ADAP2 | ITGB2 |
| ADH5 | JUN |
| ADPRS | JUND |
| ADSS2 | KAT6A |
| AFG3L2 | KCNE2 |
| AGBL4 | KCNMB1 |
| AGPS | KIR2DL4 |
| AGTPBP1 | KL |
| AGXT2 | KMO |
| AIFM3 | KRAS |
| AIM2 | KRT14 |
| AIP | KRT16 |
| AK2 | KRT25 |
| AK4 | KRT33B |
| AK9 | KRT83 |
| AKAP8 | KRTAP4-3 |
| AKR1B10 | KRTAP4-5 |
| AKR1B15 | KRTAP4-8 |
| AKT1 | KRTAP4-9 |
| AKT3 | KYNU |
| ALAS2 | LEP |
| ALB | LIMS1 |
| ALDH18A1 | LITAF |
| ALDH1B1 | LMNA |
| ALDH5A1 | LONP1 |
| ALDH6A1 | LOXL2 |
| ALKBH3 | LRP1 |
| AMACR | LRRK2 |
| AMBRA1 | MAGEA2 |
| AMMECR1 | MAGEA2B |
| ANK2 | MAP2K1 |
| ANKRD37 | MAP3K3 |
| ANTKMT | MAPK1 |
| ANXA1 | MAPK14 |
| ANXA6 | MAPK3 |
| AP3B1 | MAPKAPK5 |
| APEX1 | MARCHF5 |
| APEX2 | MBD2 |
| APH1A | MBD3 |
| AQP8 | MIF |
| ARAF | MIR10A |
| ARG1 | MIR146A |
| ARGLU1 | MIR17 |
| ARID4B | MIR188 |
| ARIH2 | MIR20B |
| ARL2BP | MIR21 |
| ARMC1 | MIR217 |
| ARMC10 | MIR22 |
| ARMS2 | MIR34A |
| ARRB2 | MIR543 |
| ARSB | MIR590 |
| ASAH2 | MME |
| ASB9 | MMP7 |
| ASS1 | MNT |
| ATAD3A | MORC3 |
| ATAD3B | MPO |
| ATAD3C | MSH2 |
| ATCAY | MSH6 |
| ATF2 | MT-ATP6 |
| ATG12 | MT-CO1 |
| ATG13 | MT-ND4 |
| ATG14 | MTOR |
| ATG2A | NAPEPLD |
| ATG2B | NEK4 |
| ATG3 | NEK6 |
| ATG4D | NFE2L2 |
| ATG5 | NFKB2 |
| ATG7 | NOX4 |
| ATG9A | NPM1 |
| ATG9B | NPY2R |
| ATP13A2 | NPY5R |
| ATP2A1 | NQO1 |
| ATP5F1B | NR5A1 |
| ATP5F1C | NSMCE2 |
| ATP5F1E | NTRK1 |
| ATP5F1EP2 | NUAK1 |
| ATP5MC3 | NUDT1 |
| ATP5MGL | NUP62 |
| ATP5PO | OGG1 |
| ATP7A | OPA1 |
| ATP7B | P2RY1 |
| ATPAF1 | PAWR |
| ATXN3 | PAX2 |
| AURKAIP1 | PAX5 |
| AVP | PCK1 |
| AZIN2 | PDCD4 |
| BAG3 | PDGFRB |
| BAG4 | PDX1 |
| BAG5 | PENK |
| BAP1 | PICALM |
| BBC3 | PITX3 |
| BBOX1 | PLA2R1 |
| BCAP31 | PLK2 |
| BCAT1 | PML |
| BCKDK | PNPT1 |
| BCL2 | POLB |
| BCL2A1 | POLG |
| BCL2L1 | PPARGC1A |
| BCLAF3 | PPP1R9A |
| BDNF | PPP1R9B |
| BECN1 | PPP3CA |
| BHLHA15 | PRDM2 |
| BLID | PRELP |
| BLOC1S2 | PRKCD |
| BMF | PRKDC |
| BNIP1 | PRMT6 |
| BRAT1 | PRNP |
| BRD8 | PSEN1 |
| BRI3BP | PTEN |
| BRINP3 | PTH1R |
| BSG | RAD54B |
| BTD | RAD54L |
| C10orf67 | RBL1 |
| C10orf88 | RELA |
| C11orf65 | RETN |
| C12orf73 | RGN |
| C14orf119 | RNF165 |
| C15orf62 | ROMO1 |
| C19orf12 | RPN2 |
| C1orf43 | RPS6KB1 |
| C9orf72 | RSL1D1 |
| CABS1 | SCAP |
| CALM1 | SEC63 |
| CALM3 | SERPINE1 |
| CAMK2A | SERPINF1 |
| CAMKK2 | SIN3A |
| CAMKMT | SIRT1 |
| CAPN1 | SIRT3 |
| CAPN10 | SLC12A2 |
| CAPN2 | SLC30A10 |
| CAPRIN2 | SLC32A1 |
| CARD19 | SLC6A3 |
| CARS2 | SMC5 |
| CASP1 | SMC6 |
| CASP14 | SOD1 |
| CASP2 | SOD2 |
| CASP4 | SPI1 |
| CASP8AP2 | SREBF1 |
| CASQ1 | SRF |
| CAT | SRR |
| CAV2 | TACR3 |
| CAVIN1 | TBX2 |
| CCAR2 | TBX3 |
| CCDC51 | TERC |
| CCDC58 | TERF2 |
| CCK | TERT |
| CCM2 | TFCP2L1 |
| CCN6 | TGFB3 |
| CCNB1 | TGFBR2 |
| CCR7 | TH |
| CDC25C | TIMP1 |
| CDC37 | TIMP2 |
| CDK1 | TNFRSF1B |
| CDK5 | TP53 |
| CDK5RAP1 | TP63 |
| CDS2 | TPRA1 |
| CEBPA | TREX1 |
| CEBPZOS | TRPC6 |
| CEND1 | TSPO |
| CEP89 | TWIST1 |
| CERT1 | TYMS |
| CFAP410 | UCP2 |
| CFAP91 | UCP3 |
| CHCHD1 | ULK3 |
| CHCHD10 | VASH1 |
| CHCHD6 | VCAM1 |
| CHCHD7 | WNT1 |
| CHPF | WNT16 |
| CIAPIN1 | WRN |
| CIBAR1 | YBX1 |
| CIDEA | YPEL3 |
| CIDEB | ZKSCAN3 |
| CISD2 | ZMIZ1 |
| CKMT1B | ZMPSTE24 |
| CLIC1 | ZNF277 |
| CLIC4 | ZNF354A |
| CLN8 | ACSS2 |
| CLPX | ATP5MC3 |
| CLU | CA4 |
| CLUH | CALB1 |
| CMC1 | COL1A1 |
| CNP | COL3A1 |
| CNR1 | COL4A5 |
| COA3 | CX3CL1 |
| COA4 | DIABLO |
| COA5 | FABP3 |
| COL7A1 | GHITM |
| COQ10A | NDUFB11 |
| COQ10B | NREP |
| COQ3 | TFRC |
| COQ4 | UQCRFS1 |
| COQ5 | UQCRQ |
| COQ8B | ADIPOR2 |
| COQ9 | ANXA3 |
| COX11 | ANXA5 |
| COX17 | APOD |
| COX18 | B2M |
| COX19 | C1QA |
| COX20 | C1QB |
| COX4I1 | C1QC |
| COX4I2 | C3 |
| COX7A2L | C4A |
| COX7A2P2 | CLIC4 |
| COX7C | CLU |
| CPNE3 | CTSS |
| CPS1 | DCLK1 |
| CPT1B | DERL1 |
| CPT1C | EFCAB14 |
| CREB1 | EFEMP1 |
| CREB3L4 | FCGR2A |
| CREBZF | FCGR2B |
| CRYAB | GBP2 |
| CSNK2A2 | GFAP |
| CTNS | GNS |
| CTSK | GPNMB |
| CTTN | GSTA1 |
| CWC15 | H1-2 |
| CXADR | HBA1 |
| CYB5A | HCST |
| CYB5R1 | HLA-G |
| CYBA | IL33 |
| CYBB | JCHAIN |
| CYP1A1 | LAPTM5 |
| CYP1B1 | LGALS3 |
| CYP27A1 | LITAF |
| CYP27C1 | LYZ |
| CYP2D6 | MGST1 |
| CYP2D7 | MPEG1 |
| CYP2E1 | MSN |
| CYP2U1 | MT1F |
| CYRIB | NDRG1 |
| DAO | NPC2 |
| DAOA | PCSK6 |
| DBT | PSMD11 |
| DCAF8 | PTGES3 |
| DCK | RASA3 |
| DCN | RNF213 |
| DCPS | S100A4 |
| DCTPP1 | S100A6 |
| DDAH2 | SERPING1 |
| DDHD1 | SGK1 |
| DDHD2 | SPP1 |
| DDIT3 | TMED10 |
| DDIT4 | TXNIP |
| DDX1 | VAT1 |
| DDX21 | VWF |
| DDX6 | GHR |
| DECR1 | GHRH |
| DEGS1 | SHC1 |
| DEPP1 | POU1F1 |
| DGAT2 | PROP1 |
| DHFR | TP53 |
| DHFR2 | TERC |
| DHFRP1 | TERT |
| DHRS13 | ATM |
| DHX32 | PLAU |
| DHX36 | ERCC2 |
| DIMT1 | ERCC8 |
| DIP2A | WRN |
| DISC1 | LMNA |
| DLGAP5 | IGF1R |
| DNAJA1 | TXN |
| DNAJA3 | KL |
| DNAJC11 | E2F1 |
| DNAJC15 | PTPN11 |
| DNAJC19 | NFKB2 |
| DNAJC5 | STAT5B |
| DND1 | STAT3 |
| DNM1 | STAT5A |
| DNM2 | NRG1 |
| DNM3 | HDAC3 |
| DNMT1 | GH1 |
| DOK7 | IL7R |
| DUSP18 | IGF1 |
| DUSP21 | IGF2 |
| DYNLL1 | INS |
| DYNLT1 | NGF |
| E2F1 | IRS1 |
| EARS2 | PTPN1 |
| ECHDC2 | IRS2 |
| ECHS1 | AKT1 |
| EFHD1 | PIK3CB |
| EHHADH | NGFR |
| ELK1 | HRAS |
| ELK3 | MYC |
| ENOSF1 | EGFR |
| ENY2 | ERBB2 |
| EP300 | INSR |
| EPAS1 | NCOR1 |
| EPHA4 | NBN |
| ERAL1 | JUND |
| ERBB4 | IL2 |
| ERCC6L2 | PDGFB |
| ERN1 | EGF |
| ESR2 | IL2RG |
| ESRRA | FOS |
| ETFB | PDGFRB |
| ETFBKMT | EPOR |
| ETFDH | SST |
| ETNPPL | PRKCD |
| EXD2 | PPARA |
| EYA2 | RET |
| FADS1 | PLCG2 |
| FAM110B | PEX5 |
| FAM124B | TCF3 |
| FAM162A | PARP1 |
| FAM72A | BRCA1 |
| FANCG | PIN1 |
| FASTKD3 | PTEN |
| FASTKD5 | CREBBP |
| FATE1 | HIF1A |
| FBXL4 | UBB |
| FBXO7 | RPA1 |
| FBXW7 | BLM |
| FDX2 | BCL2 |
| FECH | S100B |
| FEN1 | VCP |
| FEZ1 | POLG |
| FGR | IGFBP3 |
| FH | HSP90AA1 |
| FHIT | NR3C1 |
| FIBP | EGR1 |
| FKBP4 | VEGFA |
| FLCN | ABL1 |
| FLVCR1 | BRCA2 |
| FNIP1 | TOP2A |
| FOXK2 | TOP2B |
| FOXO1 | NFKB1 |
| FOXO3 | TOP1 |
| FPGS | RAD51 |
| FTMT | UBE2I |
| FUNDC1 | TNF |
| FUNDC2 | PDPK1 |
| FUS | CEBPA |
| FXN | CEBPB |
| FYN | MXI1 |
| FZD5 | TGFB1 |
| FZD9 | ERCC6 |
| G0S2 | STK11 |
| GABARAP | EP300 |
| GABARAPL1 | APTX |
| GABARAPL2 | PML |
| GABARAPL3 | GSK3B |
| GABPA | HTT |
| GABPB1 | PRKCA |
| GADD45GIP1 | SSTR3 |
| GARS1 | HELLS |
| GATB | APOC3 |
| GATD3B | EEF2 |
| GATM | ERCC3 |
| GBA | TERF1 |
| GCAT | PRKDC |
| GCK | CAT |
| GCKR | ERCC5 |
| GCLM | AR |
| GCSH | GTF2H2 |
| GDAP1 | XRCC5 |
| GDF5-AS1 | PCNA |
| GFM1 | FEN1 |
| GGCT | FAS |
| GGNBP1 | TERF2 |
| GHITM | XRCC6 |
| GIMAP8 | POLD1 |
| GIT1 | BAX |
| GJA1 | RB1 |
| GK | EMD |
| GK2 | GRB2 |
| GK5 | FOXO3 |
| GLRX2 | FOXO1 |
| GLRX5 | HSF1 |
| GLUD2 | XPA |
| GLUL | MSRA |
| GLYAT | RECQL4 |
| GLYATL1 | SOD2 |
| GLYATL1B | SOD1 |
| GLYATL2 | FOXM1 |
| GLYATL3 | COQ7 |
| GNE | CACNA1A |
| GNPAT | LRP2 |
| GNRH1 | AIFM1 |
| GOLPH3 | UCHL1 |
| GOT2 | APP |
| GPAA1 | APOE |
| GPER1 | A2M |
| GPN1 | SNCG |
| GPS2 | PRDX1 |
| GRAMD4 | PON1 |
| GRPEL1 | RELA |
| GRSF1 | IL6 |
| GSK3A | RGN |
| GSK3B | ATP5O |
| GSTP1 | RAD52 |
| GSTZ1 | TOP3B |
| GTF3C4 | ERCC1 |
| GTPBP10 | SIRT1 |
| GTPBP3 | HDAC1 |
| GTPBP8 | HSPA9 |
| GUF1 | GPX1 |
| GYG1 | GSR |
| GZMB | GSS |
| H6PD | GSTA4 |
| HADHA | GSTP1 |
| HAGH | MT-CO1 |
| HAP1 | HSPD1 |
| HARS1 | HSPA1A |
| HAUS3 | HSPA1B |
| HAX1 | PCMT1 |
| HCFC1 | MAPK8 |
| HCLS1 | YWHAZ |
| HDAC6 | PTK2B |
| HEATR1 | PTK2 |
| HEBP2 | IL7 |
| HEMK1 | MAPK14 |
| HGF | FGFR1 |
| HIF1A | SP1 |
| HIF3A | FLT1 |
| HIGD1A | JUN |
| HIGD1B | MED1 |
| HIGD1C | MAPK9 |
| HIGD2A | MAPK3 |
| HIGD2B | HMGB1 |
| HIP1R | CCNA2 |
| HIVEP1 | HMGB2 |
| HJURP | MAP3K5 |
| HK1 | TAF1 |
| HK2 | LMNB1 |
| HK3 | SDHC |
| HKDC1 | FOXO4 |
| HLCS | HESX1 |
| HMGN5 | PIK3R1 |
| HPS4 | BSCL2 |
| HRK | AGPAT2 |
| HS1BP3 | BMI1 |
| HSD3B1 | EEF1A1 |
| HSD3B2 | TFAP2A |
| HSP90AA1 | BDNF |
| HSP90AB1 | CREB1 |
| HSP90B2P | ATF2 |
| HSPA13 | TBP |
| HSPA1A | APEX1 |
| HSPA1B | HBP1 |
| HSPA1L | BUB1B |
| HSPA4 | PTGS2 |
| HSPA5 | HSPA8 |
| HSPA9 | SIN3A |
| HTD2 | CDK1 |
| HTT | TFDP1 |
| HUWE1 | DDIT3 |
| HYKK | POLA1 |
| IAPP | MAPT |
| IBA57 | CTGF |
| IDE | HDAC2 |
| IDH1 | MAX |
| IDH3G | MXD1 |
| IFI27 | MDM2 |
| IFI27L1 | SUMO1 |
| IFI27L2 | H2AFX |
| IFI6 | HOXB7 |
| IFIT2 | HOXC4 |
| IFIT3 | JAK2 |
| IGF1 | ESR1 |
| IKBKE | LEP |
| ILF3 | LEPR |
| IMMP1L | NFKBIA |
| IMMP2L | CLU |
| IMMT | MTOR |
| INF2 | GHRHR |
| IQCN | CTNNB1 |
| IREB2 | PSEN1 |
| IRF3 | DLL3 |
| IRGM | CDKN2A |
| ISCU | PPP1CA |
| JARID2 | DBN1 |
| JTB | NOG |
| JUN | ELN |
| KANK2 | ATR |
| KAT2A | UCP3 |
| KCNJ11 | ZMPSTE24 |
| KCNJ8 | TP63 |
| KDR | UCP2 |
| KIF1B | POLB |
| KIF28P | GCLC |
| KIFBP | GCLM |
| KLC2 | SIRT6 |
| KLK6 | BUB3 |
| KMO | RAE1 |
| KRAS | PMCH |
| KYAT1 | MLH1 |
| KYAT3 | CSNK1E |
| KYNU | STUB1 |
| LACTB | PPM1D |
| LACTB2 | CHEK2 |
| LARS2 | PCK1 |
| LDHAL6B | ARHGAP1 |
| LDHD | CDC42 |
| LEPROT | ARNTL |
| LGALS3 | CLOCK |
| LIG1 | HIC1 |
| LIPF | PAPPA |
| LIPT1 | ADCY5 |
| LMAN1 | PPARGC1A |
| LPIN1 | GPX4 |
| LRPPRC | UCP1 |
| LRRC10 | FGF23 |
| LRRC59 | EFEMP1 |
| LRRK1 | ERCC4 |
| LRRK2 | CETP |
| LYN | PPARG |
| MACC1 | AGTR1 |
| MAFF | CISD2 |
| MALSU1 | EEF1E1 |
| MAN2A1 | EPS8 |
| MAP1B | KCNA3 |
| MAP1LC3A | SIRT7 |
| MAP1LC3B | SLC13A1 |
| MAP1LC3B2 | SOCS2 |
| MAP1LC3C | TPP2 |
| MAP1S | TP53BP1 |
| MAP2K1 | SIRT3 |
| MAP2K2 | NCOR2 |
| MAPK1 | SUN1 |
| MAPK10 | BAK1 |
| MAPK12 | IGFBP2 |
| MAPK14 | PYCR1 |
| MAPK3 | TP73 |
| MAPK8 | CNR1 |
| MAPK8IP1 | NFE2L2 |
| MAPK9 | CDKN1A |
| MAPT | PDGFRA |
| MARK1 | PIK3CA |
| MARK2 | C1QA |
| MAVS | CDKN2B |
| MCCC1 | EIF5A2 |
| MCCD1 | MIF |
| MCEE | DGAT1 |
| MCL1 | MT1E |
| MCUB | FGF21 |
| ME1 | HTRA2 |
| ME2 | GSK3A |
| MEF2A | NUDT1 |
| MGARP | IKBKB |
| MICALL2 | SQSTM1 |
| MICOS10 | CDK7 |
| MICOS13 | GRN |
| MICU3 | SERPINE1 |
| MIGA1 | SPRTN |
| MIR144 | RICTOR |
| MIR17 | CTF1 |
| MIR29A | TRAP1 |
| MIR29B1 | TRPV1 |
| MIR29C | NFE2L1 |
| MLLT11 | IFNB1 |
| MLXIP | GDF11 |
| MMAA |  |
| MMAB |  |
| MMP1 |  |
| MMP2 |  |
| MMP9 |  |
| MOAP1 |  |
| MOBP |  |
| MPC1 |  |
| MPC1L |  |
| MPC2 |  |
| MPG |  |
| MRM3 |  |
| MRPL10 |  |
| MRPL18 |  |
| MRPL19 |  |
| MRPL20 |  |
| MRPL21 |  |
| MRPL24 |  |
| MRPL32 |  |
| MRPL51 |  |
| MRPL52 |  |
| MRPL58 |  |
| MRPS17 |  |
| MRPS18B |  |
| MRPS28 |  |
| MRPS30 |  |
| MRPS34 |  |
| MRPS35 |  |
| MRPS36 |  |
| MSRB2 |  |
| MSTO1 |  |
| MTCH2 |  |
| MT-CO1 |  |
| MT-CO2 |  |
| MTCO2P12 |  |
| MTERF2 |  |
| MTERF3 |  |
| MTERF4 |  |
| MTG2 |  |
| MTHFD1 |  |
| MTHFD1L |  |
| MTLN |  |
| MTM1 |  |
| MT-ND5 |  |
| MTO1 |  |
| MTOR |  |
| MTRF1L |  |
| MT-RNR1 |  |
| MT-RNR2 |  |
| MTRNR2L5 |  |
| MT-TE |  |
| MT-TF |  |
| MT-TH |  |
| MT-TL1 |  |
| MT-TL2 |  |
| MT-TN |  |
| MT-TQ |  |
| MT-TS2 |  |
| MT-TW |  |
| MTUS1 |  |
| MTX3 |  |
| MX1 |  |
| MX2 |  |
| MXD1 |  |
| MYCBP |  |
| MYH14 |  |
| MYH7 |  |
| MYL10 |  |
| MYO19 |  |
| MYOC |  |
| MYOM2 |  |
| NACC2 |  |
| NAIF1 |  |
| NAPG |  |
| NAXD |  |
| NAXE |  |
| NCBP1 |  |
| NCSTN |  |
| NDFIP2 |  |
| NDRG4 |  |
| NDUFA3 |  |
| NDUFA4L2 |  |
| NDUFAF2 |  |
| NDUFAF3 |  |
| NDUFAF5 |  |
| NDUFB1 |  |
| NDUFB10 |  |
| NDUFB11 |  |
| NDUFB4 |  |
| NDUFB5 |  |
| NDUFC2-KCTD14 |  |
| NDUFS5 |  |
| NDUFS6 |  |
| NDUFS8 |  |
| NEB |  |
| NECTIN2 |  |
| NEFL |  |
| NENF |  |
| NFKB1 |  |
| NFS1 |  |
| NGB |  |
| NGDN |  |
| NIPSNAP1 |  |
| NIPSNAP2 |  |
| NIPSNAP3A |  |
| NLRP5 |  |
| NME1 |  |
| NME2 |  |
| NMT1 |  |
| NOC3L |  |
| NOD2 |  |
| NOL3 |  |
| NOL6 |  |
| NOL7 |  |
| NOS1 |  |
| NOS1AP |  |
| NOS3 |  |
| NOX4 |  |
| NPEPPS |  |
| NR3C1 |  |
| NR4A1 |  |
| NRF1 |  |
| NRGN |  |
| NRP1 |  |
| NT5M |  |
| NTHL1 |  |
| NTSR1 |  |
| NUDT1 |  |
| NUDT13 |  |
| OAS1 |  |
| OGT |  |
| OLFM4 |  |
| OPTN |  |
| OXCT1 |  |
| P2RY1 |  |
| P2RY12 |  |
| P4HA1 |  |
| PACS2 |  |
| PAGE4 |  |
| PAK5 |  |
| PAM16 |  |
| PANK2 |  |
| PARG |  |
| PARL |  |
| PARP1 |  |
| PARP9 |  |
| PC |  |
| PCCB |  |
| PCF11 |  |
| PCK1 |  |
| PDCD5 |  |
| PDE12 |  |
| PDK2 |  |
| PDK3 |  |
| PDP2 |  |
| PDPN |  |
| PDSS2 |  |
| PDZD8 |  |
| PECR |  |
| PEMT |  |
| PERP |  |
| PET100 |  |
| PEX5 |  |
| PFDN2 |  |
| PFDN4 |  |
| PGR |  |
| PGRMC1 |  |
| PHYKPL |  |
| PI4K2A |  |
| PI4KB |  |
| PID1 |  |
| PIN1 |  |
| PIN4 |  |
| PINX1 |  |
| PKM |  |
| PLA2G2A |  |
| PLA2G4A |  |
| PLA2G4B |  |
| PLA2G4C |  |
| PLA2G4F |  |
| PLA2G6 |  |
| PLAUR |  |
| PLEKHN1 |  |
| PLIN5 |  |
| PLN |  |
| PMAIP1 |  |
| PNKD |  |
| PNKP |  |
| PNPLA4 |  |
| POLR1G |  |
| POU5F1 |  |
| POU5F1B |  |
| PPA2 |  |
| PPARGC1A |  |
| PPARGC1B |  |
| PPIF |  |
| PPM1B |  |
| PPM1E |  |
| PPP1CC |  |
| PPP1R13B |  |
| PPP1R15A |  |
| PPP2CA |  |
| PPP2CB |  |
| PPP2R1A |  |
| PPP2R2B |  |
| PPP3CA |  |
| PPP3CC |  |
| PPP3R1 |  |
| PPP6C |  |
| PPRC1 |  |
| PPTC7 |  |
| PRDX5 |  |
| PRDX6 |  |
| PRELID1 |  |
| PRELID2 |  |
| PRELID3A |  |
| PRELID3B |  |
| PRIMPOL |  |
| PRKAA1 |  |
| PRKACA |  |
| PRKCA |  |
| PRKCD |  |
| PRKCE |  |
| PRMT6 |  |
| PRORP |  |
| PSEN1 |  |
| PSEN2 |  |
| PSMB4 |  |
| PSMD10 |  |
| PTCD1 |  |
| PTCD2 |  |
| PTEN |  |
| PTGES2 |  |
| PTPMT1 |  |
| PTPN1 |  |
| PTPN11 |  |
| PTS |  |
| PUS10 |  |
| PYCARD |  |
| PYCR2 |  |
| PYROXD2 |  |
| QARS1 |  |
| QRSL1 |  |
| QTRT2 |  |
| RAB11FIP3 |  |
| RAB11FIP5 |  |
| RAB29 |  |
| RAB32 |  |
| RAB38 |  |
| RAB3A |  |
| RAB40AL |  |
| RAC2 |  |
| RACK1 |  |
| RAD51 |  |
| RAD51C |  |
| RAF1 |  |
| RANBP2 |  |
| RAP1GDS1 |  |
| RARS2 |  |
| RB1CC1 |  |
| REEP1 |  |
| RFK |  |
| RGS2 |  |
| RHBDD1 |  |
| RHOT1 |  |
| RHOT2 |  |
| RHOU |  |
| RIDA |  |
| RIPK1 |  |
| RMDN2 |  |
| RMDN3 |  |
| RMRP |  |
| RNASEL |  |
| RNASET2 |  |
| RNF144B |  |
| RNF185 |  |
| RNF186 |  |
| RNF31 |  |
| RNF41 |  |
| RNF5 |  |
| RPS27A |  |
| RPS3 |  |
| RPS6KA6 |  |
| RPS6KB1 |  |
| RPUSD3 |  |
| RPUSD4 |  |
| RRM2B |  |
| RSAD1 |  |
| RSAD2 |  |
| RTL10 |  |
| RTN4IP1 |  |
| RXRA |  |
| S1PR4 |  |
| SACS |  |
| SAE1 |  |
| SARM1 |  |
| SARS2 |  |
| SCCPDH |  |
| SCP2 |  |
| SDHA |  |
| SDHAF2 |  |
| SDHAF3 |  |
| SDHAF4 |  |
| SDHB |  |
| SDHD |  |
| SDS |  |
| SECISBP2 |  |
| SELENOO |  |
| SEPTIN4 |  |
| SERAC1 |  |
| SESN2 |  |
| SFN |  |
| SFXN1 |  |
| SFXN2 |  |
| SFXN3 |  |
| SFXN4 |  |
| SFXN5 |  |
| SGK1 |  |
| SH3BP5 |  |
| SH3GLB1 |  |
| SHARPIN |  |
| SHC1 |  |
| SHMT2 |  |
| SIAH3 |  |
| SIRT1 |  |
| SIRT2 |  |
| SIRT3 |  |
| SIRT4 |  |
| SIRT5 |  |
| SIRT7 |  |
| SLC11A2 |  |
| SLC19A3 |  |
| SLC22A4 |  |
| SLC22A5 |  |
| SLC25A1 |  |
| SLC25A10 |  |
| SLC25A13 |  |
| SLC25A14 |  |
| SLC25A15 |  |
| SLC25A16 |  |
| SLC25A19 |  |
| SLC25A2 |  |
| SLC25A20 |  |
| SLC25A21 |  |
| SLC25A22 |  |
| SLC25A23 |  |
| SLC25A24 |  |
| SLC25A25 |  |
| SLC25A27 |  |
| SLC25A29 |  |
| SLC25A3 |  |
| SLC25A30 |  |
| SLC25A31 |  |
| SLC25A32 |  |
| SLC25A33 |  |
| SLC25A34 |  |
| SLC25A35 |  |
| SLC25A36 |  |
| SLC25A39 |  |
| SLC25A4 |  |
| SLC25A43 |  |
| SLC25A44 |  |
| SLC25A45 |  |
| SLC25A46 |  |
| SLC25A47 |  |
| SLC25A48 |  |
| SLC25A5 |  |
| SLC25A52 |  |
| SLC25A6 |  |
| SLC27A1 |  |
| SLC27A3 |  |
| SLC34A1 |  |
| SLC35F6 |  |
| SLC39A8 |  |
| SLC3A1 |  |
| SLC44A1 |  |
| SLC44A2 |  |
| SLC4A5 |  |
| SLC8A1 |  |
| SLC8A3 |  |
| SLC8B1 |  |
| SLC9A1 |  |
| SLC9B2 |  |
| SLIRP |  |
| SLIT3 |  |
| SMCP |  |
| SMDT1 |  |
| SMURF1 |  |
| SNAP23 |  |
| SNCA |  |
| SNN |  |
| SNPH |  |
| SOD1 |  |
| SOD2 |  |
| SORD |  |
| SOX10 |  |
| SOX4 |  |
| SP140 |  |
| SPART |  |
| SPAST |  |
| SPATA18 |  |
| SPATA19 |  |
| SPATA5 |  |
| SPATA7 |  |
| SPHKAP |  |
| SPIRE1 |  |
| SPNS1 |  |
| SQSTM1 |  |
| SRC |  |
| SREBF1 |  |
| SREBF2 |  |
| SRI |  |
| STAP1 |  |
| STARD13 |  |
| STARD7 |  |
| STAT2 |  |
| STING1 |  |
| STK11 |  |
| STMP1 |  |
| STOM |  |
| STOML2 |  |
| STOX1 |  |
| STPG1 |  |
| STX17 |  |
| STXBP1 |  |
| STYXL1 |  |
| SUCLG2 |  |
| SUGCT |  |
| SUOX |  |
| SUPV3L1 |  |
| SURF1 |  |
| SYBU |  |
| SYNE2 |  |
| TACO1 |  |
| TARDBP |  |
| TAT |  |
| TAZ |  |
| TBK1 |  |
| TBRG4 |  |
| TCAIM |  |
| TCHP |  |
| TDH |  |
| TDRD7 |  |
| TERT |  |
| TEX10 |  |
| TFAP2C |  |
| TFAP4 |  |
| TFDP1 |  |
| TFDP2 |  |
| TFRC |  |
| TGM2 |  |
| TH |  |
| THOP1 |  |
| TICAM1 |  |
| TIGAR |  |
| TIMM21 |  |
| TIMM22 |  |
| TIMM23B |  |
| TIMM29 |  |
| TIMM44 |  |
| TIMMDC1 |  |
| TLE6 |  |
| TMBIM6 |  |
| TMEM102 |  |
| TMEM135 |  |
| TMEM14A |  |
| TMEM14B |  |
| TMEM14DP |  |
| TMEM14EP |  |
| TMEM186 |  |
| TMEM71 |  |
| TMEM8B |  |
| TMLHE |  |
| TMX2 |  |
| TNFRSF1A |  |
| TNFSF10 |  |
| TNRC18 |  |
| TOMM20 |  |
| TOMM34 |  |
| TOMM7 |  |
| TOMM70 |  |
| TOP1MT |  |
| TOP3A |  |
| TP53 |  |
| TP53AIP1 |  |
| TP53BP2 |  |
| TP63 |  |
| TP73 |  |
| TPPP |  |
| TRAF3 |  |
| TRAF3IP3 |  |
| TRAK1 |  |
| TRAK2 |  |
| TRAP1 |  |
| TREM2 |  |
| TRIAP1 |  |
| TRIM14 |  |
| TRIM31 |  |
| TRIM39 |  |
| TRMT10A |  |
| TRMT10B |  |
| TRMT5 |  |
| TRMT61B |  |
| TRMU |  |
| TRNT1 |  |
| TRUB1 |  |
| TRUB2 |  |
| TSC2 |  |
| TSFM |  |
| TSPOAP1 |  |
| TTC19 |  |
| TTN |  |
| TUFM |  |
| TUSC2 |  |
| TUSC3 |  |
| TWNK |  |
| TXNIP |  |
| TXNRD3 |  |
| TYMP |  |
| TYMS |  |
| UBA1 |  |
| UBA52 |  |
| UBB |  |
| UBC |  |
| UBE2D3 |  |
| UBE2J2 |  |
| UBE2L3 |  |
| UBIAD1 |  |
| UBL4B |  |
| UBL5 |  |
| UCP1 |  |
| UCP2 |  |
| ULK1 |  |
| UQCC1 |  |
| UQCC3 |  |
| UQCR10 |  |
| UQCR11 |  |
| UQCRB |  |
| UQCRC2 |  |
| UQCRFS1 |  |
| UQCRFS1P1 |  |
| UQCRH |  |
| UQCRHL |  |
| UQCRQ |  |
| URI1 |  |
| UROS |  |
| USP15 |  |
| USP36 |  |
| USP48 |  |
| UXT |  |
| VAMP1 |  |
| VASN |  |
| VAT1 |  |
| VCP |  |
| VDAC1 |  |
| VDAC3 |  |
| VHL |  |
| VPS11 |  |
| VPS13A |  |
| VPS13C |  |
| VPS13D |  |
| VPS35 |  |
| VRK2 |  |
| VWA8 |  |
| WASF1 |  |
| WDR26 |  |
| WDR35 |  |
| WDR45 |  |
| WDR45B |  |
| WDR81 |  |
| WDR93 |  |
| WIPI1 |  |
| WIPI2 |  |
| WWOX |  |
| XAF1 |  |
| XPC |  |
| XRCC3 |  |
| YJEFN3 |  |
| YKT6 |  |
| YME1L1 |  |
| YWHAB |  |
| YWHAE |  |
| YWHAG |  |
| YWHAH |  |
| YWHAQ |  |
| YWHAZ |  |
| ZBED3 |  |
| ZBTB6 |  |
| ZDHHC6 |  |
| ZDHHC8 |  |
| ZFYVE1 |  |
| ZMIZ2 |  |
| ZNF205 |  |
| ZNF217 |  |
| AADAT |  |
| AARS2 |  |
| AASS |  |
| ABAT |  |
| ABCA9 |  |
| ABCB10 |  |
| ABCB6 |  |
| ABCB7 |  |
| ABCB8 |  |
| ABCD1 |  |
| ABCD2 |  |
| ABCD3 |  |
| ABHD10 |  |
| ABHD11 |  |
| ACAA1 |  |
| ACACA |  |
| ACACB |  |
| ACAD10 |  |
| ACAD11 |  |
| ACAD8 |  |
| ACAD9 |  |
| ACADL |  |
| ACADM |  |
| ACADS |  |
| ACADSB |  |
| ACADVL |  |
| ACAT1 |  |
| ACCS |  |
| ACLY |  |
| ACOT2 |  |
| ACOT7 |  |
| ACOT9 |  |
| ACP6 |  |
| ACSF2 |  |
| ACSF3 |  |
| ACSL1 |  |
| ACSM3 |  |
| ACSS3 |  |
| ADCK1 |  |
| ADCK2 |  |
| ADCK5 |  |
| ADCY10 |  |
| ADHFE1 |  |
| AFG1L |  |
| AGK |  |
| AGMAT |  |
| AGPAT4 |  |
| AGPAT5 |  |
| AGXT |  |
| AHCYL1 |  |
| AIFM1 |  |
| AIFM2 |  |
| AK3 |  |
| AKAP1 |  |
| AKAP10 |  |
| AKR7A2 |  |
| ALAS1 |  |
| ALDH1L1 |  |
| ALDH1L2 |  |
| ALDH2 |  |
| ALDH3A2 |  |
| ALDH4A1 |  |
| ALDH7A1 |  |
| ALDH9A1 |  |
| ALKBH1 |  |
| ALKBH7 |  |
| AMT |  |
| ANGEL2 |  |
| APOO |  |
| APOOL |  |
| ARF5 |  |
| ARG2 |  |
| ARL2 |  |
| ARMCX1 |  |
| ARMCX2 |  |
| ARMCX3 |  |
| ARMCX6 |  |
| ATAD1 |  |
| ATP23 |  |
| ATP5F1A |  |
| ATP5F1D |  |
| ATP5IF1 |  |
| ATP5MC1 |  |
| ATP5MC2 |  |
| ATP5MD |  |
| ATP5ME |  |
| ATP5MF |  |
| ATP5MF-PTCD1 |  |
| ATP5MG |  |
| ATP5MPL |  |
| ATP5PB |  |
| ATP5PD |  |
| ATP5PF |  |
| ATPAF2 |  |
| ATPSCKMT |  |
| AUH |  |
| BAD |  |
| BAK1 |  |
| BAX |  |
| BCAT2 |  |
| BCKDHA |  |
| BCKDHB |  |
| BCL2L10 |  |
| BCL2L11 |  |
| BCL2L13 |  |
| BCL2L2 |  |
| BCO2 |  |
| BCS1L |  |
| BDH1 |  |
| BID |  |
| BIK |  |
| BLOC1S1 |  |
| BNIP3 |  |
| BNIP3L |  |
| BOK |  |
| BOLA1 |  |
| BOLA3 |  |
| BPHL |  |
| C12orf65 |  |
| C15orf48 |  |
| C15orf61 |  |
| C16orf91 |  |
| C1QBP |  |
| C2orf69 |  |
| C3orf33 |  |
| C5orf63 |  |
| C6orf136 |  |
| C8orf82 |  |
| CA5A |  |
| CA5B |  |
| CASP3 |  |
| CASP8 |  |
| CASP9 |  |
| CBR3 |  |
| CBR4 |  |
| CCDC127 |  |
| CCDC90B |  |
| CHCHD2 |  |
| CHCHD3 |  |
| CHCHD4 |  |
| CHCHD5 |  |
| CHDH |  |
| CHPT1 |  |
| CISD1 |  |
| CISD3 |  |
| CKMT1A |  |
| CKMT2 |  |
| CLPB |  |
| CLPP |  |
| CLYBL |  |
| CMC2 |  |
| CMC4 |  |
| CMPK2 |  |
| COA1 |  |
| COA6 |  |
| COA7 |  |
| COA8 |  |
| COASY |  |
| COMT |  |
| COMTD1 |  |
| COQ2 |  |
| COQ6 |  |
| COQ7 |  |
| COQ8A |  |
| COX10 |  |
| COX14 |  |
| COX15 |  |
| COX16 |  |
| COX5A |  |
| COX5B |  |
| COX6A1 |  |
| COX6A2 |  |
| COX6B1 |  |
| COX6B2 |  |
| COX6C |  |
| COX7A1 |  |
| COX7A2 |  |
| COX7B |  |
| COX7B2 |  |
| COX8A |  |
| COX8C |  |
| CPOX |  |
| CPT1A |  |
| CPT2 |  |
| CRAT |  |
| CRLS1 |  |
| CROT |  |
| CRY1 |  |
| CRYZ |  |
| CS |  |
| CSKMT |  |
| CYB5B |  |
| CYB5R3 |  |
| CYC1 |  |
| CYCS |  |
| CYP11A1 |  |
| CYP11B1 |  |
| CYP11B2 |  |
| CYP24A1 |  |
| CYP27B1 |  |
| D2HGDH |  |
| DAP3 |  |
| DARS2 |  |
| DBI |  |
| DCAKD |  |
| DCXR |  |
| DDX28 |  |
| DELE1 |  |
| DGLUCY |  |
| DGUOK |  |
| DHODH |  |
| DHRS1 |  |
| DHRS2 |  |
| DHRS4 |  |
| DHRS7B |  |
| DHTKD1 |  |
| DHX30 |  |
| DIABLO |  |
| DLAT |  |
| DLD |  |
| DLST |  |
| DMAC1 |  |
| DMAC2 |  |
| DMAC2L |  |
| DMGDH |  |
| DMPK |  |
| DNA2 |  |
| DNAJC28 |  |
| DNAJC30 |  |
| DNAJC4 |  |
| DNLZ |  |
| DNM1L |  |
| DTYMK |  |
| DUS2 |  |
| DUT |  |
| ECH1 |  |
| ECHDC1 |  |
| ECHDC3 |  |
| ECI1 |  |
| ECI2 |  |
| ECSIT |  |
| ELAC2 |  |
| ENDOG |  |
| EPHX2 |  |
| ETFA |  |
| ETFRF1 |  |
| ETHE1 |  |
| EXOG |  |
| FABP1 |  |
| FAHD1 |  |
| FAHD2A |  |
| FAM136A |  |
| FAM185A |  |
| FAM210A |  |
| FAM210B |  |
| FARS2 |  |
| FASN |  |
| FASTK |  |
| FASTKD1 |  |
| FASTKD2 |  |
| FDPS |  |
| FDX1 |  |
| FDXR |  |
| FIS1 |  |
| FKBP10 |  |
| FKBP8 |  |
| FLAD1 |  |
| FMC1 |  |
| FOXRED1 |  |
| FTH1 |  |
| GATC |  |
| GATD3A |  |
| GCDH |  |
| GFER |  |
| GFM2 |  |
| GLDC |  |
| GLOD4 |  |
| GLS |  |
| GLS2 |  |
| GLUD1 |  |
| GLYCTK |  |
| GPAM |  |
| GPAT2 |  |
| GPD2 |  |
| GPT2 |  |
| GPX1 |  |
| GPX4 |  |
| GRHPR |  |
| GRPEL2 |  |
| GSR |  |
| GSTK1 |  |
| GTPBP6 |  |
| GUK1 |  |
| HADH |  |
| HADHB |  |
| HAO2 |  |
| HARS2 |  |
| HCCS |  |
| HDHD3 |  |
| HDHD5 |  |
| HEBP1 |  |
| HIBADH |  |
| HIBCH |  |
| HINT1 |  |
| HINT2 |  |
| HINT3 |  |
| HMGCL |  |
| HMGCS2 |  |
| HOGA1 |  |
| HPDL |  |
| HSCB |  |
| HSD17B10 |  |
| HSD17B4 |  |
| HSD17B8 |  |
| HSDL1 |  |
| HSDL2 |  |
| HSPD1 |  |
| HSPE1 |  |
| HTATIP2 |  |
| HTRA2 |  |
| IARS2 |  |
| IDH2 |  |
| IDH3A |  |
| IDH3B |  |
| IDI1 |  |
| ISCA1 |  |
| ISCA2 |  |
| ISOC2 |  |
| IVD |  |
| KARS1 |  |
| L2HGDH |  |
| LAP3 |  |
| LDHB |  |
| LETM1 |  |
| LETM2 |  |
| LETMD1 |  |
| LIAS |  |
| LIG3 |  |
| LIPT2 |  |
| LONP1 |  |
| LYPLA1 |  |
| LYPLAL1 |  |
| LYRM1 |  |
| LYRM2 |  |
| LYRM4 |  |
| LYRM7 |  |
| LYRM9 |  |
| MACROD1 |  |
| MAIP1 |  |
| MAOA |  |
| MAOB |  |
| MARCHF5 |  |
| MARS2 |  |
| MCAT |  |
| MCCC2 |  |
| MCRIP2 |  |
| MCU |  |
| MCUR1 |  |
| MDH2 |  |
| ME3 |  |
| MECR |  |
| METAP1D |  |
| METTL15 |  |
| METTL17 |  |
| METTL4 |  |
| METTL5 |  |
| METTL8 |  |
| MFF |  |
| MFN1 |  |
| MFN2 |  |
| MGME1 |  |
| MGST1 |  |
| MGST3 |  |
| MICU1 |  |
| MICU2 |  |
| MIEF1 |  |
| MIEF2 |  |
| MIGA2 |  |
| MIPEP |  |
| MLYCD |  |
| MMADHC |  |
| MMUT |  |
| MOCS1 |  |
| MPST |  |
| MPV17 |  |
| MPV17L |  |
| MPV17L2 |  |
| MRM1 |  |
| MRM2 |  |
| MRPL1 |  |
| MRPL11 |  |
| MRPL12 |  |
| MRPL13 |  |
| MRPL14 |  |
| MRPL15 |  |
| MRPL16 |  |
| MRPL17 |  |
| MRPL2 |  |
| MRPL22 |  |
| MRPL23 |  |
| MRPL27 |  |
| MRPL28 |  |
| MRPL3 |  |
| MRPL30 |  |
| MRPL33 |  |
| MRPL34 |  |
| MRPL35 |  |
| MRPL36 |  |
| MRPL37 |  |
| MRPL38 |  |
| MRPL39 |  |
| MRPL4 |  |
| MRPL40 |  |
| MRPL41 |  |
| MRPL42 |  |
| MRPL43 |  |
| MRPL44 |  |
| MRPL45 |  |
| MRPL46 |  |
| MRPL47 |  |
| MRPL48 |  |
| MRPL49 |  |
| MRPL50 |  |
| MRPL53 |  |
| MRPL54 |  |
| MRPL55 |  |
| MRPL57 |  |
| MRPL9 |  |
| MRPS10 |  |
| MRPS11 |  |
| MRPS12 |  |
| MRPS14 |  |
| MRPS15 |  |
| MRPS16 |  |
| MRPS18A |  |
| MRPS18C |  |
| MRPS2 |  |
| MRPS21 |  |
| MRPS22 |  |
| MRPS23 |  |
| MRPS24 |  |
| MRPS25 |  |
| MRPS26 |  |
| MRPS27 |  |
| MRPS31 |  |
| MRPS33 |  |
| MRPS5 |  |
| MRPS6 |  |
| MRPS7 |  |
| MRPS9 |  |
| MRRF |  |
| MRS2 |  |
| MSRA |  |
| MSRB3 |  |
| MTARC1 |  |
| MTARC2 |  |
| MT-ATP6 |  |
| MT-ATP8 |  |
| MTCH1 |  |
| MT-CO3 |  |
| MT-CYB |  |
| MTERF1 |  |
| MTFMT |  |
| MTFP1 |  |
| MTFR1 |  |
| MTFR1L |  |
| MTFR2 |  |
| MTG1 |  |
| MTHFD2 |  |
| MTHFD2L |  |
| MTHFS |  |
| MTIF2 |  |
| MTIF3 |  |
| MT-ND1 |  |
| MT-ND2 |  |
| MT-ND3 |  |
| MT-ND4 |  |
| MT-ND4L |  |
| MT-ND6 |  |
| MTPAP |  |
| MTRES1 |  |
| MTRF1 |  |
| MTX1 |  |
| MTX2 |  |
| MUL1 |  |
| MUTYH |  |
| MYG1 |  |
| NADK2 |  |
| NAGS |  |
| NARS2 |  |
| NAT8L |  |
| NBR1 |  |
| NDUFA1 |  |
| NDUFA10 |  |
| NDUFA11 |  |
| NDUFA12 |  |
| NDUFA13 |  |
| NDUFA2 |  |
| NDUFA4 |  |
| NDUFA5 |  |
| NDUFA6 |  |
| NDUFA7 |  |
| NDUFA8 |  |
| NDUFA9 |  |
| NDUFAB1 |  |
| NDUFAF1 |  |
| NDUFAF4 |  |
| NDUFAF6 |  |
| NDUFAF7 |  |
| NDUFAF8 |  |
| NDUFB2 |  |
| NDUFB3 |  |
| NDUFB6 |  |
| NDUFB7 |  |
| NDUFB8 |  |
| NDUFB9 |  |
| NDUFC1 |  |
| NDUFC2 |  |
| NDUFS1 |  |
| NDUFS2 |  |
| NDUFS3 |  |
| NDUFS4 |  |
| NDUFS7 |  |
| NDUFV1 |  |
| NDUFV2 |  |
| NDUFV3 |  |
| NEU4 |  |
| NFU1 |  |
| NGRN |  |
| NIF3L1 |  |
| NIPSNAP3B |  |
| NIT1 |  |
| NIT2 |  |
| NLN |  |
| NLRX1 |  |
| NME3 |  |
| NME4 |  |
| NME6 |  |
| NMNAT3 |  |
| NNT |  |
| NOA1 |  |
| NOCT |  |
| NRDC |  |
| NSUN2 |  |
| NSUN3 |  |
| NSUN4 |  |
| NT5DC2 |  |
| NT5DC3 |  |
| NUBPL |  |
| NUDT19 |  |
| NUDT2 |  |
| NUDT5 |  |
| NUDT6 |  |
| NUDT8 |  |
| NUDT9 |  |
| OAT |  |
| OCIAD1 |  |
| OCIAD2 |  |
| OGDH |  |
| OGDHL |  |
| OGG1 |  |
| OMA1 |  |
| OPA1 |  |
| OPA3 |  |
| OSBPL1A |  |
| OSGEPL1 |  |
| OTC |  |
| OXA1L |  |
| OXCT2 |  |
| OXLD1 |  |
| OXNAD1 |  |
| OXR1 |  |
| OXSM |  |
| PABPC5 |  |
| PAICS |  |
| PARK7 |  |
| PARS2 |  |
| PCBD2 |  |
| PCCA |  |
| PCK2 |  |
| PDE2A |  |
| PDF |  |
| PDHA1 |  |
| PDHA2 |  |
| PDHB |  |
| PDHX |  |
| PDK1 |  |
| PDK4 |  |
| PDP1 |  |
| PDPR |  |
| PDSS1 |  |
| PET117 |  |
| PEX11B |  |
| PGAM5 |  |
| PGS1 |  |
| PHB |  |
| PHB2 |  |
| PHYH |  |
| PICK1 |  |
| PIF1 |  |
| PIGBOS1 |  |
| PINK1 |  |
| PISD |  |
| PITRM1 |  |
| PLD6 |  |
| PLGRKT |  |
| PLPBP |  |
| PLSCR3 |  |
| PMPCA |  |
| PMPCB |  |
| PNPLA8 |  |
| PNPO |  |
| PNPT1 |  |
| POLB |  |
| POLDIP2 |  |
| POLG |  |
| POLG2 |  |
| POLQ |  |
| POLRMT |  |
| PPM1K |  |
| PPOX |  |
| PRDX2 |  |
| PRDX3 |  |
| PRDX4 |  |
| PREPL |  |
| PRKN |  |
| PRODH |  |
| PRODH2 |  |
| PRSS35 |  |
| PRXL2A |  |
| PTCD3 |  |
| PTRH1 |  |
| PTRH2 |  |
| PUS1 |  |
| PUSL1 |  |
| PXMP2 |  |
| PXMP4 |  |
| PYCR1 |  |
| PYURF |  |
| QDPR |  |
| QTRT1 |  |
| RAB24 |  |
| RAB5IF |  |
| RBFA |  |
| RCC1L |  |
| RDH13 |  |
| RDH14 |  |
| RECQL4 |  |
| REXO2 |  |
| RMDN1 |  |
| RMND1 |  |
| RNASEH1 |  |
| ROMO1 |  |
| RP11_469A15.2 |  |
| RPIA |  |
| SAMM50 |  |
| SARDH |  |
| SCO1 |  |
| SCO2 |  |
| SDHAF1 |  |
| SDHC |  |
| SDR39U1 |  |
| SDSL |  |
| SERHL2 |  |
| SETD9 |  |
| SLC25A11 |  |
| SLC25A12 |  |
| SLC25A18 |  |
| SLC25A26 |  |
| SLC25A28 |  |
| SLC25A37 |  |
| SLC25A38 |  |
| SLC25A40 |  |
| SLC25A41 |  |
| SLC25A42 |  |
| SLC25A51 |  |
| SLC25A53 |  |
| SLC30A9 |  |
| SMIM20 |  |
| SMIM8 |  |
| SNAP29 |  |
| SND1 |  |
| SPATA20 |  |
| SPG7 |  |
| SPHK2 |  |
| SPR |  |
| SPRYD4 |  |
| SPTLC2 |  |
| SQOR |  |
| SSBP1 |  |
| STAR |  |
| SUCLA2 |  |
| SUCLG1 |  |
| SYNJ2BP |  |
| TAMM41 |  |
| TARS2 |  |
| TDRKH |  |
| TEFM |  |
| TFAM |  |
| TFB1M |  |
| TFB2M |  |
| THEM4 |  |
| THEM5 |  |
| THG1L |  |
| THNSL1 |  |
| TIMM10 |  |
| TIMM10B |  |
| TIMM13 |  |
| TIMM17A |  |
| TIMM17B |  |
| TIMM23 |  |
| TIMM50 |  |
| TIMM8A |  |
| TIMM8B |  |
| TIMM9 |  |
| TK2 |  |
| TMEM11 |  |
| TMEM126A |  |
| TMEM126B |  |
| TMEM143 |  |
| TMEM14C |  |
| TMEM177 |  |
| TMEM205 |  |
| TMEM65 |  |
| TMEM70 |  |
| TOMM20L |  |
| TOMM22 |  |
| TOMM40 |  |
| TOMM40L |  |
| TOMM5 |  |
| TOMM6 |  |
| TRIT1 |  |
| TRMT1 |  |
| TRMT10C |  |
| TRMT2B |  |
| TSPO |  |
| TST |  |
| TSTD1 |  |
| TSTD3 |  |
| TXN2 |  |
| TXNRD1 |  |
| TXNRD2 |  |
| UCP3 |  |
| UNG |  |
| UQCC2 |  |
| UQCRC1 |  |
| USP30 |  |
| VARS2 |  |
| VDAC2 |  |
| WARS2 |  |
| XPNPEP3 |  |
| YARS2 |  |
| YBEY |  |
| YRDC |  |
| ZADH2 |  |
