# Supplementary material for: Multi-omics analyses related to mitochondria and ageing in triple-negative breast cancer implicate PYCR1 potentiates tumor progression
Source: Cancer Cell Int. 2026 Feb 26;26:150. doi: 10.1186/s12935-026-04235-0 (PMC13041056; doi:10.1186/s12935-026-04235-0)
Supplement: Supplementary file 3 — Supplementary Material 3 [file 12935_2026_4235_MOESM3_ESM.docx]

**Table S4：** Baseline Characteristics of High- and Low-Risk Subgroups

| **Clinical parameters** | High-risk(n=95) | Low-risk(n=96) | statistic | p-value |
| --- | --- | --- | --- | --- |
| **Age** |  |  | 0.544 | 0.461 |
| ≤65 | 72 (75.79%) | 77 (80.21%) |  |  |
| ＞65 | 23 (24.21%) | 19 (19.79%) |  |  |
| **Stage** |  |  | - | 0.314 |
| Stage I | 11 (11.58%) | 17 (17.71%) |  |  |
| Stage II | 63 (66.32%) | 67 (69.79%) |  |  |
| Stage III | 17 (17.89%) | 9 (9.38%) |  |  |
| Stage IV | 2 (2.11%) | 1 (1.04%) |  |  |
| Stage X | 0 (0%) | 1 (1.04%) |  |  |
| Unknown | 2 (2.11%) | 1 (1.04%) |  |  |
| **T.Stage** |  |  | - | 0.363 |
| T1 | 17 (17.89%) | 24 (25%) |  |  |
| T2 | 64 (67.37%) | 62 (64.58%) |  |  |
| T3 | 10 (10.53%) | 8 (8.33%) |  |  |
| T4 | 4 (4.21%) | 1 (1.04%) |  |  |
| TX | 0 (0%) | 1 (1.04%) |  |  |
| **N.Stage** |  |  | 7.979 | 0.046 |
| N0 | 56 (58.95%) | 67 (69.79%) |  |  |
| N1 | 23 (24.21%) | 23 (23.96%) |  |  |
| N2 | 10 (10.53%) | 6 (6.25%) |  |  |
| N3 | 6 (6.32%) | 0 (0%) |  |  |
| **M.Stage** |  |  | 0.925 | 0.630 |
| M0 | 81 (85.26%) | 79 (82.29%) |  |  |
| M1 | 2 (2.11%) | 1 (1.04%) |  |  |
| MX | 12 (12.63%) | 16 (16.67%) |  |  |
