# Supplementary material for: Multi-omics analyses related to mitochondria and ageing in triple-negative breast cancer implicate PYCR1 potentiates tumor progression
Source: Cancer Cell Int. 2026 Feb 26;26:150. doi: 10.1186/s12935-026-04235-0 (PMC13041056; doi:10.1186/s12935-026-04235-0)
Supplement: Supplementary file 4 — Supplementary Material 4 [file 12935_2026_4235_MOESM4_ESM.docx]

**Table S5：** Baseline Characteristics of High- and Low-PYCR1 Expression Subgroups

| **Clinical parameters** | High-PYCR1(n=95) | Low-PYCR1(n=96) | statistic | p-value |
| --- | --- | --- | --- | --- |
| **Age** |  |  | 2.062 | 0.151 |
| ≤65 | 70 (73.68%) | 79 (82.29%) |  |  |
| ＞65 | 25 (26.32%) | 17 (17.71%) |  |  |
| **Stage** |  |  | - | 0.930 |
| Stage I | 13 (13.68%) | 15 (15.62%) |  |  |
| Stage II | 65 (68.42%) | 65 (67.71%) |  |  |
| Stage III | 12 (12.63%) | 14 (14.58%) |  |  |
| Stage IV | 2 (2.11%) | 1 (1.04%) |  |  |
| Stage X | 1 (1.05%) | 0 (0%) |  |  |
| Unknown | 2 (2.11%) | 1 (1.04%) |  |  |
| **T.Stage** |  |  | - | 0.787 |
| T1 | 21 (22.11%) | 20 (20.83%) |  |  |
| T2 | 64 (67.37%) | 62 (64.58%) |  |  |
| T3 | 7 (7.37%) | 11 (11.46%) |  |  |
| T4 | 2 (2.11%) | 3 (3.12%) |  |  |
| TX | 1 (1.05%) | 0 (0%) |  |  |
| **N.Stage** |  |  | 3.147 | 0.370 |
| N0 | 58 (61.05%) | 65 (67.71%) |  |  |
| N1 | 24 (25.26%) | 22 (22.92%) |  |  |
| N2 | 8 (8.42%) | 8 (8.33%) |  |  |
| N3 | 5 (5.26%) | 1 (1.04%) |  |  |
| **M.Stage** |  |  | 1.125 | 0.570 |
| M0 | 77 (81.05%) | 83 (86.46%) |  |  |
| M1 | 2 (2.11%) | 1 (1.04%) |  |  |
| MX | 16 (16.84%) | 12 (12.5%) |  |  |
