# Supplementary material for: Multi-omics analyses related to mitochondria and ageing in triple-negative breast cancer implicate PYCR1 potentiates tumor progression
Source: Cancer Cell Int. 2026 Feb 26;26:150. doi: 10.1186/s12935-026-04235-0 (PMC13041056; doi:10.1186/s12935-026-04235-0)
Supplement: Supplementary file 5 — Supplementary Material 5 [file 12935_2026_4235_MOESM5_ESM.docx]

**Table S7** **:** Gene Lists of the Greenyellow and Purple Modules

| **Probes** | **ModuleColor** |
| --- | --- |
| TNMD | greenyellow |
| HSPB6 | greenyellow |
| PDK4 | greenyellow |
| IGF1 | greenyellow |
| FHL1 | greenyellow |
| C6 | greenyellow |
| NNAT | greenyellow |
| ACACB | greenyellow |
| LIPE | greenyellow |
| CHRDL1 | greenyellow |
| HAS1 | greenyellow |
| RASD1 | greenyellow |
| PRG4 | greenyellow |
| NR4A3 | greenyellow |
| KCNIP2 | greenyellow |
| DUSP1 | greenyellow |
| EGR1 | greenyellow |
| EGR2 | greenyellow |
| ITIH5 | greenyellow |
| NR4A1 | greenyellow |
| ACVR1C | greenyellow |
| G0S2 | greenyellow |
| FOSB | greenyellow |
| ZFP36 | greenyellow |
| CDO1 | greenyellow |
| SLC7A10 | greenyellow |
| AOC3 | greenyellow |
| LGALS12 | greenyellow |
| KLB | greenyellow |
| CD36 | greenyellow |
| SLC19A3 | greenyellow |
| RBP4 | greenyellow |
| ABCA8 | greenyellow |
| CSRNP1 | greenyellow |
| ALDH1L1 | greenyellow |
| NR4A2 | greenyellow |
| TSPAN7 | greenyellow |
| TIMP4 | greenyellow |
| CD300LG | greenyellow |
| ATF3 | greenyellow |
| PI16 | greenyellow |
| VEGFD | greenyellow |
| AQP7 | greenyellow |
| BTNL9 | greenyellow |
| ADAMTS15 | greenyellow |
| PLIN1 | greenyellow |
| GPD1 | greenyellow |
| PLIN4 | greenyellow |
| TNXB | greenyellow |
| FABP4 | greenyellow |
| FOS | greenyellow |
| JUNB | greenyellow |
| TMEM37 | greenyellow |
| ADCY5 | greenyellow |
| LEP | greenyellow |
| LPL | greenyellow |
| CIDEA | greenyellow |
| LRRN4CL | greenyellow |
| JUN | greenyellow |
| EGR3 | greenyellow |
| ADIPOQ | greenyellow |
| TRARG1 | greenyellow |
| CIDEC | greenyellow |
| PLA2G2A | greenyellow |
| ADH1B | greenyellow |
| CFD | greenyellow |
| CES1 | greenyellow |
| GPX3 | greenyellow |
| MYMX | greenyellow |
| ZC3H3 | purple |
| ATP1B3 | purple |
| CPSF1 | purple |
| GFUS | purple |
| PYCR3 | purple |
| SLURP1 | purple |
| VGF | purple |
| THEM6 | purple |
| SLC52A1 | purple |
| ADCYAP1 | purple |
| ADAM15 | purple |
| SRPRB | purple |
| RGS20 | purple |
| ARHGAP39 | purple |
| SLC39A4 | purple |
| RHPN1 | purple |
| LY6K | purple |
| TONSL | purple |
| RECQL4 | purple |
| RELL2 | purple |
| EN2 | purple |
| MFSD3 | purple |
| KIFC2 | purple |
| HSD11B1L | purple |
| COMMD5 | purple |
| HSPA6 | purple |
| ADCK5 | purple |
| PPFIA3 | purple |
| EXOSC4 | purple |
| RRS1 | purple |
| CYC1 | purple |
| SHARPIN | purple |
| TIGD5 | purple |
| PUF60 | purple |
| SCRIB | purple |
| FAM83H | purple |
| MAPK15 | purple |
| ZNF707 | purple |
| RAP2B | purple |
| FBXL6 | purple |
| OLIG1 | purple |
| TOP1MT | purple |
| PTP4A3 | purple |
| HSF1 | purple |
| SLC52A2 | purple |
| RGS9BP | purple |
| PLEKHN1 | purple |
| CYHR1 | purple |
| GPAA1 | purple |
| MB | purple |
| SPRR2E | purple |
| MROH6 | purple |
| DIO1 | purple |
| HAUS7 | purple |
| TINCR | purple |
| HGH1 | purple |
| SHISA9 | purple |
| PWP2 | purple |
| GLI4 | purple |
| FOXD1 | purple |
| SCX | purple |
| BOP1 | purple |
| RHEX | purple |
| LYNX1-SLURP2 | purple |
| MMD | purple |
| STBD1 | purple |
| CTIF | purple |
| FBLN5 | purple |
| GFRA1 | purple |
| JMJD7-PLA2G4B | purple |
| ST6GALNAC3 | purple |
| SH3BGRL2 | purple |
