# Supplementary material for: Multi-omics analyses related to mitochondria and ageing in triple-negative breast cancer implicate PYCR1 potentiates tumor progression
Source: Cancer Cell Int. 2026 Feb 26;26:150. doi: 10.1186/s12935-026-04235-0 (PMC13041056; doi:10.1186/s12935-026-04235-0)
Supplement: Supplementary file 6 — Supplementary Material 6 [file 12935_2026_4235_MOESM6_ESM.docx]

**Table S2：** Patient Sample Identifiers and Corresponding Groups Across Multiple Databases

| **TCGA** | |
| --- | --- |
| Sample | Group |
| TCGA-A1-A0SK-01A | TNBC |
| TCGA-A1-A0SO-01A | TNBC |
| TCGA-A1-A0SP-01A | TNBC |
| TCGA-A2-A04Q-01A | TNBC |
| TCGA-A2-A04T-01A | TNBC |
| TCGA-A2-A04U-01A | TNBC |
| TCGA-A2-A0CM-01A | TNBC |
| TCGA-A2-A0D0-01A | TNBC |
| TCGA-A2-A0D2-01A | TNBC |
| TCGA-A2-A0EQ-01A | TNBC |
| TCGA-A2-A0SX-01A | TNBC |
| TCGA-A2-A0T0-01A | TNBC |
| TCGA-A2-A0T2-01A | TNBC |
| TCGA-A2-A0YE-01A | TNBC |
| TCGA-A2-A0YM-01A | TNBC |
| TCGA-A2-A25F-01A | TNBC |
| TCGA-A2-A3XS-01A | TNBC |
| TCGA-A2-A3XU-01A | TNBC |
| TCGA-A2-A3XV-01A | TNBC |
| TCGA-A2-A3XX-01A | TNBC |
| TCGA-A2-A3XY-01A | TNBC |
| TCGA-A2-A3Y0-01A | TNBC |
| TCGA-A2-A4RX-01A | TNBC |
| TCGA-A2-A4S1-01A | TNBC |
| TCGA-A7-A0DA-01A | TNBC |
| TCGA-A7-A13D-01A | TNBC |
| TCGA-A7-A13E-01A | TNBC |
| TCGA-A7-A26F-01A | TNBC |
| TCGA-A7-A26G-01A | TNBC |
| TCGA-A7-A26I-01A | TNBC |
| TCGA-A7-A4SD-01A | TNBC |
| TCGA-A7-A4SE-01A | TNBC |
| TCGA-A7-A5ZV-01A | TNBC |
| TCGA-A7-A6VV-01A | TNBC |
| TCGA-A7-A6VW-01A | TNBC |
| TCGA-A7-A6VY-01A | TNBC |
| TCGA-A8-A07C-01A | TNBC |
| TCGA-A8-A07O-01A | TNBC |
| TCGA-A8-A07R-01A | TNBC |
| TCGA-A8-A07U-01A | TNBC |
| TCGA-A8-A08R-01A | TNBC |
| TCGA-A8-A09X-01A | TNBC |
| TCGA-AC-A2BK-01A | TNBC |
| TCGA-AC-A2QH-01A | TNBC |
| TCGA-AC-A2QJ-01A | TNBC |
| TCGA-AC-A5EH-01A | TNBC |
| TCGA-AC-A62X-01A | TNBC |
| TCGA-AC-A6IW-01A | TNBC |
| TCGA-AC-A7VC-01A | TNBC |
| TCGA-AC-A8OQ-01A | TNBC |
| TCGA-AN-A04D-01A | TNBC |
| TCGA-AN-A0AL-01A | TNBC |
| TCGA-AN-A0AR-01A | TNBC |
| TCGA-AN-A0AT-01A | TNBC |
| TCGA-AN-A0FJ-01A | TNBC |
| TCGA-AN-A0FX-01A | TNBC |
| TCGA-AN-A0G0-01A | TNBC |
| TCGA-AN-A0XU-01A | TNBC |
| TCGA-AO-A0J2-01A | TNBC |
| TCGA-AO-A0J4-01A | TNBC |
| TCGA-AO-A0J6-01A | TNBC |
| TCGA-AO-A0JL-01A | TNBC |
| TCGA-AO-A124-01A | TNBC |
| TCGA-AO-A128-01A | TNBC |
| TCGA-AO-A129-01A | TNBC |
| TCGA-AO-A12F-01A | TNBC |
| TCGA-AO-A1KR-01A | TNBC |
| TCGA-AQ-A04J-01A | TNBC |
| TCGA-AQ-A54N-01A | TNBC |
| TCGA-AR-A0TP-01A | TNBC |
| TCGA-AR-A0TS-01A | TNBC |
| TCGA-AR-A0TU-01A | TNBC |
| TCGA-AR-A0U0-01A | TNBC |
| TCGA-AR-A0U4-01A | TNBC |
| TCGA-AR-A1AH-01A | TNBC |
| TCGA-AR-A1AI-01A | TNBC |
| TCGA-AR-A1AJ-01A | TNBC |
| TCGA-AR-A1AQ-01A | TNBC |
| TCGA-AR-A1AR-01A | TNBC |
| TCGA-AR-A1AY-01A | TNBC |
| TCGA-AR-A24Q-01A | TNBC |
| TCGA-AR-A251-01A | TNBC |
| TCGA-AR-A256-01A | TNBC |
| TCGA-AR-A2LR-01A | TNBC |
| TCGA-AR-A5QQ-01A | TNBC |
| TCGA-B6-A0I1-01A | TNBC |
| TCGA-B6-A0I6-01A | TNBC |
| TCGA-B6-A0IJ-01A | TNBC |
| TCGA-B6-A0IQ-01A | TNBC |
| TCGA-B6-A0RE-01A | TNBC |
| TCGA-B6-A0RS-01A | TNBC |
| TCGA-B6-A0RT-01A | TNBC |
| TCGA-B6-A0WX-01A | TNBC |
| TCGA-B6-A3ZX-01A | TNBC |
| TCGA-B6-A400-01A | TNBC |
| TCGA-B6-A402-01A | TNBC |
| TCGA-B6-A409-01A | TNBC |
| TCGA-BH-A0AV-01A | TNBC |
| TCGA-BH-A0B9-01A | TNBC |
| TCGA-BH-A0BG-01A | TNBC |
| TCGA-BH-A0BL-01A | TNBC |
| TCGA-BH-A0BW-01A | TNBC |
| TCGA-BH-A0E0-01A | TNBC |
| TCGA-BH-A0E6-01A | TNBC |
| TCGA-BH-A0WA-01A | TNBC |
| TCGA-BH-A18G-01A | TNBC |
| TCGA-BH-A18T-01A | TNBC |
| TCGA-BH-A18V-01A | TNBC |
| TCGA-BH-A1F0-01A | TNBC |
| TCGA-BH-A1F6-01A | TNBC |
| TCGA-BH-A1FC-01A | TNBC |
| TCGA-BH-A203-01A | TNBC |
| TCGA-BH-A208-01A | TNBC |
| TCGA-BH-A6R9-01A | TNBC |
| TCGA-C8-A12L-01A | TNBC |
| TCGA-C8-A12V-01A | TNBC |
| TCGA-C8-A131-01A | TNBC |
| TCGA-C8-A134-01A | TNBC |
| TCGA-C8-A1HJ-01A | TNBC |
| TCGA-C8-A26X-01A | TNBC |
| TCGA-C8-A27B-01A | TNBC |
| TCGA-D8-A13Z-01A | TNBC |
| TCGA-D8-A142-01A | TNBC |
| TCGA-D8-A143-01A | TNBC |
| TCGA-D8-A147-01A | TNBC |
| TCGA-D8-A1JF-01A | TNBC |
| TCGA-D8-A1JL-01A | TNBC |
| TCGA-D8-A1XK-01A | TNBC |
| TCGA-D8-A1XQ-01A | TNBC |
| TCGA-D8-A1XW-01A | TNBC |
| TCGA-D8-A27F-01A | TNBC |
| TCGA-D8-A27H-01A | TNBC |
| TCGA-D8-A27M-01A | TNBC |
| TCGA-E2-A14N-01A | TNBC |
| TCGA-E2-A14R-01A | TNBC |
| TCGA-E2-A14X-01A | TNBC |
| TCGA-E2-A150-01A | TNBC |
| TCGA-E2-A158-01A | TNBC |
| TCGA-E2-A159-01A | TNBC |
| TCGA-E2-A1AZ-01A | TNBC |
| TCGA-E2-A1B6-01A | TNBC |
| TCGA-E2-A1II-01A | TNBC |
| TCGA-E2-A1LG-01A | TNBC |
| TCGA-E2-A1LH-01A | TNBC |
| TCGA-E2-A1LI-01A | TNBC |
| TCGA-E2-A1LK-01A | TNBC |
| TCGA-E2-A1LL-01A | TNBC |
| TCGA-E2-A1LS-01A | TNBC |
| TCGA-E2-A573-01A | TNBC |
| TCGA-E2-A574-01A | TNBC |
| TCGA-E9-A1N8-01A | TNBC |
| TCGA-E9-A1NC-01A | TNBC |
| TCGA-E9-A1ND-01A | TNBC |
| TCGA-E9-A1RH-01A | TNBC |
| TCGA-E9-A22G-01A | TNBC |
| TCGA-E9-A243-01A | TNBC |
| TCGA-E9-A244-01A | TNBC |
| TCGA-E9-A248-01A | TNBC |
| TCGA-E9-A5FL-01A | TNBC |
| TCGA-EW-A1OV-01A | TNBC |
| TCGA-EW-A1OW-01A | TNBC |
| TCGA-EW-A1P4-01A | TNBC |
| TCGA-EW-A1P8-01A | TNBC |
| TCGA-EW-A1PB-01A | TNBC |
| TCGA-EW-A1PH-01A | TNBC |
| TCGA-EW-A3U0-01A | TNBC |
| TCGA-EW-A6SB-01A | TNBC |
| TCGA-GI-A2C9-01A | TNBC |
| TCGA-GM-A2DB-01A | TNBC |
| TCGA-GM-A2DD-01A | TNBC |
| TCGA-GM-A2DF-01A | TNBC |
| TCGA-GM-A3XL-01A | TNBC |
| TCGA-HN-A2NL-01A | TNBC |
| TCGA-JL-A3YW-01A | TNBC |
| TCGA-LL-A441-01A | TNBC |
| TCGA-LL-A5YO-01A | TNBC |
| TCGA-LL-A5YP-01A | TNBC |
| TCGA-LL-A6FR-01A | TNBC |
| TCGA-LL-A73Y-01A | TNBC |
| TCGA-LL-A740-01A | TNBC |
| TCGA-LL-A8F5-01A | TNBC |
| TCGA-OL-A5D6-01A | TNBC |
| TCGA-OL-A5D7-01A | TNBC |
| TCGA-OL-A5RW-01A | TNBC |
| TCGA-OL-A5S0-01A | TNBC |
| TCGA-OL-A66I-01A | TNBC |
| TCGA-OL-A66P-01A | TNBC |
| TCGA-OL-A6VO-01A | TNBC |
| TCGA-PL-A8LZ-01A | TNBC |
| TCGA-S3-AA10-01A | TNBC |
| TCGA-S3-AA15-01A | TNBC |
| TCGA-BH-A0DP-11A | Normal |
| TCGA-E2-A1LB-11A | Normal |
| TCGA-BH-A1FU-11A | Normal |
| TCGA-E9-A1ND-11A | Normal |
| TCGA-A7-A0DB-11A | Normal |
| TCGA-BH-A0DK-11A | Normal |
| TCGA-E9-A1RF-11A | Normal |
| TCGA-BH-A18R-11A | Normal |
| TCGA-GI-A2C8-11A | Normal |
| TCGA-BH-A0C0-11A | Normal |
| TCGA-E2-A1IG-11A | Normal |
| TCGA-E9-A1NA-11A | Normal |
| TCGA-AC-A2FF-11A | Normal |
| TCGA-BH-A0BW-11A | Normal |
| TCGA-BH-A0BQ-11A | Normal |
| TCGA-BH-A0DT-11A | Normal |
| TCGA-BH-A18N-11A | Normal |
| TCGA-E9-A1N4-11A | Normal |
| TCGA-A7-A13G-11A | Normal |
| TCGA-AC-A2FB-11A | Normal |
| TCGA-BH-A0DQ-11A | Normal |
| TCGA-BH-A0HA-11A | Normal |
| TCGA-BH-A0BM-11A | Normal |
| TCGA-BH-A1EN-11A | Normal |
| TCGA-BH-A0B5-11A | Normal |
| TCGA-E2-A15I-11A | Normal |
| TCGA-A7-A0DC-11A | Normal |
| TCGA-A7-A0CH-11A | Normal |
| TCGA-BH-A204-11A | Normal |
| TCGA-E2-A1L7-11A | Normal |
| TCGA-BH-A18L-11A | Normal |
| TCGA-BH-A0BZ-11A | Normal |
| TCGA-BH-A209-11A | Normal |
| TCGA-E9-A1N6-11A | Normal |
| TCGA-BH-A1FB-11A | Normal |
| TCGA-E9-A1RI-11A | Normal |
| TCGA-BH-A0BV-11A | Normal |
| TCGA-A7-A0CE-11A | Normal |
| TCGA-BH-A18V-11A | Normal |
| TCGA-BH-A0BS-11A | Normal |
| TCGA-E9-A1NF-11A | Normal |
| TCGA-BH-A0H9-11A | Normal |
| TCGA-BH-A1F2-11A | Normal |
| TCGA-BH-A0B7-11A | Normal |
| TCGA-E9-A1NG-11A | Normal |
| TCGA-BH-A0B8-11A | Normal |
| TCGA-BH-A0BJ-11A | Normal |
| TCGA-BH-A0DG-11A | Normal |
| TCGA-BH-A0DD-11A | Normal |
| TCGA-E9-A1R7-11A | Normal |
| TCGA-BH-A208-11A | Normal |
| TCGA-E9-A1RB-11A | Normal |
| TCGA-BH-A0AU-11A | Normal |
| TCGA-E9-A1RD-11A | Normal |
| TCGA-BH-A0E1-11A | Normal |
| TCGA-E9-A1RH-11A | Normal |
| TCGA-BH-A0DO-11A | Normal |
| TCGA-BH-A0C3-11A | Normal |
| TCGA-E9-A1RC-11A | Normal |
| TCGA-BH-A1EV-11A | Normal |
| TCGA-BH-A1FC-11A | Normal |
| TCGA-BH-A0E0-11A | Normal |
| TCGA-E2-A1BC-11A | Normal |
| TCGA-BH-A18Q-11A | Normal |
| TCGA-E2-A15M-11A | Normal |
| TCGA-A7-A13E-11A | Normal |
| TCGA-BH-A0DH-11A | Normal |
| TCGA-E2-A1LS-11A | Normal |
| TCGA-BH-A0AY-11A | Normal |
| TCGA-BH-A0DV-11A | Normal |
| TCGA-BH-A0H5-11A | Normal |
| TCGA-BH-A18M-11A | Normal |
| TCGA-BH-A203-11A | Normal |
| TCGA-E9-A1N5-11A | Normal |
| TCGA-BH-A18J-11A | Normal |
| TCGA-E9-A1N9-11A | Normal |
| TCGA-BH-A0HK-11A | Normal |
| TCGA-E2-A158-11A | Normal |
| TCGA-BH-A0BC-11A | Normal |
| TCGA-BH-A0BA-11A | Normal |
| TCGA-BH-A1EU-11A | Normal |
| TCGA-GI-A2C9-11A | Normal |
| TCGA-BH-A1FN-11A | Normal |
| TCGA-BH-A0DL-11A | Normal |
| TCGA-BH-A18U-11A | Normal |
| TCGA-BH-A0BT-11A | Normal |
| TCGA-BH-A18K-11A | Normal |
| TCGA-BH-A0AZ-11A | Normal |
| TCGA-A7-A13F-11A | Normal |
| TCGA-A7-A0D9-11A | Normal |
| TCGA-BH-A18P-11A | Normal |
| TCGA-E2-A1LH-11A | Normal |
| TCGA-E2-A153-11A | Normal |
| TCGA-BH-A18S-11A | Normal |
| TCGA-BH-A0DZ-11A | Normal |
| TCGA-BH-A0H7-11A | Normal |
| TCGA-E2-A15K-11A | Normal |
| TCGA-BH-A1EO-11A | Normal |
| TCGA-AC-A23H-11A | Normal |
| **Metabric** | |
| Sample | Group |
| MB_0035 | TNBC |
| MB_0115 | TNBC |
| MB_0157 | TNBC |
| MB_0158 | TNBC |
| MB_0164 | TNBC |
| MB_0206 | TNBC |
| MB_0209 | TNBC |
| MB_0214 | TNBC |
| MB_0220 | TNBC |
| MB_0221 | TNBC |
| MB_0238 | TNBC |
| MB_0249 | TNBC |
| MB_0292 | TNBC |
| MB_0294 | TNBC |
| MB_0316 | TNBC |
| MB_0352 | TNBC |
| MB_0354 | TNBC |
| MB_0372 | TNBC |
| MB_0400 | TNBC |
| MB_0401 | TNBC |
| MB_0420 | TNBC |
| MB_0424 | TNBC |
| MB_0432 | TNBC |
| MB_0446 | TNBC |
| MB_0453 | TNBC |
| MB_0476 | TNBC |
| MB_0481 | TNBC |
| MB_0494 | TNBC |
| MB_0500 | TNBC |
| MB_0506 | TNBC |
| MB_0516 | TNBC |
| MB_0524 | TNBC |
| MB_0525 | TNBC |
| MB_0540 | TNBC |
| MB_0581 | TNBC |
| MB_0599 | TNBC |
| MB_0608 | TNBC |
| MB_0613 | TNBC |
| MB_0632 | TNBC |
| MB_0635 | TNBC |
| MB_0653 | TNBC |
| MB_0658 | TNBC |
| MB_0659 | TNBC |
| MB_0660 | TNBC |
| MB_0664 | TNBC |
| MB_0869 | TNBC |
| MB_0893 | TNBC |
| MB_0906 | TNBC |
| MB_2556 | TNBC |
| MB_2753 | TNBC |
| MB_2754 | TNBC |
| MB_2827 | TNBC |
| MB_2834 | TNBC |
| MB_2842 | TNBC |
| MB_2849 | TNBC |
| MB_2857 | TNBC |
| MB_2912 | TNBC |
| MB_2917 | TNBC |
| MB_2922 | TNBC |
| MB_2957 | TNBC |
| MB_2963 | TNBC |
| MB_3014 | TNBC |
| MB_3046 | TNBC |
| MB_3057 | TNBC |
| MB_3058 | TNBC |
| MB_3063 | TNBC |
| MB_3067 | TNBC |
| MB_3123 | TNBC |
| MB_3165 | TNBC |
| MB_3271 | TNBC |
| MB_3277 | TNBC |
| MB_3292 | TNBC |
| MB_3367 | TNBC |
| MB_3395 | TNBC |
| MB_3429 | TNBC |
| MB_3453 | TNBC |
| MB_3476 | TNBC |
| MB_3502 | TNBC |
| MB_3567 | TNBC |
| MB_3702 | TNBC |
| MB_3706 | TNBC |
| MB_3752 | TNBC |
| MB_4146 | TNBC |
| MB_4408 | TNBC |
| MB_4622 | TNBC |
| MB_4640 | TNBC |
| MB_4667 | TNBC |
| MB_4712 | TNBC |
| MB_4715 | TNBC |
| MB_4717 | TNBC |
| MB_4732 | TNBC |
| MB_4733 | TNBC |
| MB_4757 | TNBC |
| MB_4758 | TNBC |
| MB_4809 | TNBC |
| MB_4888 | TNBC |
| MB_4911 | TNBC |
| MB_4938 | TNBC |
| MB_4945 | TNBC |
| MB_4982 | TNBC |
| MB_4992 | TNBC |
| MB_4993 | TNBC |
| MB_5019 | TNBC |
| MB_5041 | TNBC |
| MB_5052 | TNBC |
| MB_5058 | TNBC |
| MB_5065 | TNBC |
| MB_5070 | TNBC |
| MB_5072 | TNBC |
| MB_5076 | TNBC |
| MB_5100 | TNBC |
| MB_5109 | TNBC |
| MB_5115 | TNBC |
| MB_5126 | TNBC |
| MB_5137 | TNBC |
| MB_5138 | TNBC |
| MB_5145 | TNBC |
| MB_5157 | TNBC |
| MB_5188 | TNBC |
| MB_5205 | TNBC |
| MB_5209 | TNBC |
| MB_5213 | TNBC |
| MB_5222 | TNBC |
| MB_5223 | TNBC |
| MB_5232 | TNBC |
| MB_5236 | TNBC |
| MB_5258 | TNBC |
| MB_5272 | TNBC |
| MB_5281 | TNBC |
| MB_5294 | TNBC |
| MB_5299 | TNBC |
| MB_5301 | TNBC |
| MB_5323 | TNBC |
| MB_5325 | TNBC |
| MB_5348 | TNBC |
| MB_5378 | TNBC |
| MB_5387 | TNBC |
| MB_5390 | TNBC |
| MB_5392 | TNBC |
| MB_5421 | TNBC |
| MB_5427 | TNBC |
| MB_5442 | TNBC |
| MB_5446 | TNBC |
| MB_5468 | TNBC |
| MB_5482 | TNBC |
| MB_5511 | TNBC |
| MB_5526 | TNBC |
| MB_5529 | TNBC |
| MB_5531 | TNBC |
| MB_5548 | TNBC |
| MB_5551 | TNBC |
| MB_5560 | TNBC |
| MB_5565 | TNBC |
| MB_5602 | TNBC |
| MB_5616 | TNBC |
| MB_5625 | TNBC |
| MB_5634 | TNBC |
| MB_5651 | TNBC |
| MB_0020 | TNBC |
| MB_0045 | TNBC |
| MB_0062 | TNBC |
| MB_0079 | TNBC |
| MB_0100 | TNBC |
| MB_0110 | TNBC |
| MB_0127 | TNBC |
| MB_0149 | TNBC |
| MB_0153 | TNBC |
| MB_0156 | TNBC |
| MB_0160 | TNBC |
| MB_0163 | TNBC |
| MB_0174 | TNBC |
| MB_0179 | TNBC |
| MB_0188 | TNBC |
| MB_0191 | TNBC |
| MB_0200 | TNBC |
| MB_0210 | TNBC |
| MB_0211 | TNBC |
| MB_0241 | TNBC |
| MB_0259 | TNBC |
| MB_0265 | TNBC |
| MB_0269 | TNBC |
| MB_0278 | TNBC |
| MB_0284 | TNBC |
| MB_0289 | TNBC |
| MB_0299 | TNBC |
| MB_0303 | TNBC |
| MB_0318 | TNBC |
| MB_0333 | TNBC |
| MB_0340 | TNBC |
| MB_0350 | TNBC |
| MB_0396 | TNBC |
| MB_0399 | TNBC |
| MB_0403 | TNBC |
| MB_0414 | TNBC |
| MB_0435 | TNBC |
| MB_0436 | TNBC |
| MB_0464 | TNBC |
| MB_0470 | TNBC |
| MB_0489 | TNBC |
| MB_0495 | TNBC |
| MB_0499 | TNBC |
| MB_0502 | TNBC |
| MB_0558 | TNBC |
| MB_0582 | TNBC |
| MB_0588 | TNBC |
| MB_0617 | TNBC |
| MB_0627 | TNBC |
| MB_0634 | TNBC |
| MB_0639 | TNBC |
| MB_0874 | TNBC |
| MB_0876 | TNBC |
| MB_0885 | TNBC |
| MB_0901 | TNBC |
| MB_2643 | TNBC |
| MB_2718 | TNBC |
| MB_2724 | TNBC |
| MB_2771 | TNBC |
| MB_2821 | TNBC |
| MB_2833 | TNBC |
| MB_2850 | TNBC |
| MB_2904 | TNBC |
| MB_2929 | TNBC |
| MB_2993 | TNBC |
| MB_3001 | TNBC |
| MB_3062 | TNBC |
| MB_3153 | TNBC |
| MB_3211 | TNBC |
| MB_3218 | TNBC |
| MB_3297 | TNBC |
| MB_3363 | TNBC |
| MB_3383 | TNBC |
| MB_3396 | TNBC |
| MB_3500 | TNBC |
| MB_4015 | TNBC |
| MB_4024 | TNBC |
| MB_4254 | TNBC |
| MB_4303 | TNBC |
| MB_4332 | TNBC |
| MB_4351 | TNBC |
| MB_4354 | TNBC |
| MB_4407 | TNBC |
| MB_4416 | TNBC |
| MB_4417 | TNBC |
| MB_4621 | TNBC |
| MB_4660 | TNBC |
| MB_4679 | TNBC |
| MB_4696 | TNBC |
| MB_4707 | TNBC |
| MB_4714 | TNBC |
| MB_4769 | TNBC |
| MB_4792 | TNBC |
| MB_4793 | TNBC |
| MB_4859 | TNBC |
| MB_4865 | TNBC |
| MB_4880 | TNBC |
| MB_4881 | TNBC |
| MB_4893 | TNBC |
| MB_4904 | TNBC |
| MB_4928 | TNBC |
| MB_4931 | TNBC |
| MB_4942 | TNBC |
| MB_4974 | TNBC |
| MB_5008 | TNBC |
| MB_5057 | TNBC |
| MB_5102 | TNBC |
| MB_5135 | TNBC |
| MB_5148 | TNBC |
| MB_5155 | TNBC |
| MB_5162 | TNBC |
| MB_5173 | TNBC |
| MB_5208 | TNBC |
| MB_5225 | TNBC |
| MB_5235 | TNBC |
| MB_5255 | TNBC |
| MB_5295 | TNBC |
| MB_5298 | TNBC |
| MB_5335 | TNBC |
| MB_5346 | TNBC |
| MB_5440 | TNBC |
| MB_5450 | TNBC |
| MB_5453 | TNBC |
| MB_5465 | TNBC |
| MB_5547 | TNBC |
| MB_5566 | TNBC |
| MB_5572 | TNBC |
| MB_5577 | TNBC |
| MB_5624 | TNBC |
| MB_5633 | TNBC |
| MB_5655 | TNBC |
| MB_6052 | TNBC |
| MB_6055 | TNBC |
| MB_6058 | TNBC |
| MB_6062 | TNBC |
| MB_6068 | TNBC |
| MB_6098 | TNBC |
| MB_6143 | TNBC |
| MB_6144 | TNBC |
| MB_6152 | TNBC |
| MB_6178 | TNBC |
| MB_6188 | TNBC |
| MB_6223 | TNBC |
| MB_6228 | TNBC |
| MB_6237 | TNBC |
| MB_6242 | TNBC |
| MB_6245 | TNBC |
| MB_6248 | TNBC |
| MB_6251 | TNBC |
| MB_6272 | TNBC |
| MB_6280 | TNBC |
| MB_6305 | TNBC |
| MB_6318 | TNBC |
| MB_6336 | TNBC |
| MB_7007 | TNBC |
| MB_7008 | TNBC |
| MB_7009 | TNBC |
| MB_7012 | TNBC |
| MB_7017 | TNBC |
| MB_7023 | TNBC |
| MB_7025 | TNBC |
| MB_7030 | TNBC |
| MB_7031 | TNBC |
| MB_7036 | TNBC |
| MB_7038 | TNBC |
| MB_7039 | TNBC |
| MB_7045 | TNBC |
| MB_7049 | TNBC |
| MB_7052 | TNBC |
| MB_7054 | TNBC |
| MB_7055 | TNBC |
| MB_7057 | TNBC |
| MB_7066 | TNBC |
| MB_7078 | TNBC |
| MB_7081 | TNBC |
| MB_7087 | TNBC |
| MB_7089 | TNBC |
| MB_7090 | TNBC |
| MB_7114 | TNBC |
| MB_7119 | TNBC |
| MB_7121 | TNBC |
| MB_7145 | TNBC |
| MB_7151 | TNBC |
| MB_7154 | TNBC |
| MB_7155 | TNBC |
| MB_7158 | TNBC |
| MB_7159 | TNBC |
| MB_7165 | TNBC |
| MB_7201 | TNBC |
| MB_7205 | TNBC |
| MB_7208 | TNBC |
| MB_7225 | TNBC |
| MB_7252 | TNBC |
| MB_7258 | TNBC |
| MB_7267 | TNBC |
| MB_7269 | TNBC |
| MB_7270 | TNBC |
| **GSE58812** | |
| Sample | Group |
| GSM1419942 | TNBC |
| GSM1419943 | TNBC |
| GSM1419944 | TNBC |
| GSM1419945 | TNBC |
| GSM1419946 | TNBC |
| GSM1419947 | TNBC |
| GSM1419948 | TNBC |
| GSM1419949 | TNBC |
| GSM1419950 | TNBC |
| GSM1419951 | TNBC |
| GSM1419952 | TNBC |
| GSM1419953 | TNBC |
| GSM1419954 | TNBC |
| GSM1419955 | TNBC |
| GSM1419956 | TNBC |
| GSM1419957 | TNBC |
| GSM1419958 | TNBC |
| GSM1419968 | TNBC |
| GSM1419969 | TNBC |
| GSM1419970 | TNBC |
| GSM1419971 | TNBC |
| GSM1419972 | TNBC |
| GSM1419973 | TNBC |
| GSM1419974 | TNBC |
| GSM1419975 | TNBC |
| GSM1419976 | TNBC |
| GSM1419977 | TNBC |
| GSM1419978 | TNBC |
| GSM1419979 | TNBC |
| GSM1419980 | TNBC |
| GSM1419981 | TNBC |
| GSM1419982 | TNBC |
| GSM1419983 | TNBC |
| GSM1419984 | TNBC |
| GSM1419985 | TNBC |
| GSM1419986 | TNBC |
| GSM1419987 | TNBC |
| GSM1419988 | TNBC |
| GSM1419989 | TNBC |
| GSM1419990 | TNBC |
| GSM1419991 | TNBC |
| GSM1419992 | TNBC |
| GSM1419993 | TNBC |
| GSM1419994 | TNBC |
| GSM1419995 | TNBC |
| GSM1419996 | TNBC |
| GSM1419997 | TNBC |
| GSM1419998 | TNBC |
| GSM1419999 | TNBC |
| GSM1420000 | TNBC |
| GSM1420001 | TNBC |
| GSM1420002 | TNBC |
| GSM1420003 | TNBC |
| GSM1420004 | TNBC |
| GSM1420005 | TNBC |
| GSM1420006 | TNBC |
| GSM1420007 | TNBC |
| GSM1420008 | TNBC |
| GSM1420009 | TNBC |
| GSM1420010 | TNBC |
| GSM1420011 | TNBC |
| GSM1420012 | TNBC |
| GSM1420013 | TNBC |
| GSM1420014 | TNBC |
| GSM1420015 | TNBC |
| GSM1420016 | TNBC |
| GSM1420017 | TNBC |
| GSM1420018 | TNBC |
| GSM1420019 | TNBC |
| GSM1420020 | TNBC |
| GSM1420021 | TNBC |
| GSM1420022 | TNBC |
| GSM1420023 | TNBC |
| GSM1420024 | TNBC |
| GSM1420025 | TNBC |
| GSM1420026 | TNBC |
| GSM1420027 | TNBC |
| GSM1420028 | TNBC |
| GSM1420029 | TNBC |
| GSM1420030 | TNBC |
| GSM1420031 | TNBC |
| GSM1420032 | TNBC |
| GSM1420033 | TNBC |
| GSM1420034 | TNBC |
| GSM1420035 | TNBC |
| GSM1420036 | TNBC |
| GSM1420037 | TNBC |
| GSM1420038 | TNBC |
| GSM1420039 | TNBC |
| GSM1420040 | TNBC |
| GSM1420041 | TNBC |
| GSM1420042 | TNBC |
| GSM1420043 | TNBC |
| GSM1420044 | TNBC |
| GSM1420045 | TNBC |
| GSM1420046 | TNBC |
| GSM1420047 | TNBC |
| GSM1420048 | TNBC |
| GSM1420049 | TNBC |
| GSM1420050 | TNBC |
| GSM1420051 | TNBC |
| GSM1420052 | TNBC |
| GSM1420053 | TNBC |
| GSM1420054 | TNBC |
| GSM1420055 | TNBC |
| GSM1420056 | TNBC |
| GSM1420057 | TNBC |
| **GSE76275** | |
| Sample | Group |
| GSM1974566 | TNBC |
| GSM1974567 | TNBC |
| GSM1974568 | TNBC |
| GSM1974569 | TNBC |
| GSM1974570 | TNBC |
| GSM1974571 | TNBC |
| GSM1974572 | TNBC |
| GSM1974573 | TNBC |
| GSM1974574 | TNBC |
| GSM1974575 | TNBC |
| GSM1974576 | TNBC |
| GSM1974577 | TNBC |
| GSM1974578 | TNBC |
| GSM1974579 | TNBC |
| GSM1974580 | TNBC |
| GSM1974581 | TNBC |
| GSM1974582 | TNBC |
| GSM1974583 | TNBC |
| GSM1974584 | TNBC |
| GSM1974585 | TNBC |
| GSM1974586 | TNBC |
| GSM1974587 | TNBC |
| GSM1974588 | TNBC |
| GSM1974589 | TNBC |
| GSM1974590 | TNBC |
| GSM1974591 | TNBC |
| GSM1974592 | TNBC |
| GSM1974593 | TNBC |
| GSM1974594 | TNBC |
| GSM1974595 | TNBC |
| GSM1974596 | TNBC |
| GSM1974597 | TNBC |
| GSM1974598 | TNBC |
| GSM1974599 | TNBC |
| GSM1974600 | TNBC |
| GSM1974601 | TNBC |
| GSM1974602 | TNBC |
| GSM1974603 | TNBC |
| GSM1974604 | TNBC |
| GSM1974605 | TNBC |
| GSM1974606 | TNBC |
| GSM1974607 | TNBC |
| GSM1974608 | TNBC |
| GSM1974609 | TNBC |
| GSM1974610 | TNBC |
| GSM1974611 | TNBC |
| GSM1974612 | TNBC |
| GSM1974613 | TNBC |
| GSM1974614 | TNBC |
| GSM1974615 | TNBC |
| GSM1974616 | TNBC |
| GSM1974617 | TNBC |
| GSM1974618 | TNBC |
| GSM1974619 | TNBC |
| GSM1974620 | TNBC |
| GSM1974621 | TNBC |
| GSM1974622 | TNBC |
| GSM1974623 | TNBC |
| GSM1974624 | TNBC |
| GSM1974625 | TNBC |
| GSM1974626 | TNBC |
| GSM1974627 | TNBC |
| GSM1974628 | TNBC |
| GSM1974629 | TNBC |
| GSM1974630 | TNBC |
| GSM1974631 | TNBC |
| GSM1974632 | TNBC |
| GSM1974633 | TNBC |
| GSM1974634 | TNBC |
| GSM1974635 | TNBC |
| GSM1974636 | TNBC |
| GSM1974637 | TNBC |
| GSM1974638 | TNBC |
| GSM1974639 | TNBC |
| GSM1974640 | TNBC |
| GSM1974641 | TNBC |
| GSM1974642 | TNBC |
| GSM1974643 | TNBC |
| GSM1974644 | TNBC |
| GSM1974645 | TNBC |
| GSM1974646 | TNBC |
| GSM1974647 | TNBC |
| GSM1974648 | TNBC |
| GSM1974649 | TNBC |
| GSM1974650 | TNBC |
| GSM1974651 | TNBC |
| GSM1974652 | TNBC |
| GSM1974653 | TNBC |
| GSM1974654 | TNBC |
| GSM1974655 | TNBC |
| GSM1974656 | TNBC |
| GSM1974657 | TNBC |
| GSM1974658 | TNBC |
| GSM1974659 | TNBC |
| GSM1974660 | TNBC |
| GSM1974661 | TNBC |
| GSM1974662 | TNBC |
| GSM1974663 | TNBC |
| GSM1974664 | TNBC |
| GSM1974665 | TNBC |
| GSM1974666 | TNBC |
| GSM1974667 | TNBC |
| GSM1974668 | TNBC |
| GSM1974669 | TNBC |
| GSM1974670 | TNBC |
| GSM1974671 | TNBC |
| GSM1974672 | TNBC |
| GSM1974673 | TNBC |
| GSM1974674 | TNBC |
| GSM1974675 | TNBC |
| GSM1974676 | TNBC |
| GSM1974677 | TNBC |
| GSM1974678 | TNBC |
| GSM1974679 | TNBC |
| GSM1974680 | TNBC |
| GSM1974681 | TNBC |
| GSM1974682 | TNBC |
| GSM1974683 | TNBC |
| GSM1974684 | TNBC |
| GSM1974685 | TNBC |
| GSM1974686 | TNBC |
| GSM1974687 | TNBC |
| GSM1974688 | TNBC |
| GSM1974689 | TNBC |
| GSM1974690 | TNBC |
| GSM1974691 | TNBC |
| GSM1974692 | TNBC |
| GSM1974693 | TNBC |
| GSM1974694 | TNBC |
| GSM1974695 | TNBC |
| GSM1974696 | TNBC |
| GSM1974697 | TNBC |
| GSM1974698 | TNBC |
| GSM1974699 | TNBC |
| GSM1974700 | TNBC |
| GSM1974701 | TNBC |
| GSM1974702 | TNBC |
| GSM1974703 | TNBC |
| GSM1974704 | TNBC |
| GSM1974705 | TNBC |
| GSM1974706 | TNBC |
| GSM1974707 | TNBC |
| GSM1974708 | TNBC |
| GSM1974709 | TNBC |
| GSM1974710 | TNBC |
| GSM1974711 | TNBC |
| GSM1974712 | TNBC |
| GSM1974713 | TNBC |
| GSM1974714 | TNBC |
| GSM1974715 | TNBC |
| GSM1974716 | TNBC |
| GSM1974717 | TNBC |
| GSM1974718 | TNBC |
| GSM1974719 | TNBC |
| GSM1974720 | TNBC |
| GSM1974721 | TNBC |
| GSM1974722 | TNBC |
| GSM1974723 | TNBC |
| GSM1974724 | TNBC |
| GSM1974725 | TNBC |
| GSM1974726 | TNBC |
| GSM1974727 | TNBC |
| GSM1974728 | TNBC |
| GSM1974729 | TNBC |
| GSM1974730 | TNBC |
| GSM1974731 | TNBC |
| GSM1974732 | TNBC |
| GSM1974733 | TNBC |
| GSM1974734 | TNBC |
| GSM1974735 | TNBC |
| GSM1974736 | TNBC |
| GSM1974737 | TNBC |
| GSM1974738 | TNBC |
| GSM1974739 | TNBC |
| GSM1974740 | TNBC |
| GSM1974741 | TNBC |
| GSM1974742 | TNBC |
| GSM1974743 | TNBC |
| GSM1974744 | TNBC |
| GSM1974745 | TNBC |
| GSM1974746 | TNBC |
| GSM1974747 | TNBC |
| GSM1974748 | TNBC |
| GSM1974749 | TNBC |
| GSM1974750 | TNBC |
| GSM1974751 | TNBC |
| GSM1974752 | TNBC |
| GSM1974753 | TNBC |
| GSM1974754 | TNBC |
| GSM1974755 | TNBC |
| GSM1974756 | TNBC |
| GSM1974757 | TNBC |
| GSM1974758 | TNBC |
| GSM1974759 | TNBC |
| GSM1974760 | TNBC |
| GSM1974761 | TNBC |
| GSM1974762 | TNBC |
| GSM1974763 | TNBC |
| GSM1978883 | non-TNBC |
| GSM1978884 | non-TNBC |
| GSM1978885 | non-TNBC |
| GSM1978886 | non-TNBC |
| GSM1978887 | non-TNBC |
| GSM1978888 | non-TNBC |
| GSM1978889 | non-TNBC |
| GSM1978890 | non-TNBC |
| GSM1978891 | non-TNBC |
| GSM1978892 | non-TNBC |
| GSM1978893 | non-TNBC |
| GSM1978894 | non-TNBC |
| GSM1978895 | non-TNBC |
| GSM1978896 | non-TNBC |
| GSM1978897 | non-TNBC |
| GSM1978898 | non-TNBC |
| GSM1978899 | non-TNBC |
| GSM1978900 | non-TNBC |
| GSM1978901 | non-TNBC |
| GSM1978902 | non-TNBC |
| GSM1978903 | non-TNBC |
| GSM1978904 | non-TNBC |
| GSM1978905 | non-TNBC |
| GSM1978906 | non-TNBC |
| GSM1978907 | non-TNBC |
| GSM1978908 | non-TNBC |
| GSM1978909 | non-TNBC |
| GSM1978910 | non-TNBC |
| GSM1978911 | non-TNBC |
| GSM1978912 | non-TNBC |
| GSM1978913 | non-TNBC |
| GSM1978914 | non-TNBC |
| GSM1978915 | non-TNBC |
| GSM1978916 | non-TNBC |
| GSM1978917 | non-TNBC |
| GSM1978918 | non-TNBC |
| GSM1978919 | non-TNBC |
| GSM1978920 | non-TNBC |
| GSM1978921 | non-TNBC |
| GSM1978922 | non-TNBC |
| GSM1978923 | non-TNBC |
| GSM1978924 | non-TNBC |
| GSM1978925 | non-TNBC |
| GSM1978926 | non-TNBC |
| GSM1978927 | non-TNBC |
| GSM1978928 | non-TNBC |
| GSM1978929 | non-TNBC |
| GSM1978930 | non-TNBC |
| GSM1978931 | non-TNBC |
| GSM1978932 | non-TNBC |
| GSM1978933 | non-TNBC |
| GSM1978934 | non-TNBC |
| GSM1978935 | non-TNBC |
| GSM1978936 | non-TNBC |
| GSM1978937 | non-TNBC |
| GSM1978938 | non-TNBC |
| GSM1978939 | non-TNBC |
| GSM1978940 | non-TNBC |
| GSM1978941 | non-TNBC |
| GSM1978942 | non-TNBC |
| GSM1978943 | non-TNBC |
| GSM1978944 | non-TNBC |
| GSM1978945 | non-TNBC |
| GSM1978946 | non-TNBC |
| GSM1978947 | non-TNBC |
| GSM1978948 | non-TNBC |
| GSM1978949 | non-TNBC |
| **GSE161529** | |
| Sample | Group |
| GSM4909281 | TNBC |
| GSM4909282 | TNBC |
| GSEM4909283 | TNBC |
| GSM4909284 | TNBC |
