# Supplementary figures and images for: Multi-omics analyses related to mitochondria and ageing in triple-negative breast cancer implicate PYCR1 potentiates tumor progression
Source: Cancer Cell Int. 2026 Feb 26;26:150. doi: 10.1186/s12935-026-04235-0 (PMC13041056; doi:10.1186/s12935-026-04235-0)

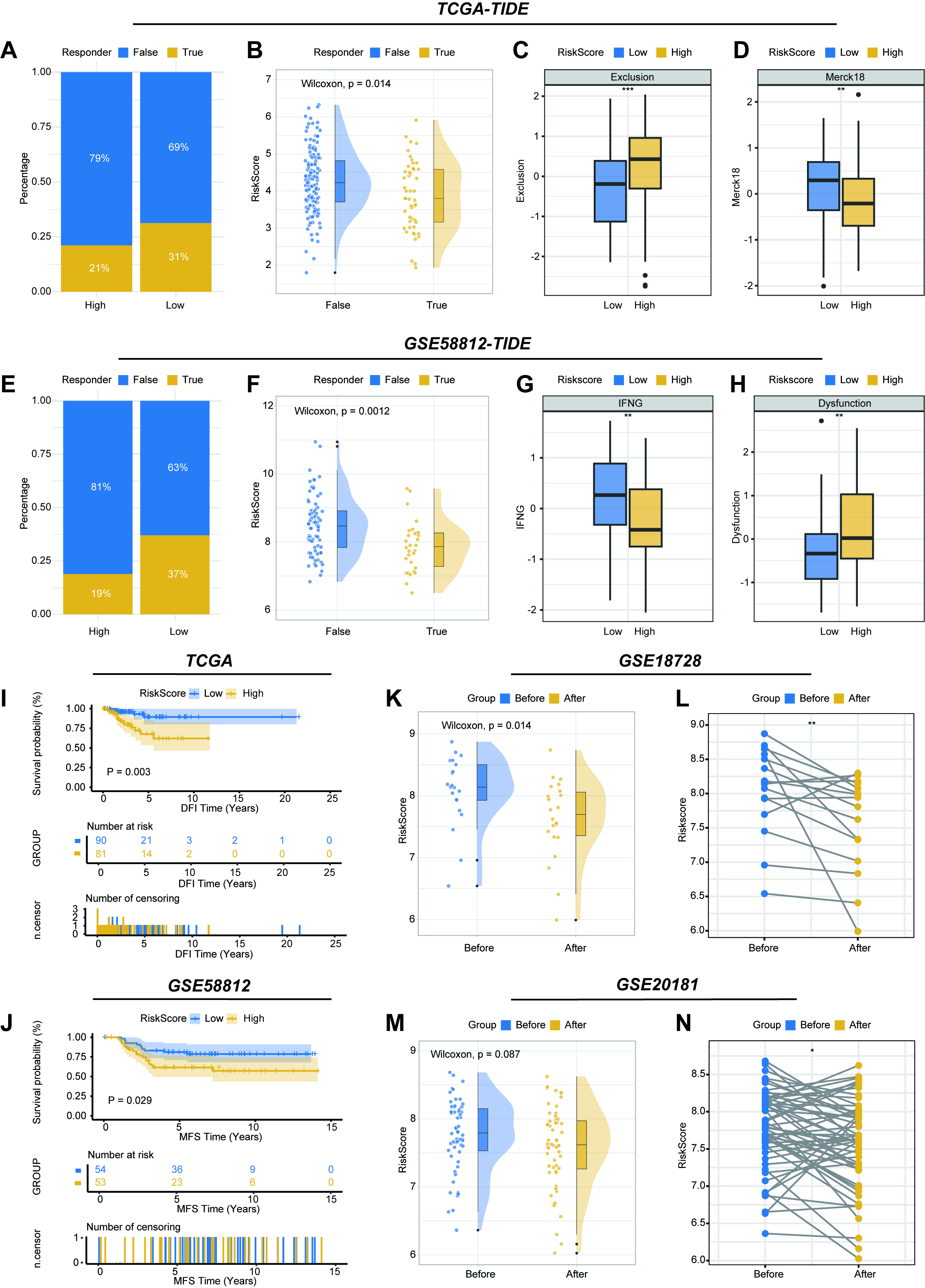

Supplement: Supplementary file 8 — Supplementary Material 8: Figure S1: The therapeutic benefit of the MARS value. A, E: The proportion of predicted immunotherapy responses in high-risk and low-risk subgroups. B, F: MARS of different immune response groups. C, D: Exclusion and Merck18 scores of high-risk and low-risk subgroups within the TCGA cohort. G, H: IFNG and Dysfunction scores of high-risk and low-risk subgroups within the GSE58812 cohort. I: Kaplan-Meier curves for DFI of TNBC patients within the TCGA and GSE58812 cohort, stratified by median MARS. J: Kaplan-Meier curves for MFS of TNBC patients within the GSE58812 cohort, stratified by median MARS. K, M: MARS of pre-chemotherapy and post-chemotherapy subgroups. L, N: Paired MARS of pre-chemotherapy and post-chemotherapy subgroups. [file 12935_2026_4235_MOESM8_ESM.tif]

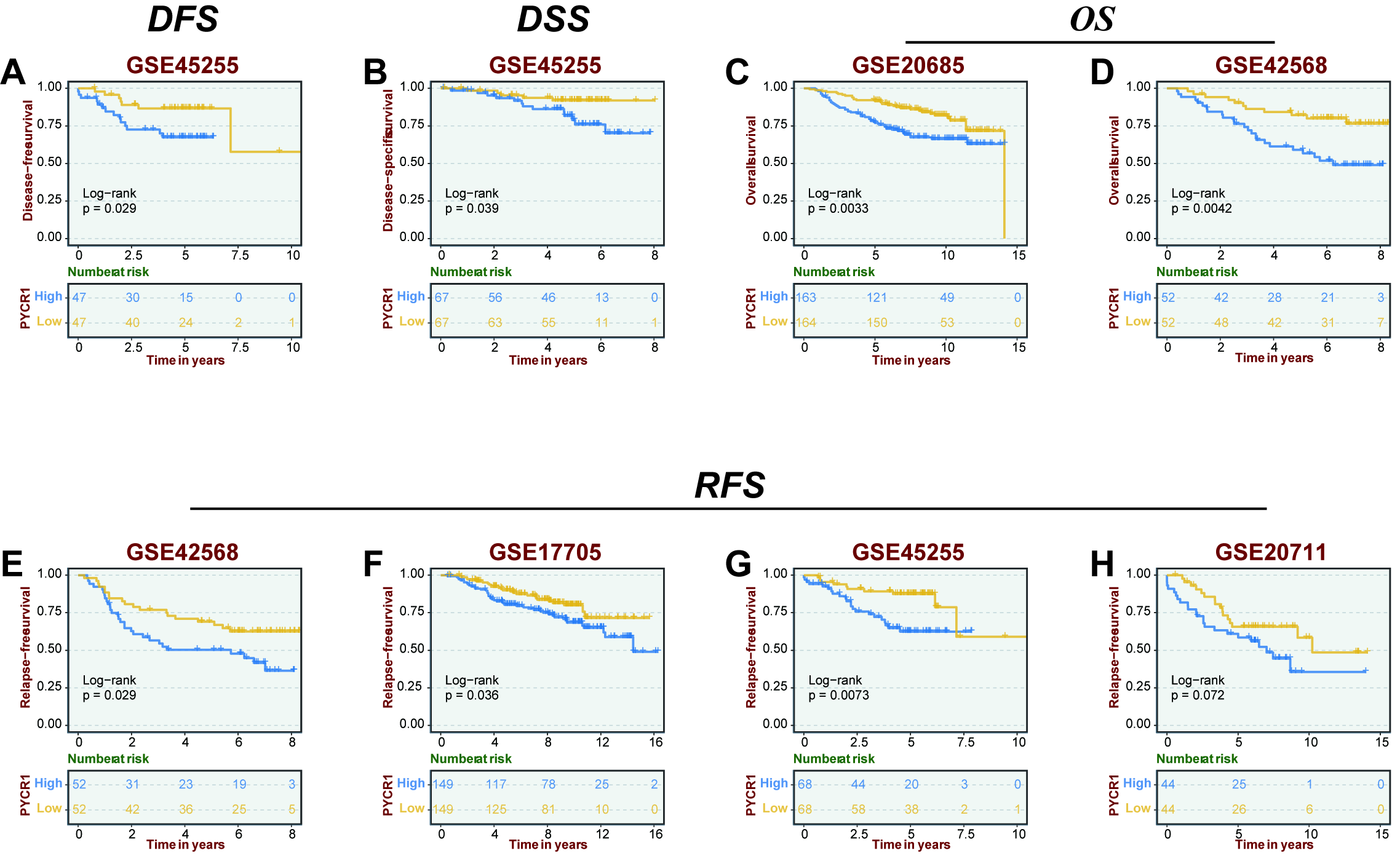

Supplement: Supplementary file 9 — Supplementary Material 9: Figure S2: Comprehensive pan-cancer analysis of PYCR1. A: Pan-cancer expression of PYCR1 in 33 tumor samples (without normal samples). B: Correlation between PYCR1 and the proportions of immune cells across pan-cancer. C: Triangular representation of the correlation between PYCR1 and immune scores across pan-cancer. D: Heatmap of the correlation between PYCR1 and immune scores. E: Pan-cancer Cox regression analysis of PYCR1 in TCGA cancers. F: Radar plot of the correlation between PYCR1 expression and TMB. [file 12935_2026_4235_MOESM9_ESM.tif]

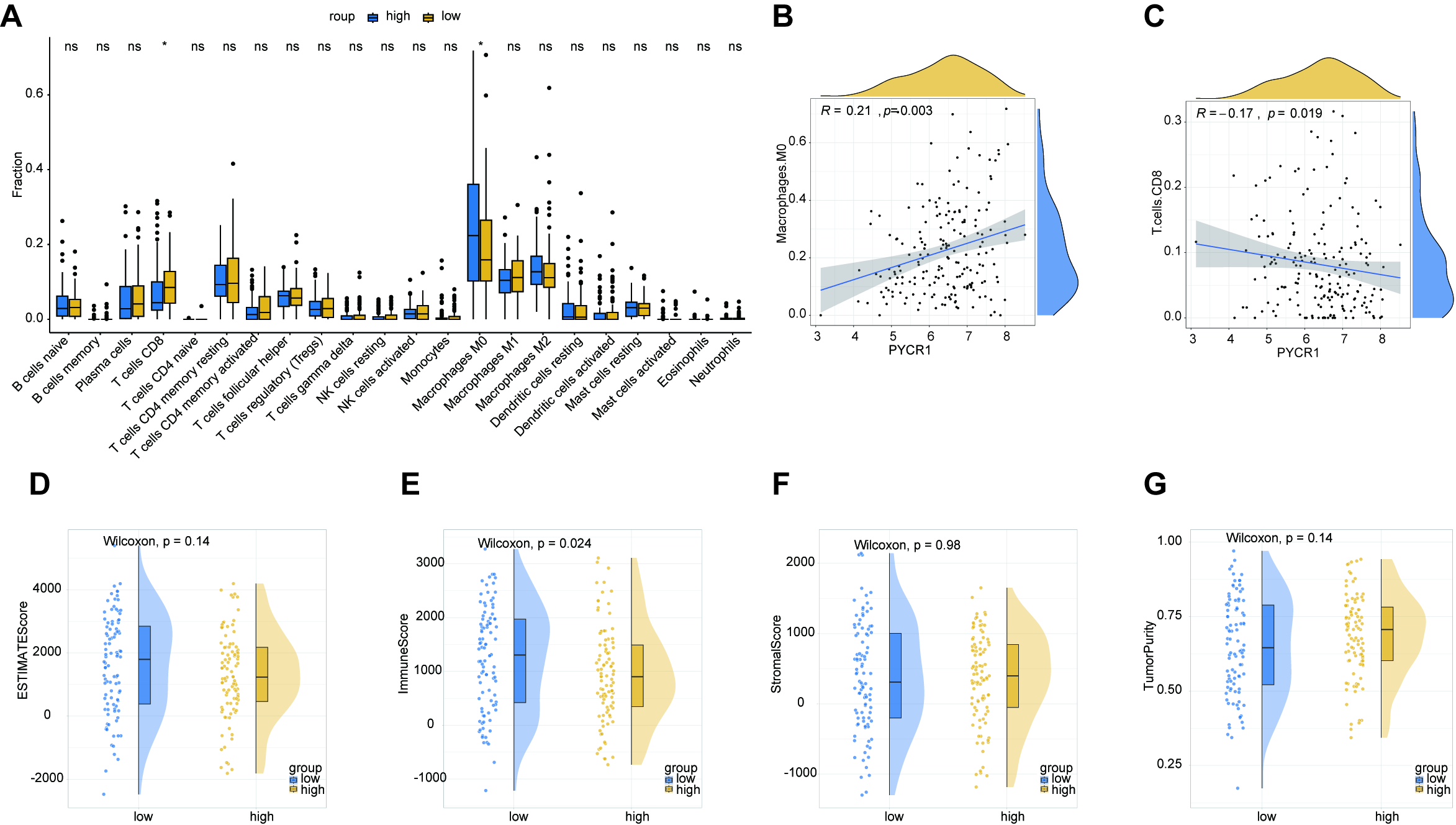

Supplement: Supplementary file 10 — Supplementary Material 10:Figure S3: Survival analysis of PYCR1 in different datasets. A: Kaplan-Meier curve for DFS of BC patients in the GSE45255 dataset. B: Kaplan-Meier curve for DSS of BC patients in the GSE45255 dataset. C: Kaplan-Meier curve for OS of BC patients in the GSE20685 dataset. D: Kaplan-Meier curve for OS of BC patients in the GSE42568 dataset. E: Kaplan-Meier curve for RFS of BC patients in the GSE42568 dataset. F: Kaplan-Meier curve for RFS of BC patients in the GSE17705 dataset. G: Kaplan-Meier curve for RFS of BC patients in the GSE45255 dataset. H: Kaplan-Meier curve for RFS of BC patients in the GSE20711 dataset [file 12935_2026_4235_MOESM10_ESM.tif]

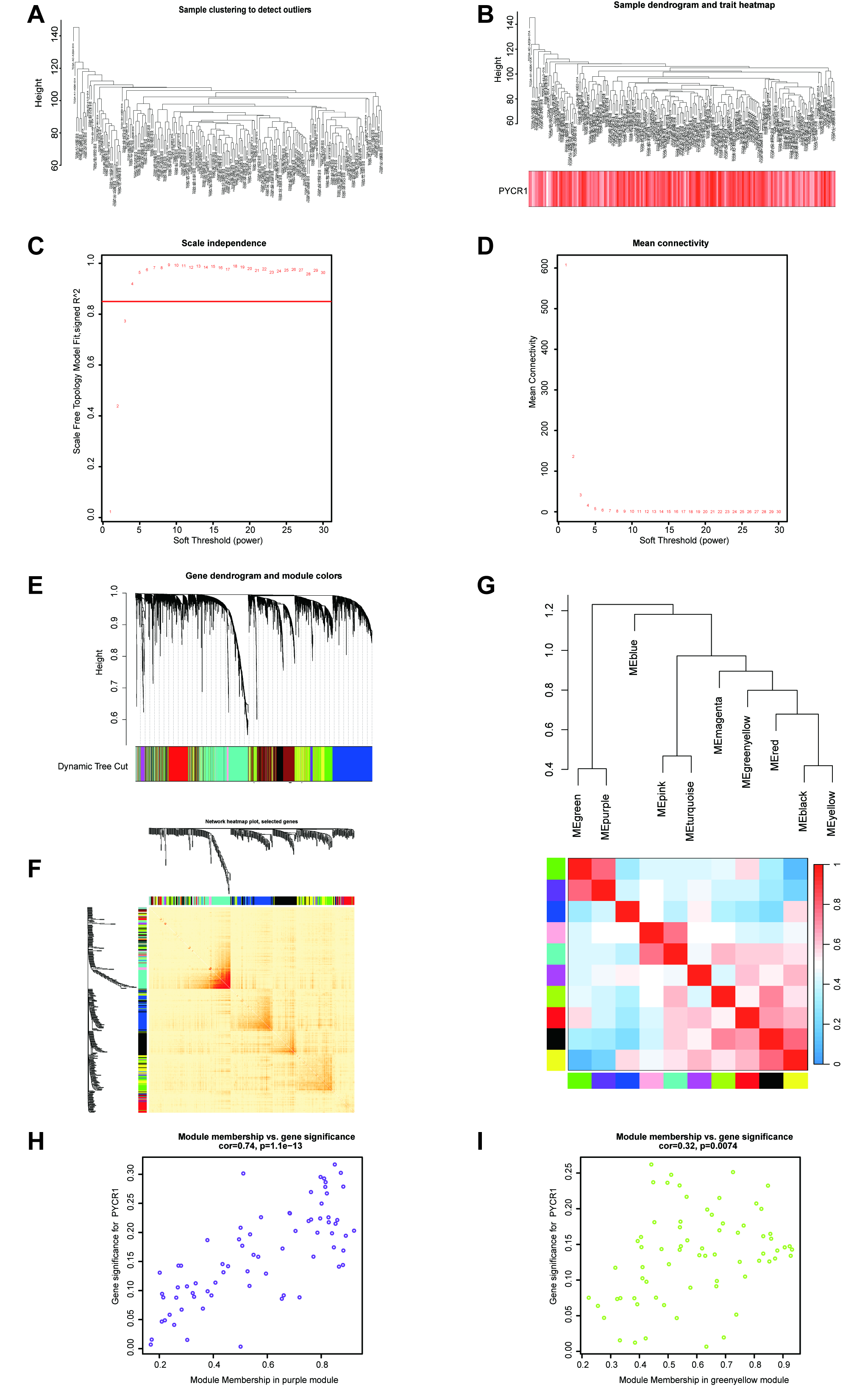

Supplement: Supplementary file 11 — Supplementary Material 11: Figure S4: Immune cell landscape between high and low PYCR1 expression groups in the TCGA-TNBC cohort. A: Expression profiles of immune cells between high and low PYCR1 expression groups. B: Positive correlation between Macrophages M0 and PYCR1 expression. C: Negative correlation between T cells CD8 and PYCR1 expression. D: Stromal score. E: Immune score. F: ESTIMATE score. G: Tumor purity. *p < 0.05, ** p < 0.01, *** p < 0.001. [file 12935_2026_4235_MOESM11_ESM.tif]

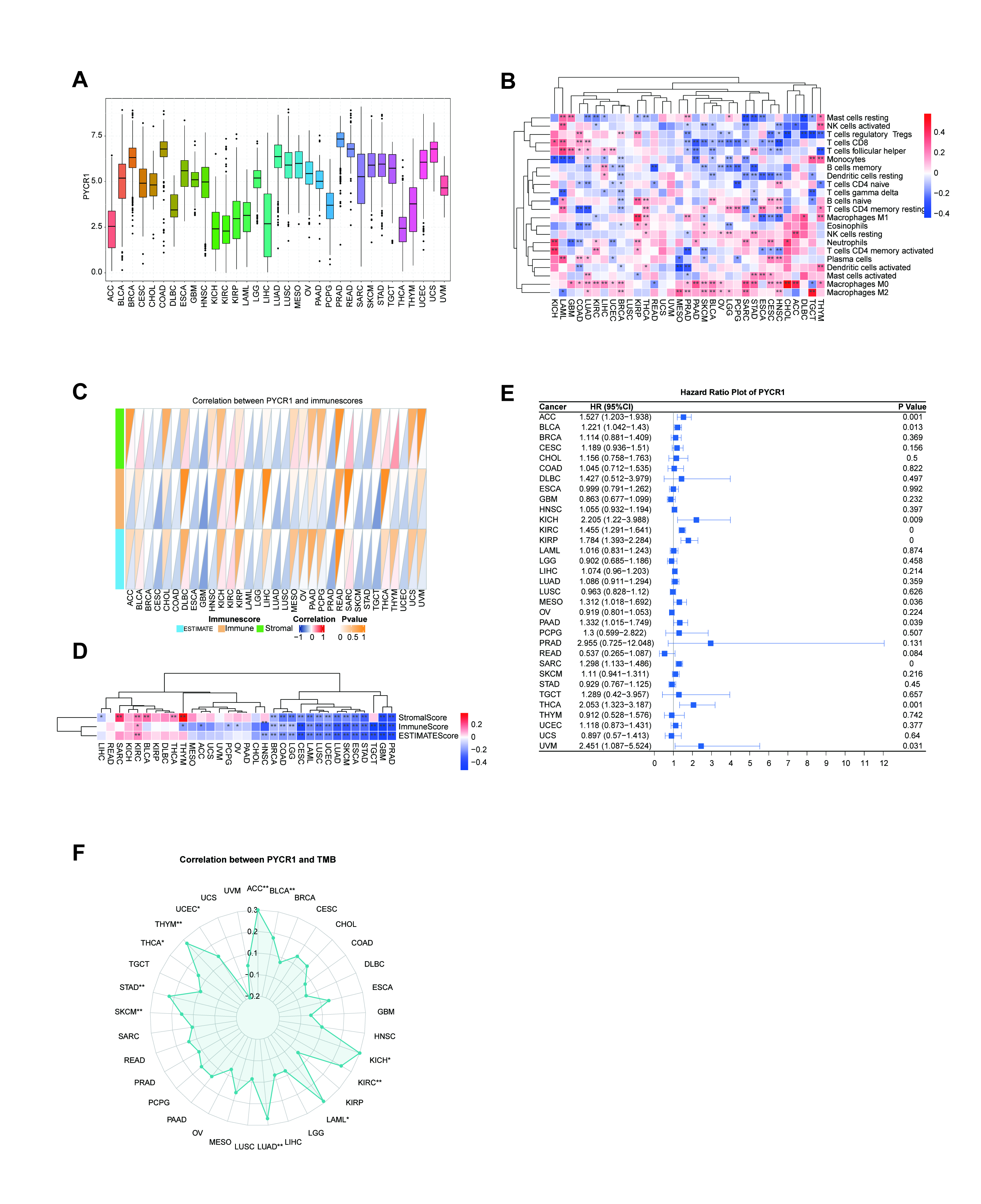

Supplement: Supplementary file 12 — Supplementary Material 12: Figure S5: Identification of gene modules co-expressed with PYCR1 in the TCGA-TNBC cohort using WGCNA. A: Dendrogram of all DEGs based on their trend-adjusted expression profiles. B: Trend-adjusted expression profiles of DEGs, with colors assigned according to their module membership. C,D: Dendrogram of TNBC patients. 6223 genes were clustered into 11 modules based on dissimilarity measure (1-TOM). E: Clustering dendrogram of module eigengenes. F: Heatmap showing the clustering relationship between gene modules and samples. G: Heatmap of correlations between gene modules. H: Scatter plots of the purple module. I: Scatter plots of the purple module. [file 12935_2026_4235_MOESM12_ESM.tif]
